# A plug-and-play approach to antibody-based therapeutics *via* a chemoselective dual click strategy

**DOI:** 10.1038/ncomms7645

**Published:** 2015-03-31

**Authors:** Antoine Maruani, Mark E.B. Smith, Enrique Miranda, Kerry A. Chester, Vijay Chudasama, Stephen Caddick

**Affiliations:** 1 Department of Chemistry, University College London, 20 Gordon Street, London WC1H OAJ, UK; 2 UCL Cancer Institute, 72 Huntley Street, London WC1E 6BT, UK

## Abstract

Although recent methods for the engineering of antibody–drug conjugates (ADCs) have gone some way to addressing the challenging issues of ADC construction, significant hurdles still remain. There is clear demand for the construction of novel ADC platforms that offer greater stability, homogeneity and flexibility. Here we describe a significant step towards a platform for next-generation antibody-based therapeutics by providing constructs that combine site-specific modification, exceptional versatility and high stability, with retention of antibody binding and structure post-modification. The relevance of the work in a biological context is also demonstrated in a cytotoxicity assay and a cell internalization study with HER2-positive and -negative breast cancer cell lines.

## Abstract (web summary)

Antibody–drug conjugates are a class of therapeutic combining the directing ability of antibodies with the cell-killing ability of cytotoxic drugs. Here the authors describe an approach based on click chemistry that enables the rapid assembly of dual-modified antibodies with potential for new therapeutic modalities.

## Graphical abstract

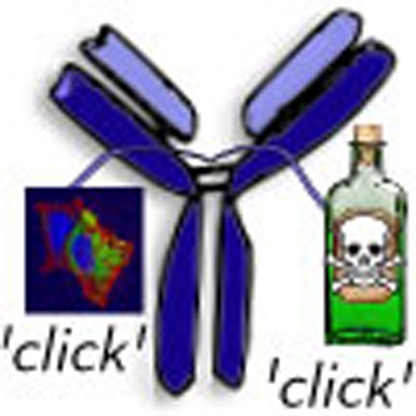

Antibody–drug conjugates (ADCs) are comprised of antibodies that are armed with highly potent warheads using various conjugation/linker technologies^1^,^2^,^3^,^4^. This class of therapeutic combines the directing ability of antibodies (that is, allowing for discrimination between healthy and diseased tissue) with the cell-killing ability of potent cytotoxic drugs. This powerful class of targeted therapy has shown considerable promise in the treatment of various cancers with two US Food and Drug Administration (FDA)-approved ADCs currently on the market (Adcetris and Kadcyla) and over 30 ADCs currently in the clinic^5^,^6^. However, in order for ADCs to deliver their full potential, sophisticated conjugation technologies to connect the warhead to the antibody and novel strategies and approaches for their construction are required^7^,^8^. Conjugation to native ADCs is typically achieved through either multiple lysine modification or by functionalization of thiols generated by reduction of interchain disulfide bonds; neither of which is ideal (Fig. 1)^7^,^8^. Lysine modification is suboptimal as it results in batch-to-batch variability and generates heterogeneous ADCs, which have been shown to have a narrow therapeutic window relative to homogeneous ADCs, therefore having major pharmacokinetic limitations^9^,^10^. Cysteine modification, following interchain disulfide reduction, results in the permanent loss of structural disulfide bonds, which may reduce the stability of the ADC *in vivo*^7^,^8^. It also generates heterogeneous mixtures when targeting typical drug-to-antibody ratios. Other approaches using cysteine-based site-directed mutagenesis and unnatural amino acids have also been described^10^,^11^, but similarly have limitations, for example, disulfide scrambling post-reduction and high cost combined with relatively low expression yields, respectively.

Recently, we have described methods for the insertion of small molecules into disulfide bonds in various proteins, engineered antibody single-chain variable fragments, a fragment antigen-binding (Fab) construct to yield site-selectively modified products and a full antibody^12^,^13^,^14^,^15^,^16^,^17^,^18^,^19^. This has gone some way to addressing the issues of site-selective antibody modification. There is also relevant work in this area by Godwin and coworkers, which highlights an alternative set of reagents for site-selective disulfide bridging of antibodies^20^. However, significant barriers still remain in this rapidly evolving field and there is demand for novel ways of constructing ADCs with stable and versatile linkers with retention of core antibody structure^1^,^2^,^3^,^4^,^5^,^6^.

In this manuscript, we describe a significant step towards a platform for next-generation ADCs by providing constructs that combine site-specific native antibody modification, exceptional versatility (*via* a ‘dual click’ approach), high stability and retention of antibody structure post-modification. The technology, at its core, is based on the insertion of pyridazinediones (PDs) bearing orthogonal ‘clickable’ handles into native disulfide bonds in antibody fragments and full antibodies, with a view to then carry out two orthogonal transformations to yield multifunctionalized adducts (Fig. 2). This enables the rapid assembly of dual-modified ADCs in a highly convergent manner. The work described herein could pave the way to novel antibody-based therapeutics.

## Results

### Antibody scaffold, drug and fluorophore selection

To evaluate this chemistry, a suitable antibody system and cytotoxic drug needed to be selected. Trastuzumab (Herceptin), a monoclonal immunoglobulin G1 (IgG1) that targets the internalizing HER2 receptor, has been used successfully in the treatment of HER2+ breast cancer and is the antibody component of a recently FDA-approved ADC therapy for the same indication, trastuzumab emtansine (Kadcyla)^21^,^22^. Anticancer drug doxorubicin (Dox) has been used as a cytotoxic model payload previously and has a relatively distinctive absorbance maximum at 495 nm to facilitate determination of drug-to-antibody ratios by ultraviolet–visible absorption^12^. As such, Herceptin and Dox were chosen as the antibody and cytotoxic platforms, respectively. To analyse the effectiveness of the ‘dual click’ approach on a full antibody scaffold, where accurate mass spectrometry analysis is limited, a second light absorbing moiety that absorbs at a distinct wavelength to Dox was needed to enable facile analysis by ultraviolet–visible spectrometry of the loading of each cargo. To this end, a photostable, water-soluble, cyanine-based fluorophore with a maximum absorbance at 646 nm (sulfo-Cy5) was selected.

### Choice of linker

**Figure 3:**
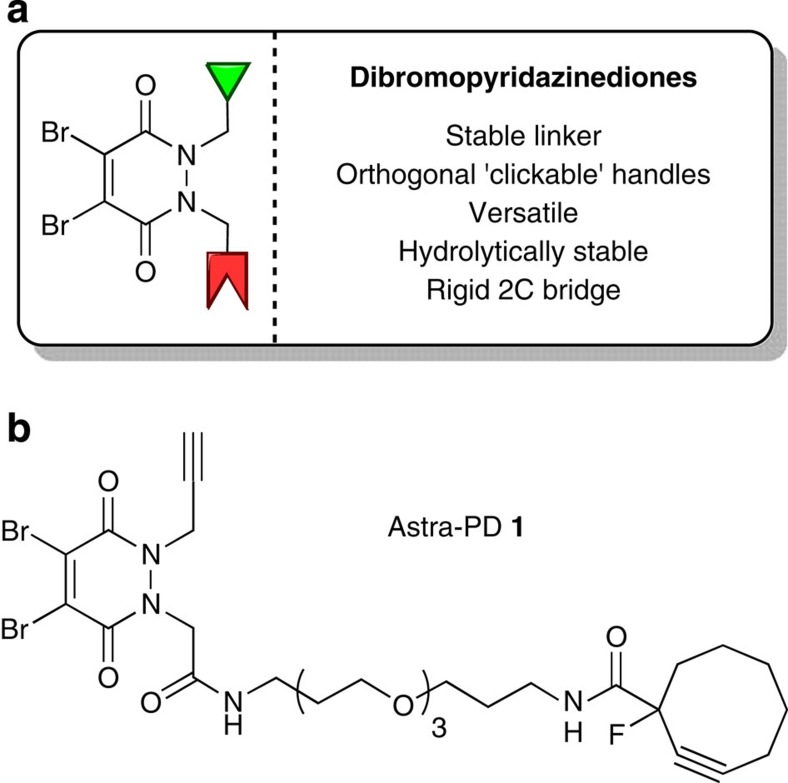
Properties of dibromopyridazinediones and structure of Astra-PD 1. (**a**) General properties of the pyridazinedione platform. (**b**) Chemical structure of Astra-PD **1** with an alkyne and a strained alkyne handle.

In order to deliver a widely applicable and versatile approach to antibody modification, it was rationalized that an exceptionally stable linker bearing multiple modalities that could be introduced *via* conjugation onto native antibodies was required. A suitable scaffold was dibromopyridazinedione (diBrPD) as it has previously been shown to be efficient at inserting into disulfide bonds and the resulting constructs to be exceptionally stable to hydrolysis, even at high temperatures (Fig. 3)^18^. Moreover, their structure is appealing as they are ideally set up for attaching various modalities *via* each nitrogen atom. As we wanted the platform to be versatile and widely applicable, orthogonal ‘clickable’ handles, one on each nitrogen atom, onto the PD motif were to be attached. To this end, Astra-PD **1**, an alkyne-strained alkyne-pyridazinedione was synthesized (Fig. 3, see Supplementary Information for details on the synthesis).

### Appraisal of dual click strategy on an antibody fragment

**Figure 4:**
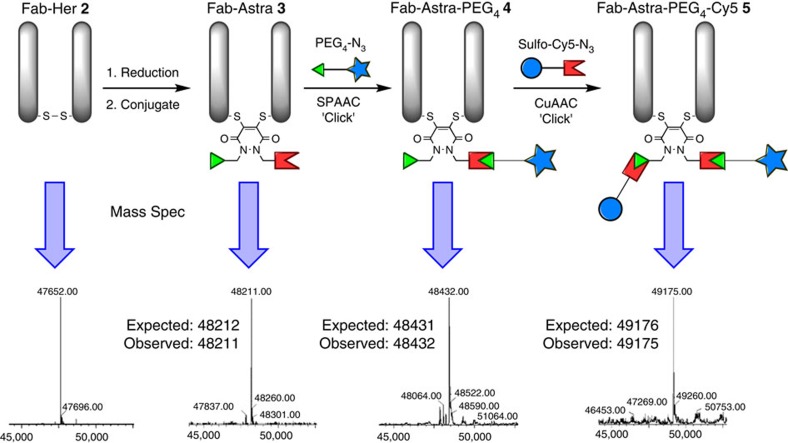
Sequential modifications from native Fab-Her 2 to afford Fab-Astra-PEG4-Cy5 5. Re-bridging of Fab-Her **2** and regioselective dual modification using SPAAC with PEG_4_-N_3_ followed by CuAAC with Sulfo-Cy5-N_3_ to yield Fab-Astra-PEG_4_-Cy5 **5** conjugate as a single product.

With PD-construct **1** in hand, the insertion of this molecule into a Fab fragment of Herceptin; Fab-Her **2**, by simple reduction of the single interchain disulfide bond, followed by functional disulfide re-bridging with the PD construct was carried out (Fig. 4), which afforded exclusive formation of a re-bridged Fab fragment with a PD molecule inserted into the disulfide bond, construct **3**, by mass spectrometry and SDS–PAGE. No further purification was required and a yield in excess of 95% was obtained (Supplementary Figs 11 and 12).

Moreover, the two orthogonal reactive handles could be utilized to introduce distinct functionalities selectively. For example, construct **3** was reacted with PEG_4_-N_3_ followed by reaction with sulfo-Cy5-N_3_, by applying strain-promoted azide–alkyne cycloaddition (SPAAC) and copper(I)-catalysed azide–alkyne cycloaddition (CuAAC) chemistry, respectively, to form species **5** (Fig. 4). These reactions were shown to be clean and high yielding with complete selectivity, as demonstrated by mass spectrometry and SDS–PAGE (Fig. 4 and Supplementary Figs 13 and 14).

**Figure 5:**
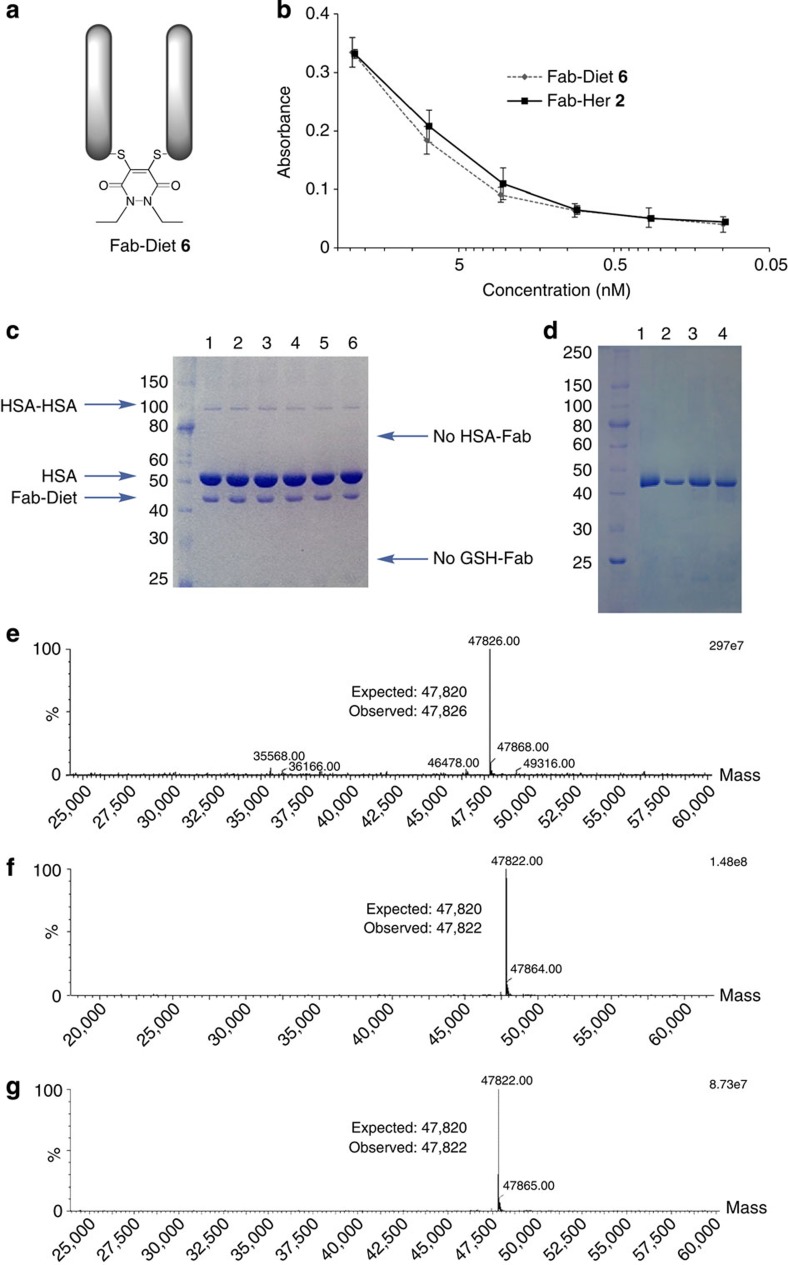
Activity and stability data for model construct Fab-Diet 6. (**a**) Structure of Fab-Diet **6**. (**b**) Binding activity data for native Fab-Her **2** and Fab-Diet **6** assessed by ELISA. (**c**) SDS–PAGE gel following incubation of Fab-Diet **6** in blood plasma mimicking conditions for 0, 1, 2, 3, 5 and 7 days (lanes 1–6, respectively). (**d**) SDS–PAGE gel of Fab-Diet **6**, Fab-Diet **6** after 8 months of storage at 4 °C in PBS, Fab-Diet **6** after 24 h at 37 °C in buffer pH 3.1 and Fab-Diet **6** after 24 h at 37 °C in buffer pH 9.0 (lanes 1–4, respectively). (**e**) Deconvoluted MS data of Fab-Diet **6** after 8 months of storage at 4 °C in PBS. (**f**) Deconvoluted MS data of Fab-Diet **6** after 24 h at 37 °C in buffer pH 3.1. (**g**) Deconvoluted MS data of Fab-Diet **6** after 24 h at 37 °C in buffer pH 9.0.

Furthermore, model diethyl-PD-modified Fab construct **6** (Fab-Diet) was shown to have: (i) comparable binding activity by enzyme-linked immunosorbent assay (ELISA) to Fab-Her **2**; (ii) excellent stability in blood plasma mimicking conditions for 7 days; (iii) complete stability after 8 months of storage at 4 °C in phosphate-buffered saline (PBS); and stability in (iv) low pH (3.1) and (v) high pH (9.0) conditions at 37 °C over a protracted period (the blood plasma mimicking conditions correspond to the simulated body fluid described by Jalota *et al.*^23^ with addition of human serum albumin (600 μM, 40 mg ml^−1^) and glutathione (20 μM); Fig. 5 and Supplementary Figs 15–20).

There are plentiful opportunities in multimodal imaging and theranostics, which can be made available by the dual labelling technology described by attachment of a fluorophore and a radiolabel or cytotoxic drug, respectively^24^,^25^. Furthermore, this technology allows for the construction of novel ADC fragment-based products. In terms of therapeutics, antibody fragments offer potential advantages over full antibodies (for example, fragments are smaller and offer greater tissue penetration, and many lack the antibody Fc region and can therefore be expressed in bacterial cell lines as there is no requirement for post-translational glycosylation)^3^,^26^,^27^,^28^. However, antibody fragments below the *ca.* 60–70 kDa size cut-off for glomerular filtration lack the stability afforded by the antibody Fc region and therefore often have insufficient lifetime in blood serum required to elicit a beneficial therapeutic effect without intensive and frequent therapy^28^,^29^,^30^,^31^,^32^,^33^. It is envisaged that the technology will allow antibody fragment therapeutics to be far more accessible by delivering long lifetime antibody fragment–drug conjugates. This could be achieved by the attachment of an antibody fragment to: (i) a suitable toxic drug; and (ii) a blood serum-stabilizing functionality (for example, PEG, albumin or albumin-binding functionality) to increase lifetime *in vivo*, using the dual labelling technology. Moreover, as the chemistry allows for the controlled stoichiometric addition of each moiety, it will allow for the construction of well-characterized products.

**Figure 6:**
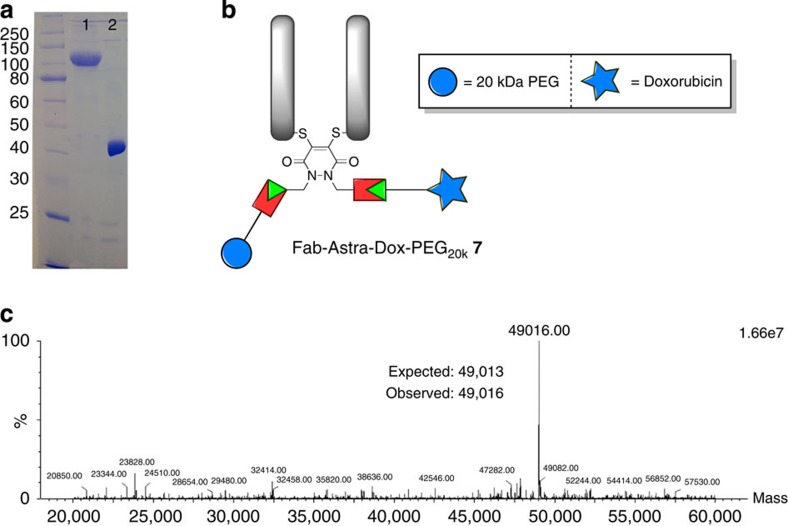
Construction of Fab-Astra-Dox-PEG_20k_ 7, a fragment-based ADC with an additional lifetime extension modality. (**a**) SDS–PAGE analysis for bioconjugates Fab-Astra-Dox-PEG_20k_ **7** and Fab-Astra-Dox (lanes 1 and 2, respectively). (**b**) Structure of bioconjugate Fab-Astra-Dox-PEG_20k_ **7**. (**c**) Deconvoluted MS data of Fab-Astra-Dox.

The construction of an antibody fragment functionalized with a life-extension technology and a cytotoxic drug was initiated by choosing Dox as the drug payload and a 20 kDa PEG chain as the lifetime extension modality (as its effects on half-life are well-characterized *in vivo* and it does not significantly compromise the penetration ability of a Fab fragment)^34^,^35^,^36^. As such, Fab-Astra **3** was treated with Dox-N_3_ (see Supplementary Information for details on the synthesis) and PEG_20k_-N_3_ in a sequential manner using SPAAC and CuAAC chemistry, respectively, to afford construct **7** by liquid chromatography–mass spectrometry (LC–MS) and SDS–PAGE analysis, without the need for gel-filtration chromatography (Fig. 6 and Supplementary Figs 21 and 22). The formation of this construct with almost complete retention of binding activity by ELISA on HER2 and no unspecific binding observed on EGFR (HER1; Fig. 6 and Supplementary Fig. 23) paves the way for the appraisal of the strategy on delivering antibody fragment-based therapeutics. The retention in binding affinity post-conjugation to a large construct is due to the installation of the PD-bridging moiety at a position that is distal from the binding site, which is another important feature of the chemistry. Moreover, the construction of a wide variety of similar lifetime extension/drug modalities should be facile and allow for rapid evaluation of various combinations of these modalities. The construction of Fab-Astra-Dox-PEG_20k_ **7** also further demonstrates the flexibility of the chemistry platform to allow for the attachment of large constructs such as a 20-kDa PEG moiety in a facile manner.

### Appraisal of dual click strategy on a full antibody

The use of Astra-PD **1** was then applied to the bridging of disulfide bonds in the full antibody system of trastuzumab (Herceptin), a clinically approved IgG1 full antibody comprising four disulfide bonds. Successful application of this method on this scaffold would allow for the construction of a site-specifically dual-functionalized full ADC with retention of core antibody structure.

**Figure 7:**
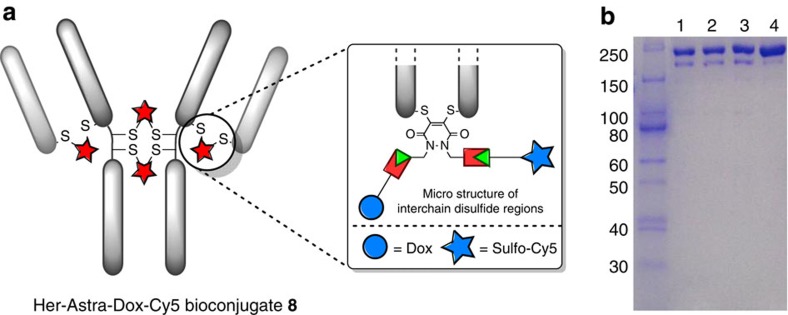
Construction of Her-Astra-Dox-Cy5 8, a dually modified ADC with both a cytotoxic drug and a fluorophore. (**a**) Structure of fully re-bridged and dually functionalized Herceptin. (**b**) SDS–PAGE analysis of Her-Astra, Her-Astra-Dox, Her-Astra-Dox-Cy5 **8** and Herceptin (lanes 1–4, respectively).

**Figure 8:**
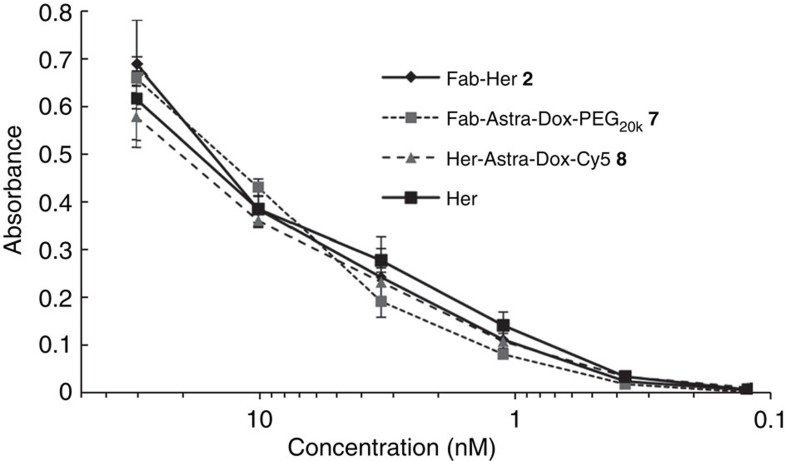
Binding activity of dually modified constructs by ELISA. Binding activity of Fab-Her **2**, Fab-Astra-Dox-PEG_20k_ **7**, Her-Astra-Dox-Cy5 **8** and unmodified full antibody Herceptin (Her).

Initially, Herceptin was modified with PD-construct **1** by reduction of the full antibody interchain disulfide bonds followed by functional re-bridging. Through optimization of temperature and concentration of reagents, a full antibody that was re-bridged in the correct orientation with a 4:1 ratio of PD-to-antibody by SDS–PAGE and ultraviolet–visible spectrometry was obtained (Fig. 7 and Supplementary Figs 24 and 25). This provides the first example of functionally re-bridging the disulfide bonds of a full antibody with almost complete retention of full antibody structure. Following this, the site-selectively modified full antibody was functionalized with a drug (Dox-N_3_) and a fluorophore (sulfo-Cy5-N_3_) using click chemistry. It was confirmed by ultraviolet–visible spectrometry that each species was attached with a loading of four, and by SDS–PAGE that there was retention of full antibody structure (Fig. 7 and Supplementary Figs 24 and 25). Construct **8** was also stable under a range of pH conditions and in blood plasma mimicking conditions (Supplementary Figs 26 and 27). These results provide proof of concept for the ability to attach multiple modalities in a controlled manner to a full antibody and a new approach for native antibody functionalization^37^. Moreover, retention of binding by ELISA on HER2, and no unspecific binding on EGFR (HER1) or evidence of aggregation over a protracted period could be observed (Fig. 8 and Supplementary Fig. 23).

### Internalization study

**Figure 9:**
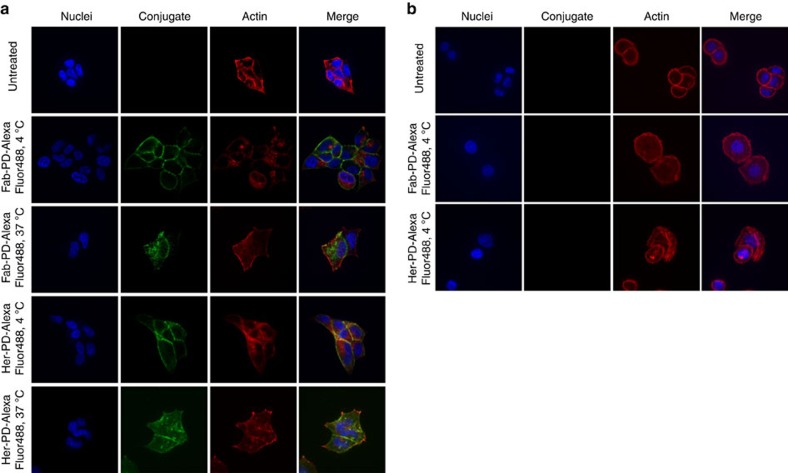
Internalization study of fragment-based and full length antibody-based bioconjugates. (**a**) BT-474 breast cancer cells were treated at 4 °C with Her-PD-AlexaFluor488 and Fab-PD-AlexaFluor488 (green); both constructs bound at this temperature and were found inside the cells once internalization was allowed by incubating at 37 °C for 1 h. (**b**) No signal from fluorophore-conjugated constructs Her-PD-AlexaFluor488 and Fab-PD-AlexaFluor488 was detected when allowed to bind to the MDA-MB-468 breast cancer cells.

The effect of the conjugation chemistry on the internalizing properties of Herceptin and Fab-Her **2** was appraised next. For this purpose, Herceptin and Fab-Her **2** were directly conjugated to AlexaFluor488 *via* a PD-linker to form Her-PD-AlexaFluor488 and Fab-PD-AlexaFluor488 (Supplementary Figs 28 and 29). These constructs were then used to treat breast cancer cell lines BT-474 (HER2-positive) and MDA-MB-468 (HER2-negative). Initially, incubation was performed at 4 °C, a temperature that allowed binding but not internalization; Her-PD-AlexaFluor488 and Fab-PD-AlexaFluor488 bound to BT-474 cells (Fig. 9a) but not to MDA-MB-468 cells (Fig. 9b) indicating the specificity of these constructs to HER2. Internalization of the bound antibodies occurred when the temperature was increased to 37 °C; incubation under these conditions for 1 h resulted in cytoplasmic localization of the labelled constructs in BT-474 cells (Fig. 9a), thus indicating that the chemistry employed does not interfere with the internalizing properties of Herceptin or Fab-Her **2**.

### Cytotoxicity assay

**Figure 10:**
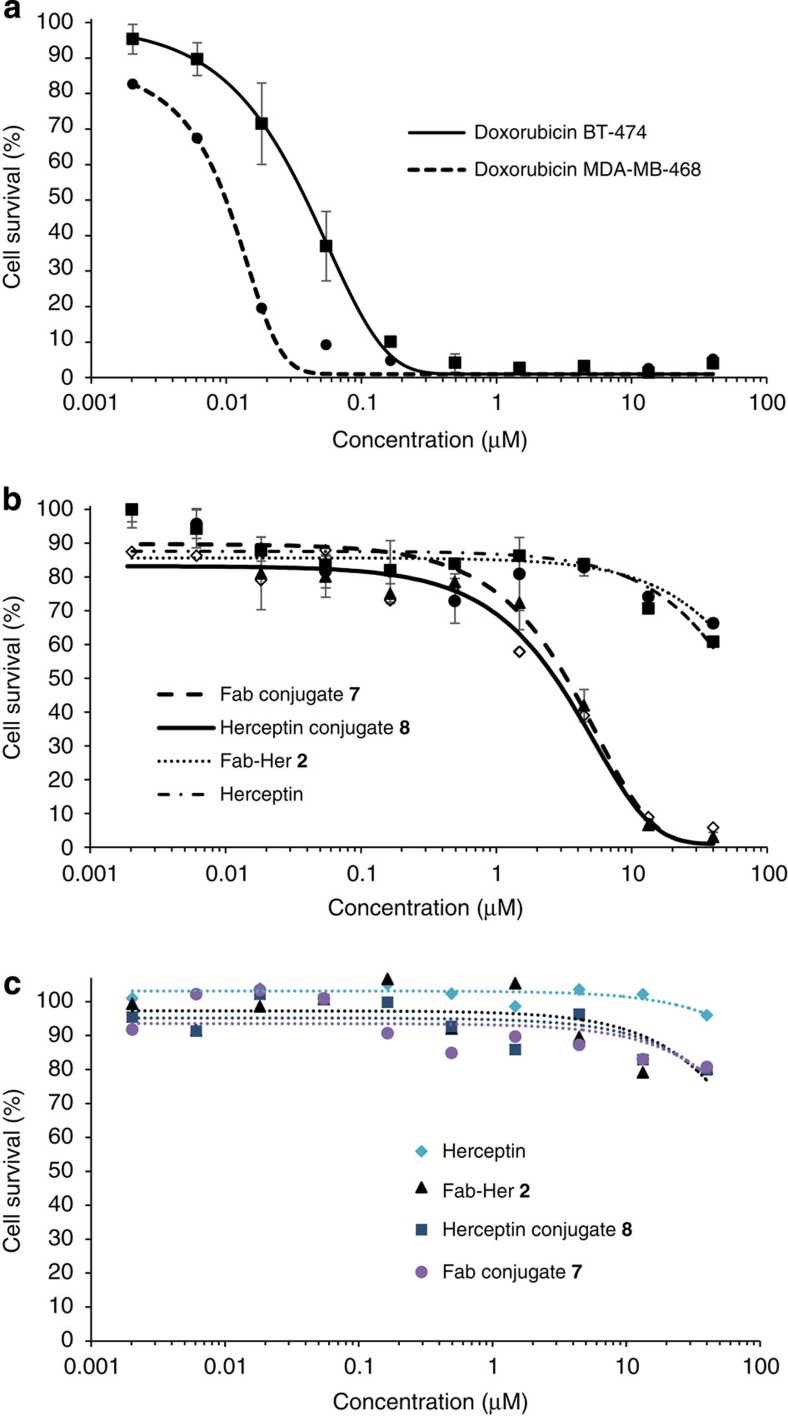
Inhibition of cell proliferation in cancer cell lines with different levels of HER2 expression. (**a**) BT-474 (HER2-positive) and MDA-MB-468 (HER2-negative): Dox alone; IC_50_=39 and 10 nM for BT-474 and MDA-MB-468, respectively. (**b**) BT-474: Fab-Astra-Dox-PEG_20k_ **7**, Her-Astra-Dox-Cy5 **8** (at similar concentration in Dox) in comparison with Herceptin and Fab-Her **2**; IC_50_=2.8 and 2.1 μM for conjugates **7** and **8,** respectively (**c**) MDA-MB-468: Fab-Astra-Dox-PEG_20k_ **7**, Her-Astra-Dox-Cy5 **8** (at similar concentration in Dox) in comparison with Herceptin and Fab-Her **2**.

Finally, the selectivity and cytotoxicity of constructs Fab-Astra-Dox-PEG_20k_ **7** and Her-Astra-Dox-Cy5 **8** in *in vitro* studies, using the same HER2-positive and -negative cell lines in the microscopy studies (that is, BT-474 and MDA-MB-468), were evaluated. Initially, the sensitivity of both cell lines to Dox to assess their suitability in a cytotoxicity assay was appraised. A comparable reduction in cell viability was observed for both cell lines at similar concentrations of the toxic payload (IC_50_=39 and 10 nM for BT-474 and MDA-MB-468, respectively) (Fig. 10a), thus paving the way for toxicity studies with Dox conjugates **7** and **8**. Notably, BT-474 cell viability was reduced significantly when incubated with the conjugates (IC_50_=2.8 and 2.1 μM for conjugates **7** and **8,** respectively), especially when compared with the controls of native Herceptin and Fab-Her **2** where the reduction in cell survival was minimal at high concentrations (Fig. 10b). In addition, highlighting the targeted delivery aspect of the method, no toxicity was observed in analogous studies with MDA-MB-468 cells incubated with either conjugate **7** or **8** (Fig. 10c). This is consistent with the observations by microscopy, that is, Herceptin and Fab-Her conjugates do not appear to bind to these cells, and hence Dox could not be internalized and thereby cause a toxic effect. These results highlight the selectivity of conjugates **7** and **8** over Dox alone, and indicate that the toxic drug in conjugates **7** and **8** is delivered by an HER2-dependent internalization mechanism.

Not only does this chemistry have the potential to make a significant contribution to the current ADC field, it is also flexible and has far reaching scope. Indeed, from a full antibody construct modified with Astra-PD **1**, it is wholly feasible that entirely new branches of strategies for ADC development will ensue. For example, the attachment of drugs that operate by an orthogonal mechanism of action, which is a concept that is gathering increasing momentum^38^,^39^,^40^,^41^.

## Discussion

In conclusion, by the introduction of PD molecules bearing orthogonal ‘clickable’ handles into the native disulfide bonds of antibodies, we could demonstrate: (i) the opportunity to synthesize multifunctionalized Fab fragments, which should be useful in a variety of applications (for example, imaging and theranostics); (ii) the potential of the technology to make a novel Fab-based ADC drug candidate; (iii) a new approach to a multifunctionalized, homogeneous full antibody to open the door to next-generation full antibody-based drug conjugate therapeutics; (iv) selective internalization into HER2-positive cells with conjugates of Herceptin and Fab-Her **2** that were directly conjugated to AlexaFluor488 *via* a PD-linker; and (v) selective cell killing over Dox alone for conjugates **7** and **8** on appropriate HER2 cell lines. Moreover, the ‘plug-and-play’ approach has broader applications in fields outside the scope of ADC technologies (for example, dual warhead antibiotics)^39^,^40^,^41^.

## Methods

### General experimental

All reagents were purchased from Sigma-Aldrich, Promega, Molecular Probes, AlfaAesar, Sino Biological, Invitrogen, UCLH or Lumiprobe and were used as received. Where described below, petrol refers to petroleum ether (40–60 °C). All reactions were monitored by thin-layer chromatography on pre-coated SIL G/UV254 silica gel plates (254 μm) purchased from VWR. Flash column chromatography was carried out with Kiesegel 60 M 0.04/0.063 mm (200–400 mesh) silica gel. ^1^H and ^13^C NMR spectra were recorded at ambient temperature on a Bruker Avance 500 instrument operating at a frequency of 500 MHz for ^1^H and 125 MHz for ^13^C and a Bruker Avance 600 instrument operating at a frequency of 600 MHz for ^1^H and 150 MHz for ^13^C in CDCl_3_ or CD_3_OD (as indicated below). The chemical shifts (*δ*) for ^1^H and ^13^C are quoted relative to residual signals of the solvent on the p.p.m. scale. ^1^H NMR peaks are reported as singlet (s), doublet (d), triplet (t), quartet (q), quint. (quintet), sext. (sextet), oct. (octet), m (multiplet), br (broad), dd (doublet of doublet), dt (doublet of triplets), ABq (AB quartet). Coupling constants (*J* values) are reported in hertz (Hz) and are H-H coupling constants unless otherwise stated. Signal multiplicities in ^13^C NMR were determined using the distortionless enhancement by phase transfer spectral editing technique. Infrared spectra were obtained on a Perkin Elmer Spectrum 100 FTIR Spectrometer operating in ATR mode with frequencies given in reciprocal centimetres (cm^−1^). Melting points were measured with a Gallenkamp apparatus and are uncorrected. Mass spectra of small molecule organic compounds were obtained on a VG70-SE mass spectrometer.

### Protein LC–MS

LC–MS was performed on protein samples using a Waters Acquity uPLC connected to Waters Acquity Single Quad Detector. Column: Hypersil Gold C4, 1.9 μm, 2.1 × 50 mm; wavelength: 254 nm; mobile phase: 95:5 water (0.1% formic acid):MeCN (0.1% formic acid); gradient over 4 min (to 5:95 water (0.1% formic acid):MeCN (0.1% formic acid); flow rate: 0.6 ml min^−1^; MS mode: ES+; scan range: *m*/*z*=250–2,000; scan time: 0.25 s. Data obtained in continuum mode. The electrospray source of the MS was operated with a capillary voltage of 3.5 kV and a cone voltage of 50 V. Nitrogen was used as the nebulizer and desolvation gas at a total flow of 600 l h^−1^. Ion series were generated by integration of the ultraviolet-absorbance (at 254 nm) chromatogram over the 1.4–2.0 min range. Total mass spectra for protein samples were reconstructed from the ion series using the MaxEnt 1 algorithm pre-installed on MassLynx software.

### Ultraviolet–visible spectroscopy

Ultraviolet–visible spectra were recorded on a Varian Cary 100 Bio UV-Visible spectrophotometer, operating at room temperature. Sample buffer was used as blank for baseline correction. Calculation of molecule over antibody ratio, *r*, follows the formula below with *ε*_280_=215,380 M^−1^ cm^−1^for Herceptin mAb, *ε*_280_=68,590 M^−1^ cm^−1^ for Herceptin Fab, *ε*_345_=9,100 M^−1^ cm^−1^ for Astra-PD, *ε*_495_=8,030 M^−1^ cm^−1^ for Dox, *ε*_646_=271,000 M^−1^ cm^−1^, and 0.724, 0.28 and 0.13 as a correction factor (CF) for Dox, Astra-PD and sulfo-Cy5, respectively, for the absorbance at 280 nm.


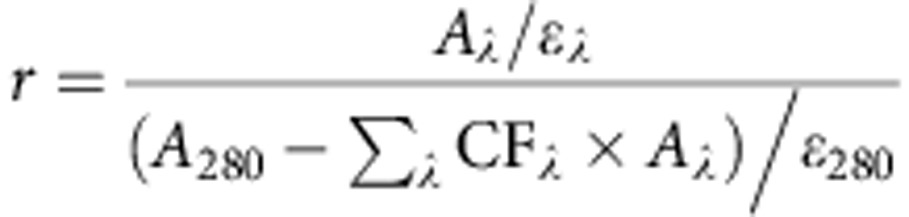


With *A*_*λ*_ the absorbance of a molecule at the wavelength *λ* in nm, and *ε*_*λ*_, the corresponding extinction coefficient.

### SDS–PAGE gels

Non-reducing glycine-SDS–PAGE at 12% acrylamide gels were performed following standard laboratory procedures. A 4% stacking gel was used and a broad-range molecular weight (MW) marker (10–250 kDa, BioLabs) was co-run to estimate protein weights. Samples (3–5 μl at ~10 μM in total mAb) were mixed with loading buffer (1–2 μl, composition for 6 × SDS: 1 g SDS, 3 ml glycerol, 6 ml 0.5 M Tris buffer pH 6.8, 2 mg R-250 dye) and heated at 75 °C for 3 min. The gel was run at constant current (30–35 mA) for 40 min in 1 × SDS running buffer. All gels were stained with Coomassie. Gel photographs were taken with a Wiko-Stairway device.

### Chemical biology

Conjugation of Astra-PD **1** onto Fab-Her **2**: To a solution of Fab-Her **2** (1 eq) in borate buffer (25 mM sodium borate, 25 mM NaCl, 0.5 mM EDTA, pH 8.0) was added TCEP (3 eq) and the reaction mixture was incubated at 37 °C for 90 min. After this time, was added a solution of Astra-PD **1** in DMF (5 eq) and the reaction mixture was incubated at 37 °C for 1 h. The excess reagents were then removed by repeated diafiltration into fresh buffer using VivaSpin sample concentrators (GE Healthcare, 10,000 molecular weight cut-off (MWCO)).

Conjugation of Astra-PD **1** onto Herceptin: To a solution of Herceptin (1 eq) in borate buffer (25 mM sodium borate, 25 mM NaCl, 0.5 mM EDTA, pH 8.0) was added TCEP (10 eq) and Astra-PD **1** (20 eq) in DMF and the reaction mixture was incubated at 4 °C for 6 h. The excess reagents were then removed by repeated diafiltration into fresh buffer using VivaSpin sample concentrators (GE Healthcare, 10,000 MWCO).

SPAAC click conditions: To a solution of an antibody or antibody fragment (1 eq) modified with Astra-PD **1** in PBS or borate buffer was added azide cargo (5 eq per re-bridged Astra-PD molecule) and the reaction mixture was incubated at 37 °C for 4 h. The excess reagents were then removed by repeated diafiltration into fresh buffer using VivaSpin sample concentrators (GE Healthcare, 10,000 MWCO).

CuAAC click conditions: To a solution of an antibody or antibody fragment (1 eq) modified with mono-functionalized-Astra-PD **1** in PBS was added tris(3-hydroxypropyltriazolylmethyl)amine (1.25 mM), CuSO_4_ (250 μM) followed by the addition of azide cargo (3 eq for Herceptin-Fab-Astra-PD and 10 eq for Herceptin-Astra-PD) and sodium ascorbate (final concentration 5 mM), and the reaction mixture was incubated at 37 °C for 16 h. The excess reagents were then removed by repeated diafiltration into fresh PBS with 2 mM EDTA (to remove residual copper ions) using VivaSpin sample concentrators (GE Healthcare, 10,000 MWCO).

### Cell lines

Breast cancer cell lines BT-474 and MDA-MB-468 were purchased from ATCC. BT-474 cells were maintained at 37 °C, 5% CO_2_ in Hybricare Medium (ATCC) complemented with 10% foetal calf serum (Labtech International, Ringmer, UK). MDA-MB-468 cells were maintained at 37 °C, 5% CO_2_ in Dulbecco’s modified Eagle’s medium complemented with 10% foetal calf serum and 2 mM L-glutamine (PAA Laboratories, UK).

### Internalization analysis by confocal microscopy

Cells on coverslips at 70% confluency were incubated with AlexaFluor488-conjugated constructs at 10 μg ml^−1^ for 1 h at 4 °C. Cells were extensively washed with PBS to remove unbound antibodies and incubated at 37 °C in growth media. Internalization was allowed for 1 h, followed by extensive washing and fixation with 4% formaldehyde for 10 min at 4 °C. Coverslips were then blocked with 5% goat serum in 0.3% Triton-X100 (Sigma). Actin was detected with phalloidin-568 (Invitrogen) and Hoechst trihydrochloride (Invitrogen) was used to stain cell nuclei. Coverslips were mounted on slides using ProLong Gold Antifade (Invitrogen) and examined using Perkin Elmer Spinning Disc Confocal microscope and Volocity Visualization software.

### Toxicity assays

Cells were seeded in 96-well plates at 10^4^ cells/well and allowed to attach for 24 h. Serial dilution of Herceptin, Fab-Her **2**, Dox, and conjugates **7** and **8** were added to the cells at concentrations ranging from 40 to 0 μM in complete growth medium. After 96 h, cell viability was measured using the CellTiter 96 Aqueous Non-radioactive cell proliferation assay (Promega) following manufacturer’s instructions. Cell viability was plotted as percentage of untreated cells.

### Ellman’s test

Herceptin and Fab-Her **2**, respectively; To a solution of trastuzumab (50 μl, 40 μM, 1 eq) in phosphate buffer (100 mM sodium phosphate, 1 mM EDTA, pH 8.0), was added TCEP (final concentration 400 μM, 10 eq) and the reaction mixture was incubated at 37 °C for 2 h. The excess reagents were then removed by repeated diafiltration into fresh buffer using VivaSpin sample concentrators (GE Healthcare, 10,000 MWCO). The concentration of protein was then adjusted to 40 μM. Following this, a solution of Ellman’s reagent (5,5′-dithio-*bis*-(2-nitrobenzoic acid)) in phosphate buffer (100 mM sodium phosphate, 1 mM EDTA, pH 8.0; final concentration 1.6 mM, 40 eq) was added, and the reaction mixture was incubated at 21 °C for 30 min. Analysis by ultraviolet–visible spectrometry after a tenfold dilution revealed an absorption at 412 nm of 0.439 that corresponds to 7.8 accessible sulfhydryl groups (*ε*_412_=14,150 M^−1^ cm^−1^). A similar experiment without TCEP reduction before incubation with Ellman’s reagent showed no absorbance at 412 nm.

To a solution of Fab-Her **2** (50 μl, 20 μM, 1 eq) in phosphate buffer (100 mM sodium phosphate, 1 mM EDTA, pH 8.0), was added TCEP (final concentration 100 μM, 5 eq) and the reaction mixture was incubated at 37 °C for 2 h. The excess reagents were then removed by repeated diafiltration into fresh buffer using VivaSpin sample concentrators (GE Healthcare, 10,000 MWCO). The concentration of protein was then adjusted to 20 μM. Following this, a solution of Ellman’s reagent (5,5′-dithio-*bis*-(2-nitrobenzoic acid)) in phosphate buffer (100 mM sodium phosphate, 1 mM EDTA, pH 8.0; final concentration 200 μM, 10 eq) was added, and the reaction mixture was incubated at 21 °C for 30 min. Analysis by ultraviolet–visible spectrometry revealed an absorption at 412 nm of 0.557 that corresponds to two accessible sulfhydryl groups (*ε*_412_=14,150 M^−1^ cm^−1^). A similar experiment without TCEP reduction before incubation with Ellman’s reagent showed no absorbance at 412 nm.

## Authors contributions

V.C. conceived and designed the project; V.C., A.M., M.E.B.S. and S.C. conceived and designed the chemistry and chemical biology experiments; A.M., V.C., E.M. and K.C. conceived and designed the biology experiments; A.M. and V.C. performed the chemistry and chemical biology experiments; A.M. and E.M. performed the biology experiments; V.C. and A.M. analysed the data; V.C., A.M. and S.C. co-wrote the paper.

## Additional information

**How to cite this article:** Maruani, A. *et al.* A plug-and-play approach to antibody-based therapeutics *via* a chemoselective dual click strategy. *Nat. Commun.* 6:6645 doi: 10.1038/ncomms7645 (2015).

## Supplementary Material

Supplementary Information Supplementary Figures 1-29 and Supplementary References

## Figures and Tables

**Figure 1:**
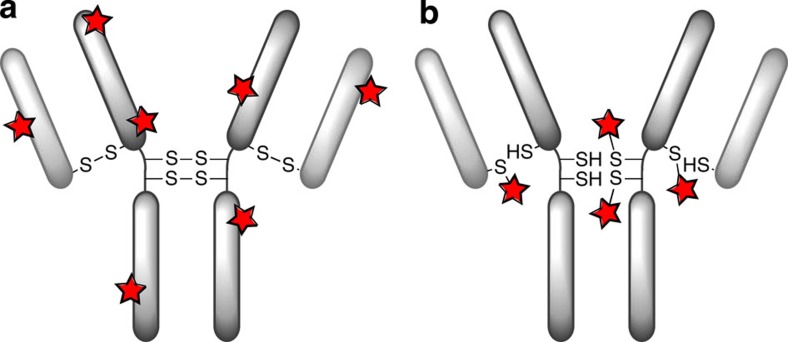
Approaches used for the generation of FDA-approved ADCs. (**a**) Lysine modification leading to a heterogeneous product mixture, undefined physical and pharmacokinetic properties, and wide distribution of drug-to-antibody ratio. (**b**) Modification of reduced disulfide bonds leading to a heterogeneous product mixture, loss of structural disulfide bonds and a suboptimal therapeutic index.

**Figure 2:**
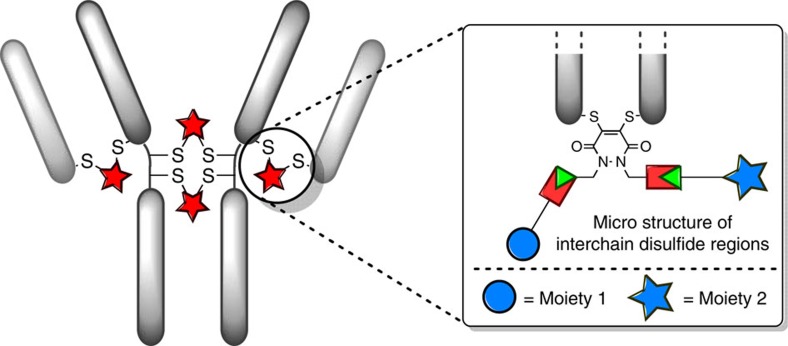
Functional disulfide re-bridging followed by a dual click approach. Disulfide re-bridging by a pyridazinedione construct yields a site-selectively modified antibody with dual modalities.
