# Supplementary material for: A plug-and-play approach to antibody-based therapeutics via a chemoselective dual click strategy
Source: Nat Commun. 2015 Mar 31;6:6645. doi: 10.1038/ncomms7645 (PMC4389247; doi:10.1038/ncomms7645)
Supplement: Supplementary Information — Supplementary Figures 1-29 and Supplementary References [file ncomms7645-s1.pdf]

Synthesis of compounds

Di-*tert*-butyl 1-(prop-2-yn-1-yl)hydrazine-1,2-dicarboxylate[1]

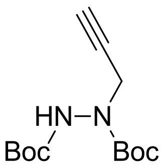

To a solution of di-*tert*-butyl hydrazine-1,2-dicarboxylate (300 mg, 1.29 mmol) in a mixture of toluene (2 mL) and 5% aq. NaOH (2 mL) was added tetra-*n*-butylammonium bromide (13 mg, 0.03 mmol) and propargyl bromide (461 mg, 3.87 mmol). The reaction mixture was stirred at 21 °C for 16 h. After this time, H_2O (20 mL) was added and the mixture was extracted with ethyl acetate (3 × 15 mL). The combined organic layers were washed with brine (15 mL), dried ($MgSO_4$), and concentrated *in vacuo*. Purification by flash column chromatography (20% EtOAc/petrol) yielded di-*tert*-butyl 1-(prop-2-yn-1-yl)hydrazine-1,2-dicarboxylate (435 mg, 1.61 mmol, 85%) as a white solid: m.p. 101-103 ºC (*lit. m.p.* 103.1–103.4 °C)[1]; ^{1}H NMR (500 MHz, $CDCl_3$) δ 6.47 (br s, 0.78H), 6.18 (br s, 0.22H,), 4.27 (s, 2H), 2.24 (t, J = 2.4 Hz, 1H), 1.48 (s, 18H); ^{13}C NMR (125 MHz, $CDCl_3$) δ 154.7 (C), 82.2 (C), 81.7 (C), 78.8 (C), 72.1 (CH), 39.7 (CH_2), 28.3 (CH_3), 28.2 (CH_3); IR (solid) 3310, 2112, 1703 cm^{-1}.

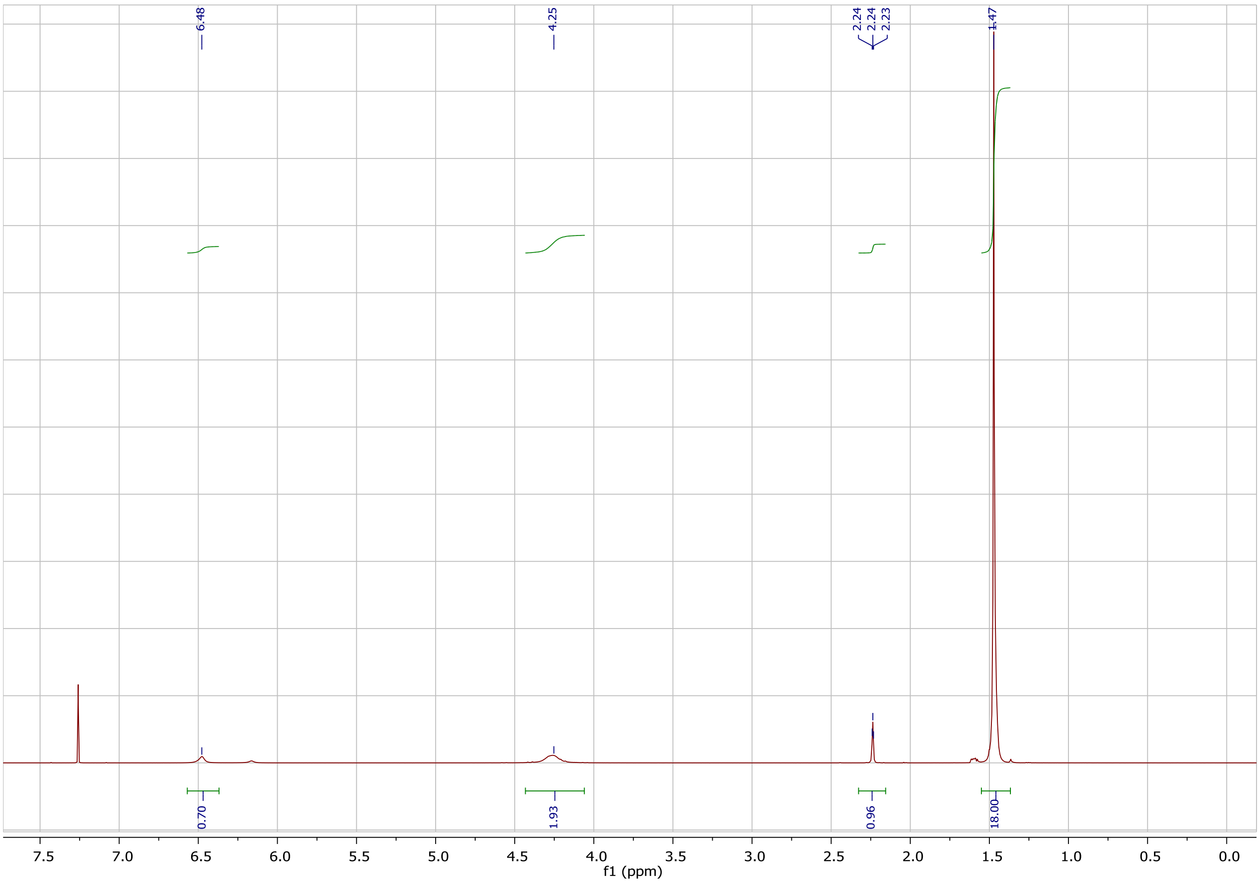

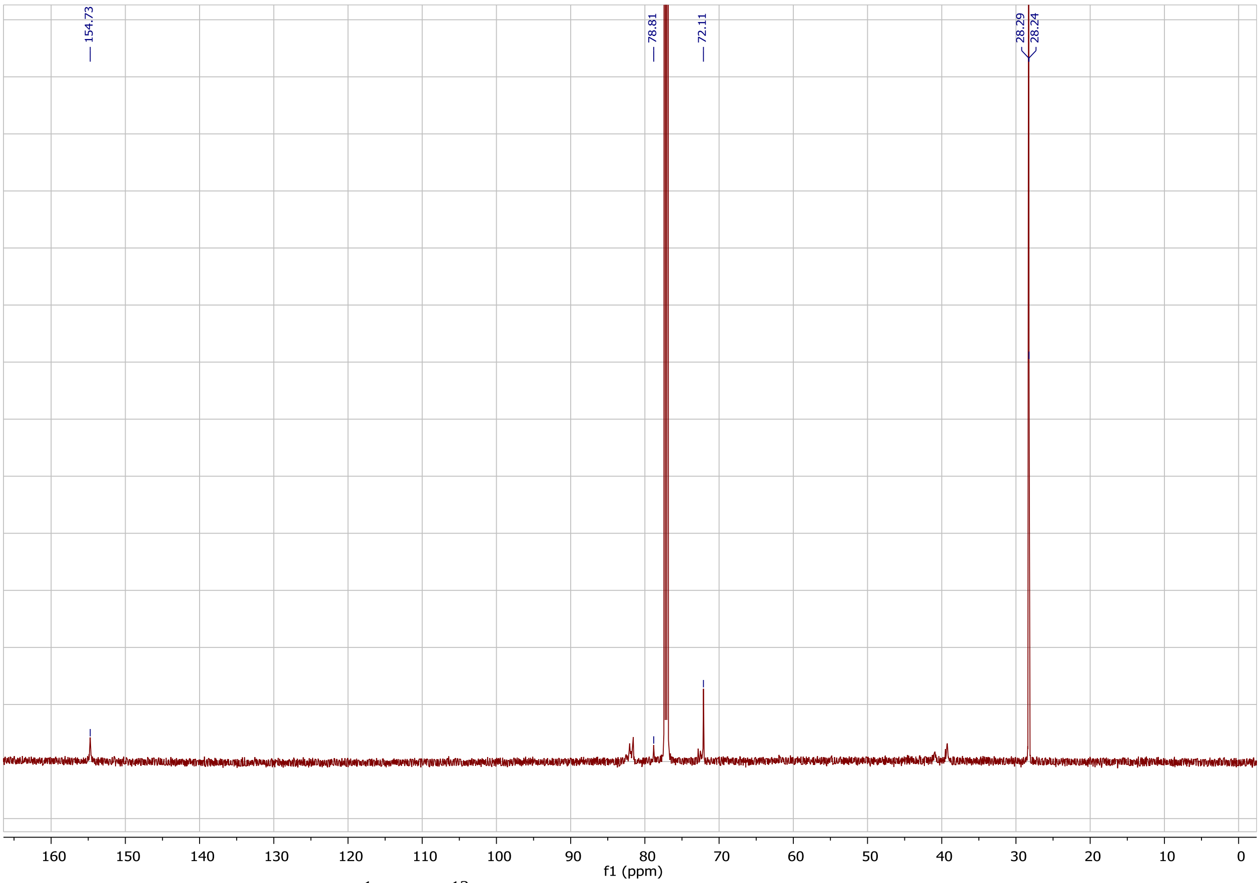

Supplementary Figure 1 ^{1}H and ^{13}C NMR data for di-*tert*-butyl 1-(prop-2-yn-1-yl)hydrazine-1,2-dicarboxylate.

Di-*tert*-butyl 1-(2-(*tert*-butoxy)-2-oxoethyl)-2-(prop-2-yn-1-yl)hydrazine-1,2-dicarboxylate

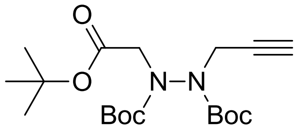

To a solution of di-*tert*-butyl 1-(prop-2-yn-1-yl)hydrazine-1,2-dicarboxylate (500 mg, 1.85 mmol) in DMF (15 mL) was added caesium carbonate (723 mg, 2.22 mmol) and *tert*-butyl bromoacetate (542 mg, 2.78 mmol) and the reaction mixture stirred at 21 °C for 16 h. After this time, the reaction mixture was diluted with H_2O (50 mL), extracted with EtOAc (4 × 50 mL), the combined organic layers washed with sat. aq. LiCl (2 × 30 mL), dried ($MgSO_4$), and concentrated *in vacuo* using toluene as an azeotrope. Purification by flash column chromatography (10% EtOAc/petrol) yielded di-*tert*-butyl 1-(2-(*tert*-butoxy)-2-oxoethyl)-2-(prop-2-yn-1-yl)hydrazine-1,2-dicarboxylate (713 mg, 1.85 mmol, >99%) as a colourless oil: ^{1}H NMR (600 MHz, $CDCl_3$) δ 4.58–4.35 (m, 2H), 4.16–4.01 (m, 1H), 3.67–3.64 (m, 1H), 2.18 (t, *J* = 2.4 Hz, 1H), 1.53–1.41 (m, 27H); ^{13}C NMR (150 MHz, $CDCl_3$) (major rotamer) δ 168.1 (C), 153.9 (C), 83.0 (C), 81.8 (C), 79.4 (C), 71.9 (CH), 53.5 (CH_2), 40.1 (CH_2), 28.3 (CH_3), 28.3 (CH_3), 28.1 (CH_3); IR (thin film) 3265, 2110, 1714 cm^{-1}; LRMS (CI) 385 (55, $[M+H]^+$), 329 (65), 273 (100), 217 (65); HRMS (CI) calcd for $C_{19}H_{33}O_6N_2$ $[M+H]^+$ 385.2333, observed 385.2319.

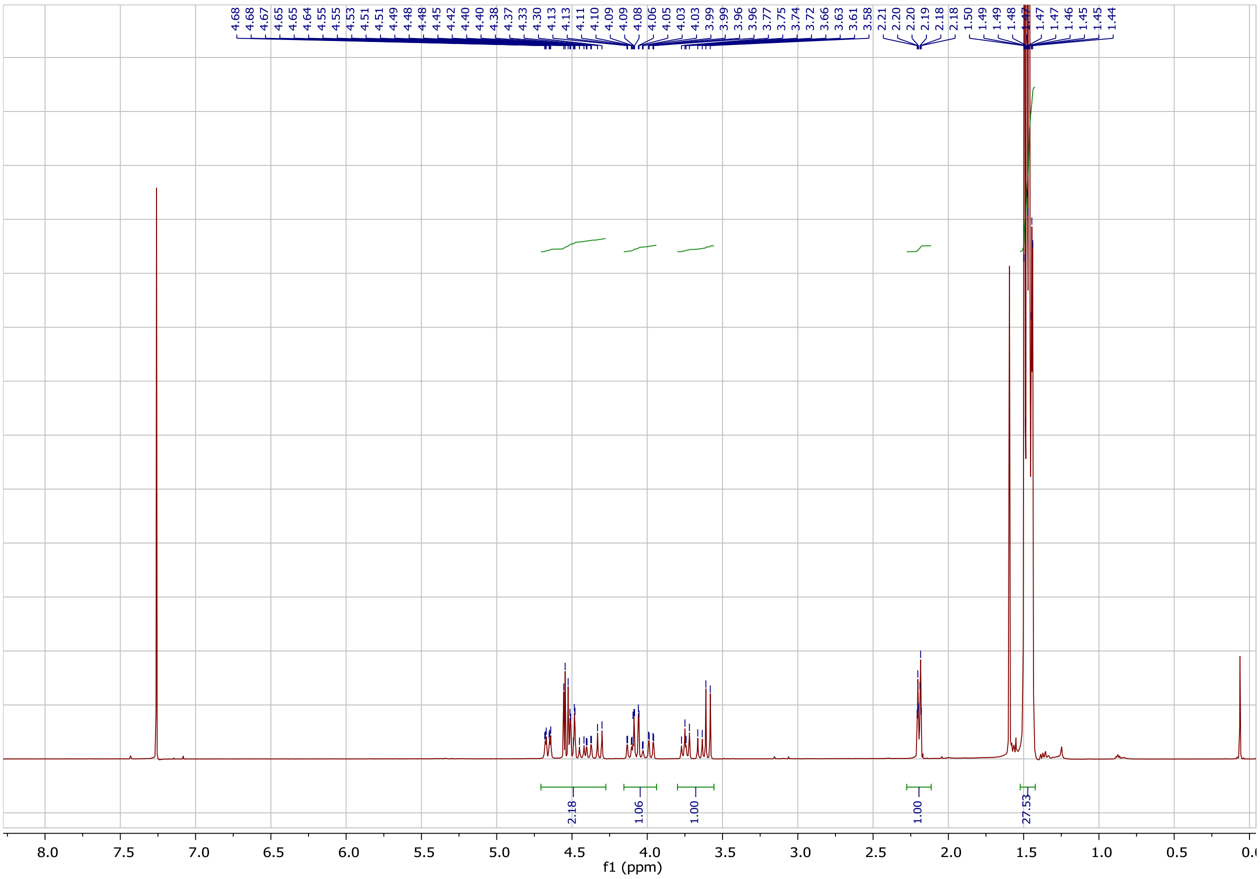

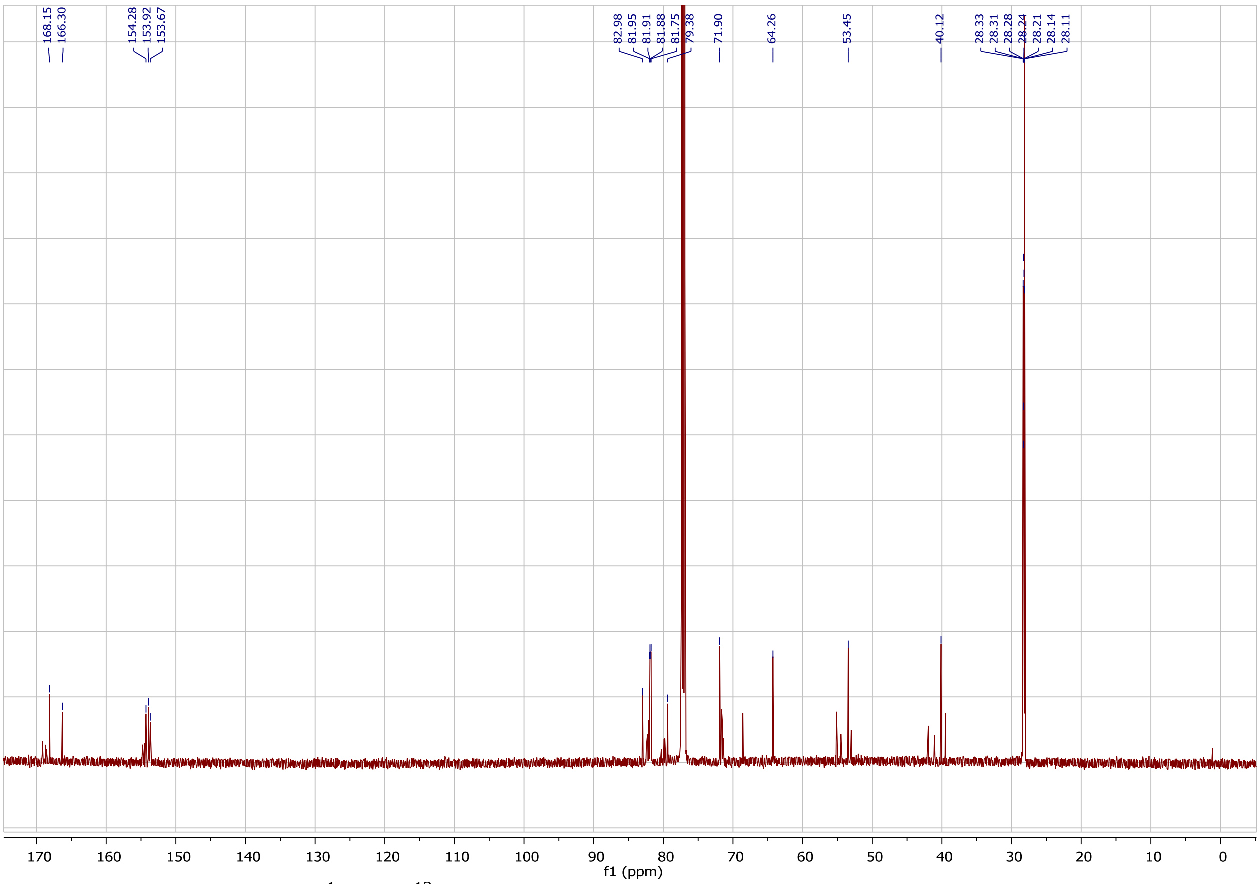

Supplementary Figure 2 ^{1}H and ^{13}C NMR data for di-*tert*-butyl 1-(2-(*tert*-butoxy)-2-oxoethyl)-2-(prop-2-yn-1-yl)hydrazine-1,2-dicarboxylate.

2-(4,5-Dibromo-3,6-dioxo-2-(prop-2-yn-1-yl)-2,3-dihydropyridazin-1(6*H*)-yl)acetic acid

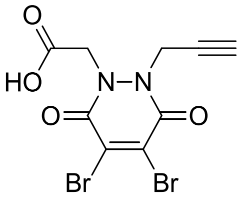

To a solution of di-*tert*-butyl 1-(2-(*tert*-butoxy)-2-oxoethyl)-2-(prop-2-yn-1-yl)hydrazine-1,2-dicarboxylate (500 mg, 1.30 mmol) in CH_2Cl_2 (10 mL) was added TFA (10 mL) and the reaction mixture stirred at 21 °C for 2 h. After this time, all volatile material was removed *in vacuo* using toluene as an azeotrope. The crude residue was added to a solution of 2,3-dibromomaleic anhydride[2] (397 mg, 1.55 mmol) in glacial AcOH (40 mL), and the reaction mixture heated at 130 °C for 16 h. Then the reaction mixture was concentrated *in vacuo*, and purification by flash column chromatography (1% AcOH/3% MeOH/DCM) yielded 2-(4,5-dibromo-3,6-dioxo-2-(prop-2-yn-1-yl)-2,3-dihydropyridazin-1(6*H*)-yl)acetic acid (243 mg, 0.66 mmol, 51%) as a white solid: m.p. 108–110 °C; ^{1}H NMR (600 MHz, MeOD-d_4) δ 4.95 (s, 2H), 4.91 (s, 2H), 2.98 (s, 1H); ^{13}C NMR (150 MHz, MeOD-d_4) δ 170.1 (C), 155.6 (C), 154.3 (C), 137.1 (C), 136.8 (C), 77.2 (C), 76.2 (CH), 50.3 (CH_2), 38.7 (CH_2); IR (solid) 3444, 3287, 2109, 1729, 1631 cm^{-1}; LRMS (CI) 369 (50, $[M^{81}Br^{81}Br+H]^+$), 367 (100, $[M^{81}Br^{79}Br+H]^+$), 365 (50, $[M^{79}Br^{79}Br+H]^+$); HRMS (CI) calcd for $C_9H_7N_2O_4{}^{79}Br_2$ $[M^{79}Br^{79}Br+H]^+$ 364.8767, observed 364.8762.

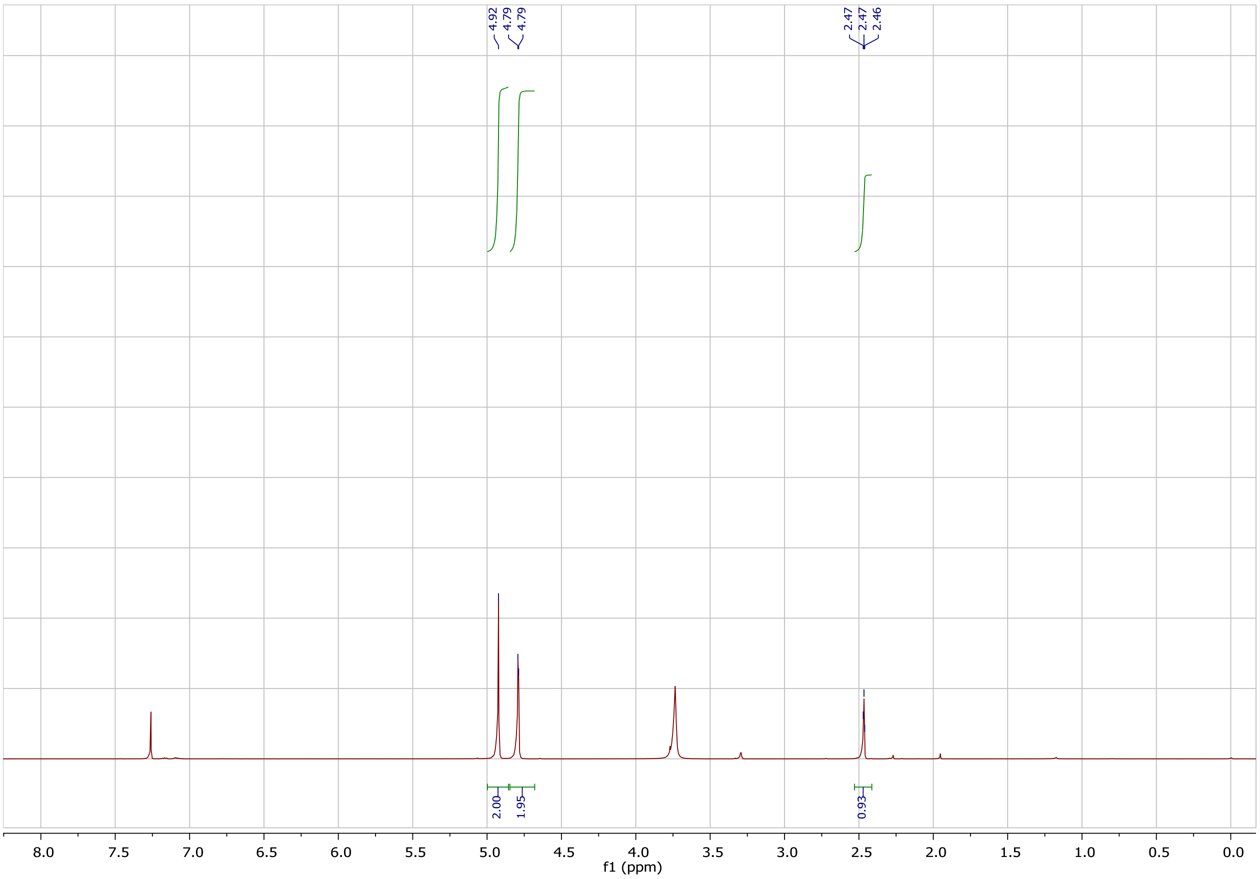

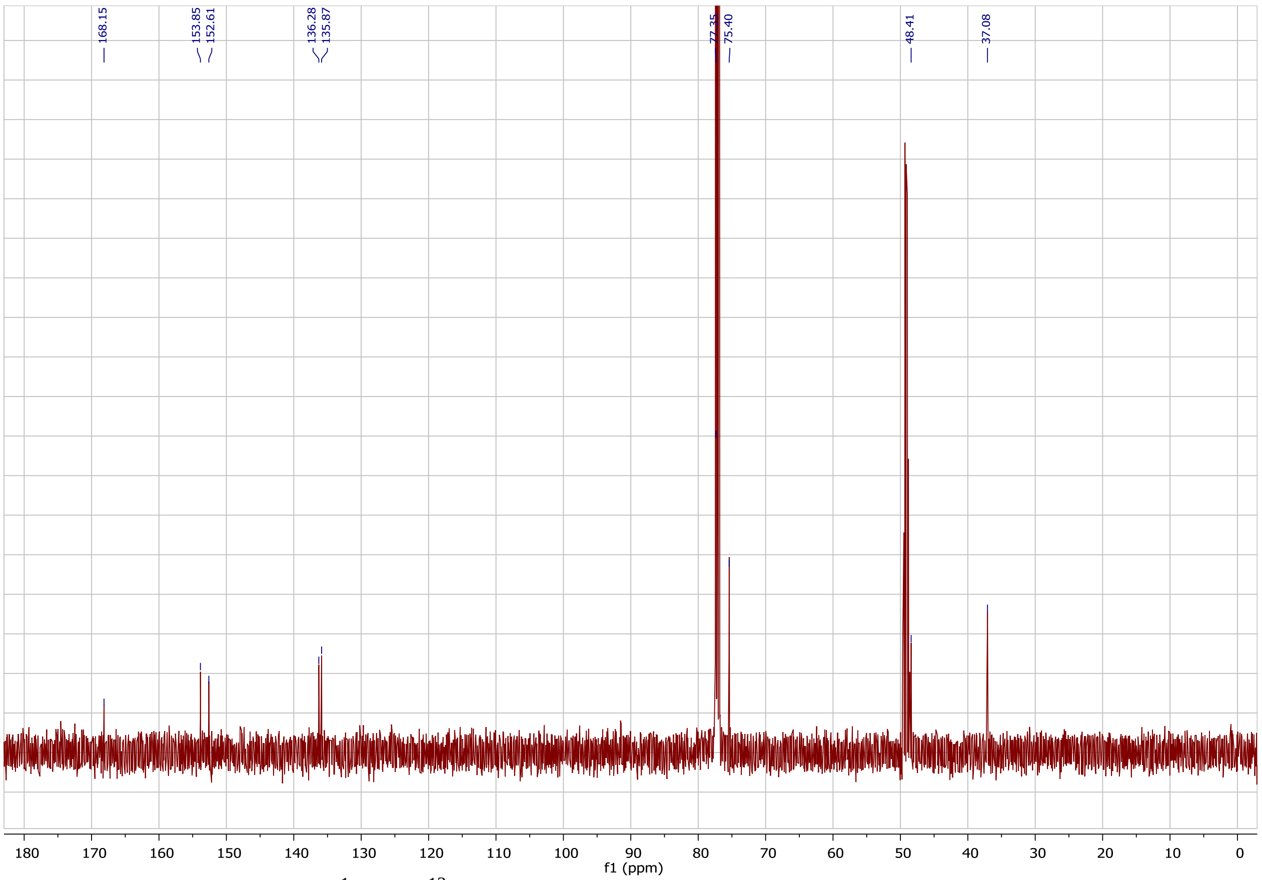

Supplementary Figure 3 ^{1}H and ^{13}C NMR data for 2-(4,5-dibromo-3,6-dioxo-2-(prop-2-yn-1-yl)-2,3-dihydropyridazin-1(6*H*)-yl)acetic acid.

(*Z*)-Methyl 2-hydroxycyclooct-1-enecarboxylate[3]

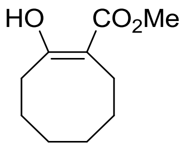

To a solution of dimethyl carbonate (1.4 g, 16 mmol) in toluene (20 mL) was added sodium hydride (0.88 g, 24 mmol). The resulting mixture was heated to 90 °C and a solution of cyclooctanone (1.0 g, 7.9 mmol) in toluene (5 mL) was added dropwise over 30 min. The resultant mixture was stirred at reflux for 4 h. The reaction mixture was cooled down to 0 °C and AcOH (2 mL) was added. The resulting precipitate was then dissolved by addition of 2 M HCl (6 mL). The mixture was extracted with toluene (3 × 30 mL) the combined organic layers washed with sat. aq. $NaHCO_3$ (20 mL), H_2O (20 mL), dried ($MgSO_4$), and concentrated *in vacuo*. Purification by flash column chromatography (10% Et_2O/petrol) yielded methyl 2-oxocyclooctanecarboxylate (1.4 g, 7.9 mmol, >99 %) as a clear liquid: ^{1}H NMR (500 MHz, $CDCl_3$) (major tautomer) δ 12.51 (s, 1H), 3.75 (s, 3H), 2.42–2.32 (m, 4H), 1.77–1.66 (m, 2H), 1.56–1.42 (m, 6H); ^{13}C NMR (125 MHz, $CDCl_3$) (major tautomer) δ 176.3 (C), 173.5 (C), 99.2 (C), 51.5 (CH_3), 32.4 (CH_2), 30.0 (CH_2), 28.8 (CH_2), 26.6 (CH_2), 26.2 (CH_2), 24.0 (CH_2); IR (thin film) 2960, 2940, 2860, 1740, 1700 cm^{-1}.

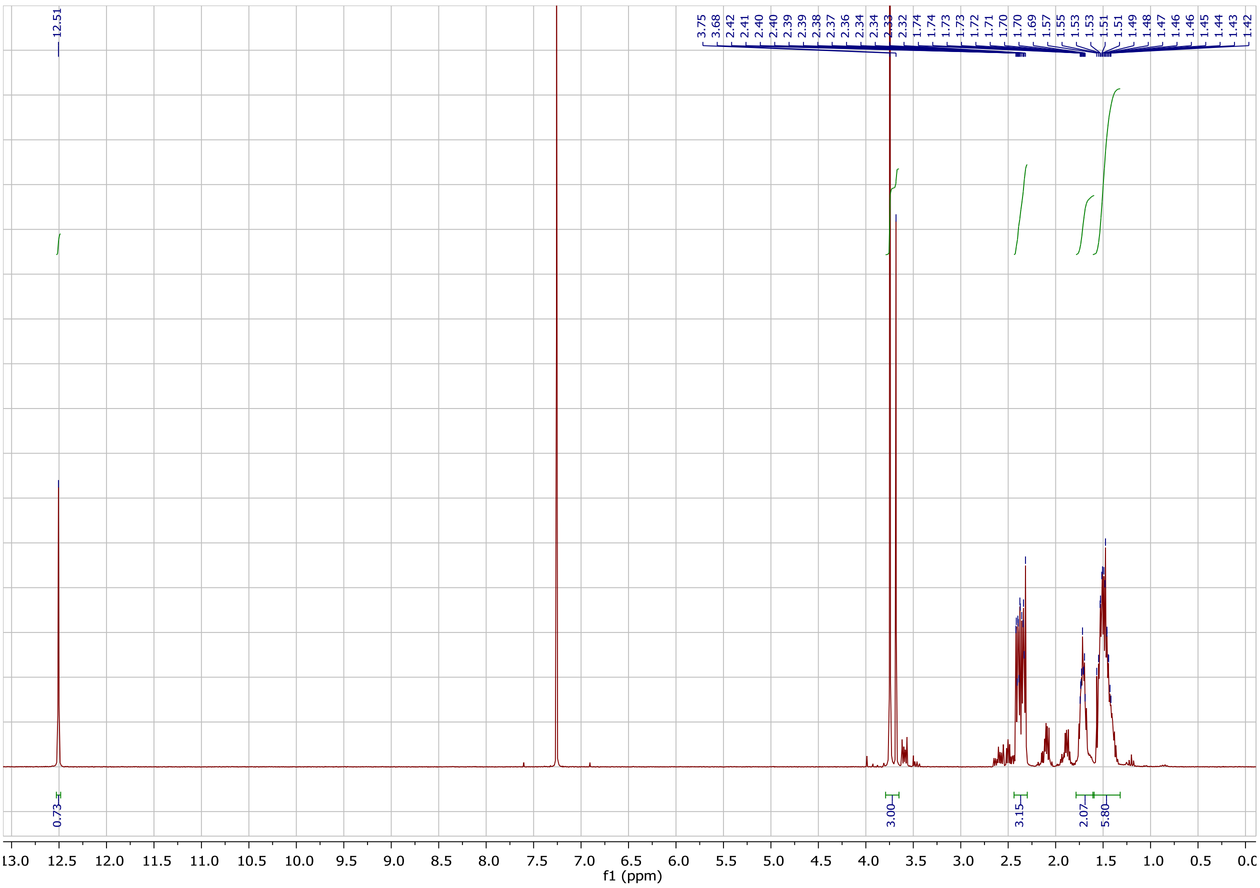

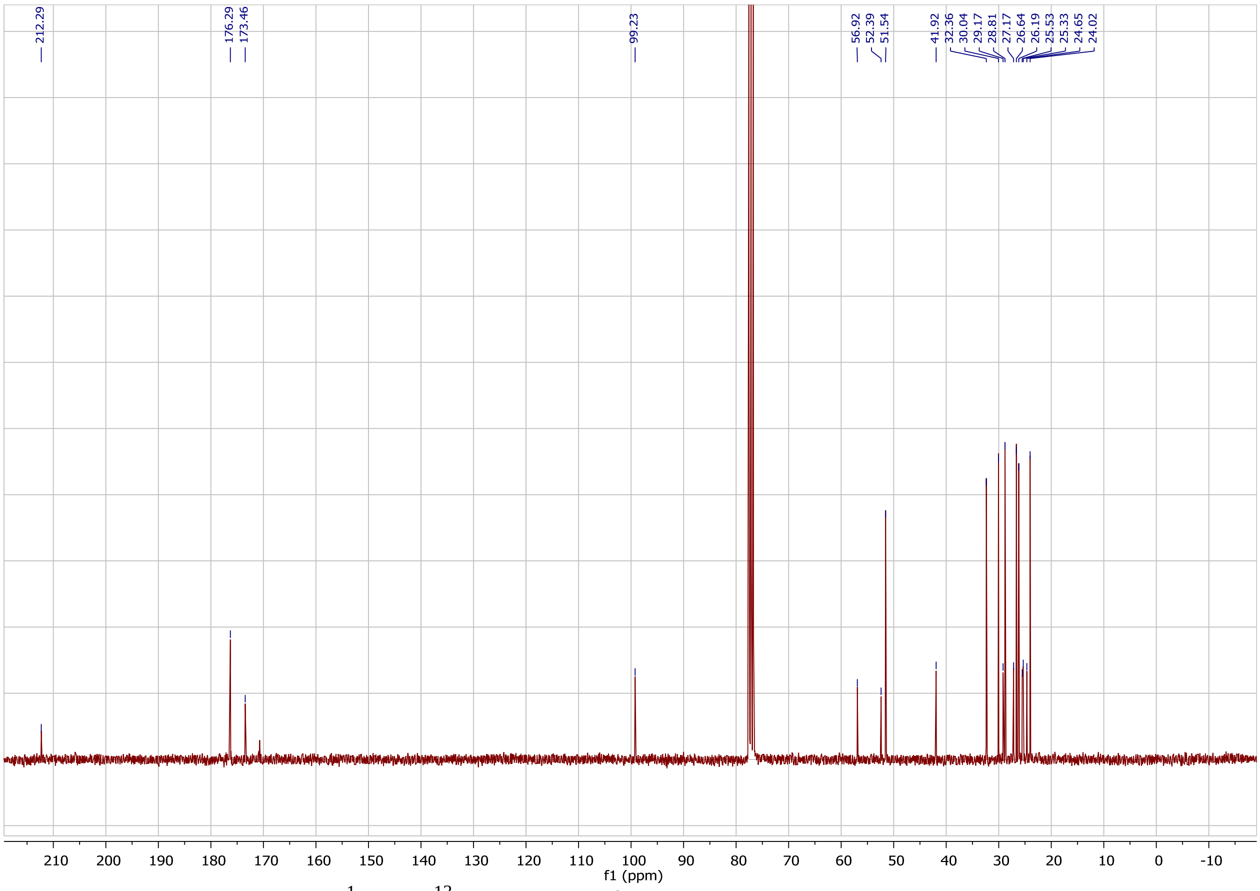

Supplementary Figure 4 ^{1}H and ^{13}C NMR data for (*Z*)-methyl 2-hydroxycyclooct-1-enecarboxylate.

Methyl 1-fluoro-2-oxocyclooctanecarboxylate[4]

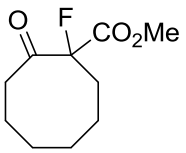

To a solution of (*Z*)-methyl 2-hydroxycyclooct-1-enecarboxylate (4.5 g, 25 mmol) in dry MeCN (80 mL) was added Selectfluor™ (10.4 g, 29 mmol) at 0 °C. The resulting mixture was stirred at 55 °C for 16 h. The reaction mixture was poured into water (200 mL), extracted with EtOAc (3 × 200 mL), washed with brine (3 × 100 mL), dried ($MgSO_4$), and concentrated *in vacuo*. Purification by flash column chromatography. Purification by flash column chromatography (10% Et_2O/petrol) yielded methyl 1-fluoro-2-oxocyclooctanecarboxylate (4.2 g, 21 mmol, 86%) as a white solid: ^{1}H NMR (500 MHz, $CDCl_3$) δ 3.75 (s, 3H), 2.69–2.46 (m, 3H), 2.22–2.16 (m, 1H), 1.84–1.79 (m, 2H), 1.69–1.56 (m, 3H), 1.47–1.33 (m, 3H); ^{13}C NMR (125 MHz, $CDCl_3$) δ 208.5 (d, $^{2}J_{C\text{-}F}$ = 22.1 Hz, C), 167.4 (d, $^{2}J_{C\text{-}F}$ = 24.9 Hz, C), 99.0 (d, $J_{C\text{-}F}$ = 200.6 Hz, C), 53.0 (CH_3), 38.7 (CH_2), 33.3 (d, $^{2}J_{C\text{-}F}$ = 22.1 Hz, CH_2), 27.3 (CH_2), 26.4 (CH_2), 24.3 (CH_2), 21.1 (CH_2); IR (thin film) 2924, 2853, 1751, 1713 cm^{-1}.

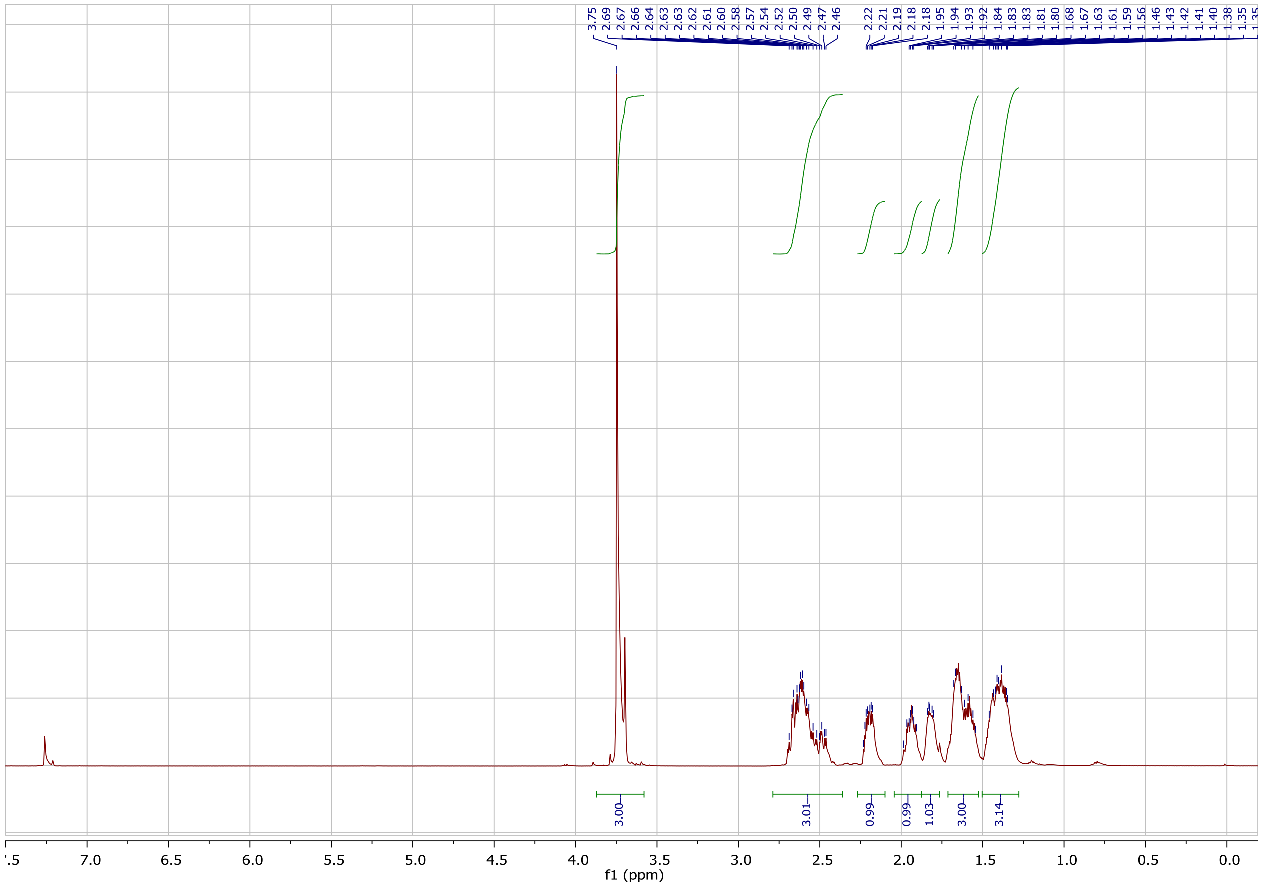

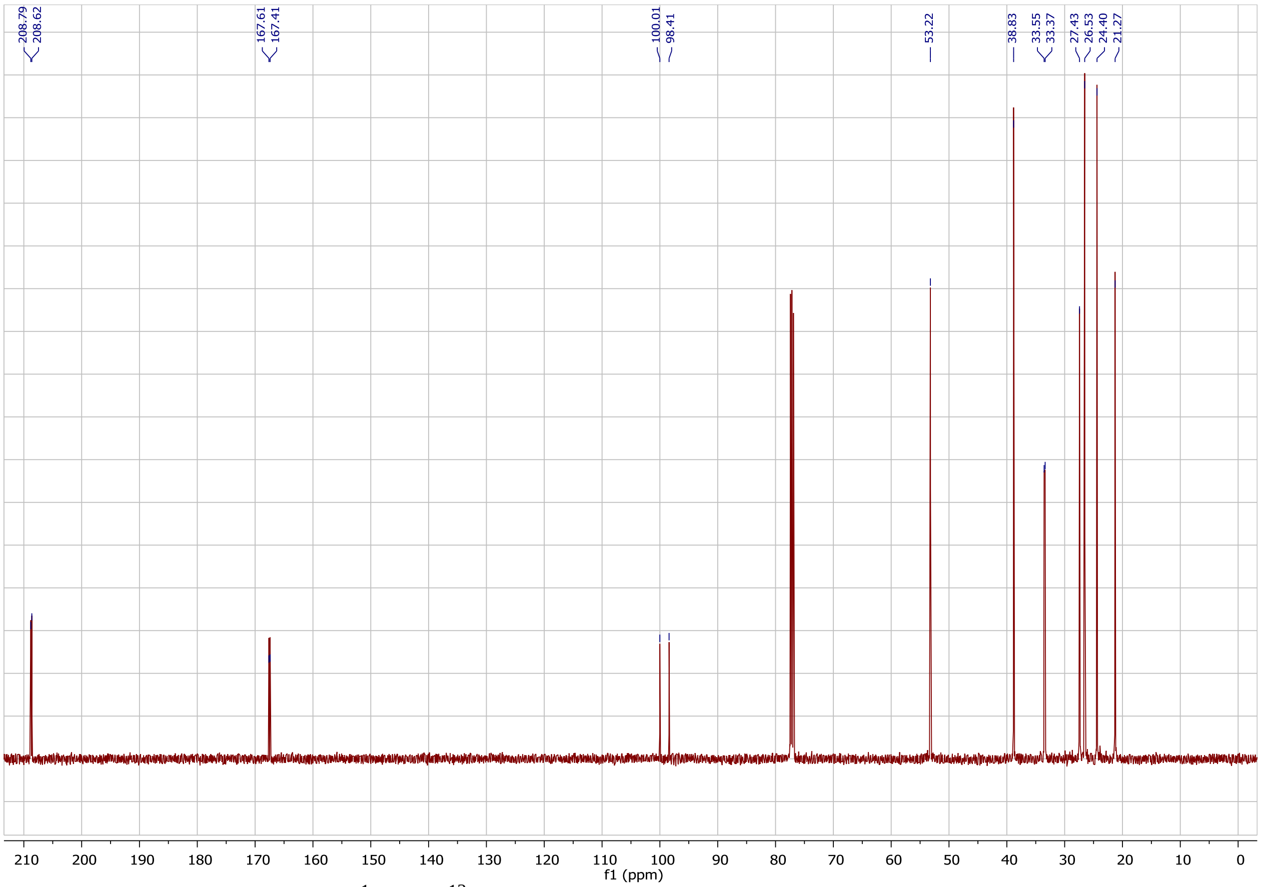

Supplementary Figure 5 ^{1}H and ^{13}C NMR data for methyl 1-fluoro-2-oxocyclooctanecarboxylate.

Methyl 1-fluorocyclooct-2-ynecarboxylate[4]

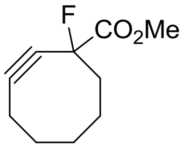

To solution of methyl 1-fluoro-2-oxocyclooctanecarboxylate (2.0 g, 9.9 mmol) in dry THF (200 mL) stirred at −78 °C was added a solution of KHMDS (0.91 M in THF, 32.6 mL, 29.7 mmol) dropwise over 10 min. After the addition was complete the reaction mixture was maintained for 30 min at −78 °C. Then a solution of Tf_2NPh (3.9 g, 11 mmol) in THF (30 mL) was added dropwise over 10 min. The reaction was stirred at −78 °C for 1 h. The reaction mixture was then warmed up to room temperature and stirred for an additional 16 h. Methanol (15 mL) was then added and the reaction mixture was concentrated *in vacuo*. Purification by flash column chromatography (0% to 20% EtOAc/petrol) yielded methyl 1-fluorocyclooct-2-ynecarboxylate (1.3 g, 7.4 mmol, 75%) as a yellow oil: ^{1}H NMR (500 MHz, $CDCl_3$) δ 3.83 (s, 3H), 2.41–2.20 (m, 4H), 2.06–1.85 (m, 4H), 1.77–1.66 (m, 1H), 1.50–1.42 (m, 1H); ^{13}C NMR (125 MHz, $CDCl_3$) δ 168.8 (d, $^{2}J_{C\text{-}F}$ = 27.8 Hz, C), 108.5 (d, $^{3}J_{C\text{-}F}$ = 9.6 Hz, C), 91.7 (d, $J_{C\text{-}F}$ = 186.2 Hz, C), 86.9 (d, $^{2}J_{C\text{-}F}$ = 31.7 Hz, C), 53.4 (CH_3), 46.2 (d, $^{2}J_{C\text{-}F}$ = 25.0 Hz, CH_2), 33.9 (CH_2), 29.2 (CH_2), 25.6 (CH_2), 20.7 (CH_2); IR (thin film) 2930, 2854, 2222, 1751 cm^{-1}.

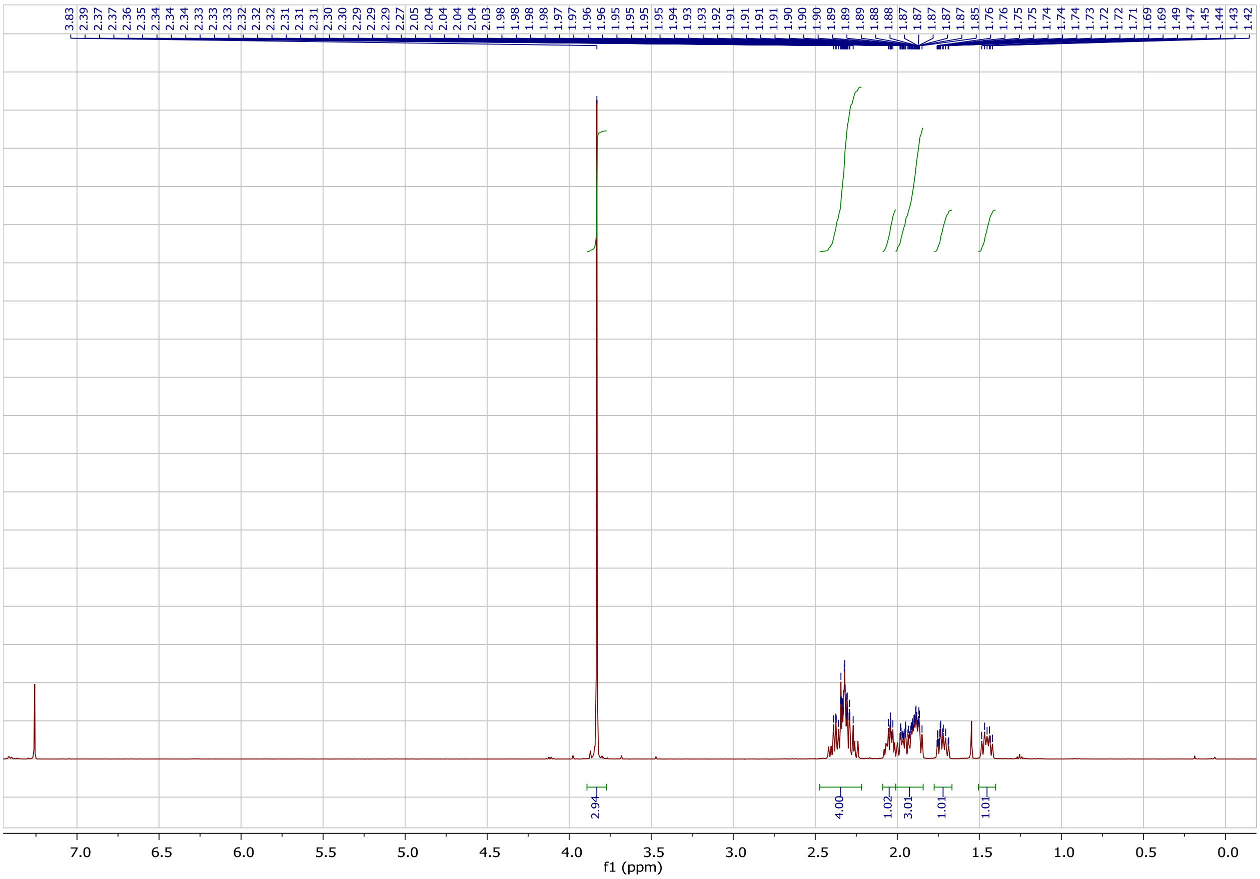

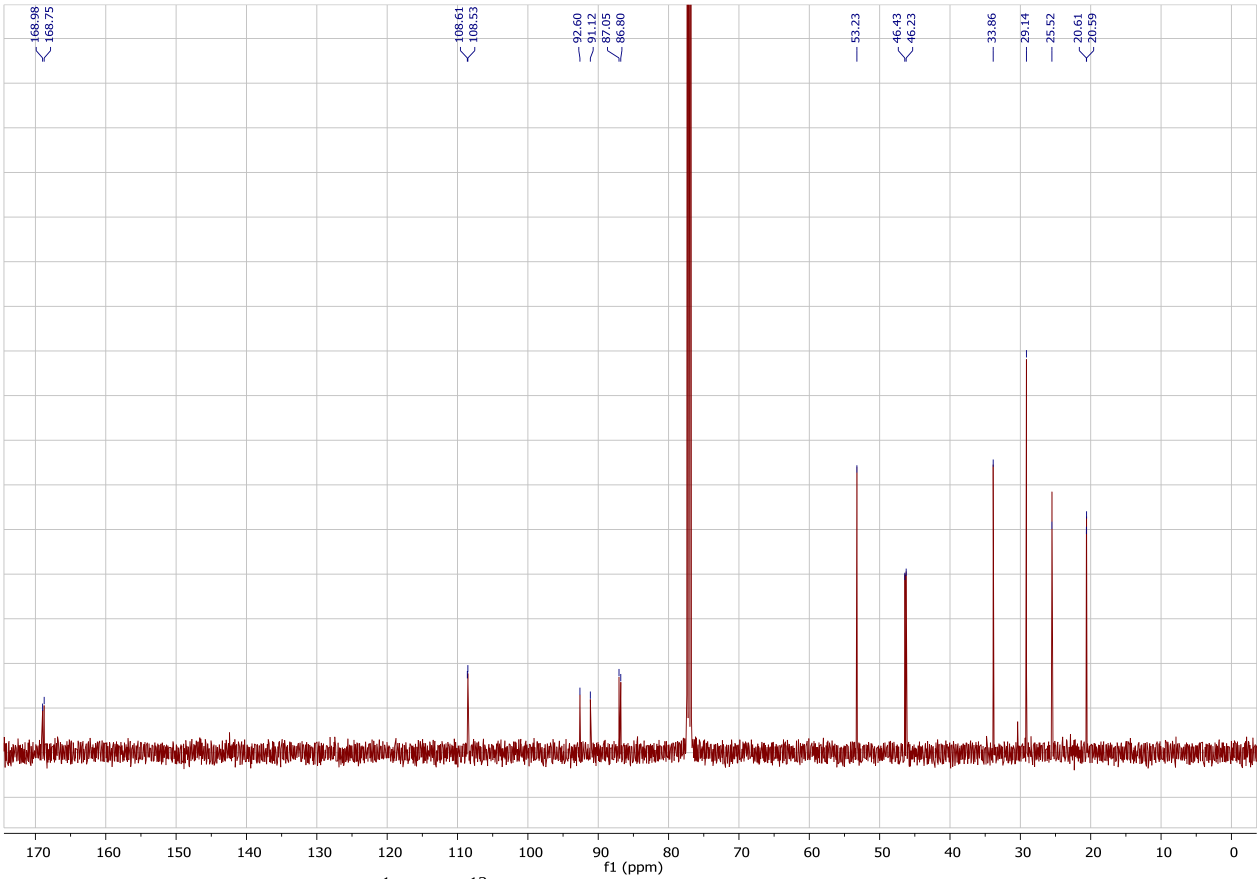

Supplementary Figure 6 ^{1}H and ^{13}C NMR data for methyl 1-fluorocyclooct-2-ynecarboxylate.

***Tert*-butyl (1-(cyclooct-2-yn-1-yl)-1-oxo-6,9,12-trioxa-2-azapentadecan-15-yl)carbamate**[5]

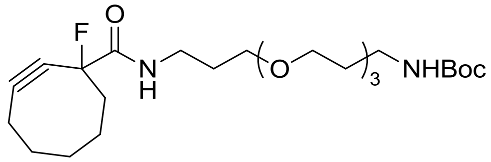

To a solution of methyl 1-fluorocyclooct-2-ynecarboxylate (1.0 g, 5.0 mmol) in triethylamine (6 mL) was added *tert*-butyl (3-(2-(2-(3-aminopropoxy)ethoxy)ethoxy)propyl)carbamate (4.8 g, 15 mmol). The reaction mixture was stirred at 25 °C for 48 h. Purification by flash column chromatography (0% to 50% EtOAc/petrol) yielded *tert*-butyl (1-(cyclooct-2-yn-1-yl)-1-oxo-6,9,12-trioxa-2-azapentadecan-15-yl)carbamate (1.4 g, 3.1 mmol, 63 %) as a yellow oil: ^{1}H NMR (600 MHz, $CDCl_3$) δ 7.00 (br s, 1H), 4.98 (br s, 1H), 3.62–3.58 (m, 4H), 3.58–3.52 (m, 6H), 3.49 (t, J = 6.0 Hz, 2H), 3.37 (q, J = 6.0 Hz, 2H), 3.18 (q, J = 5.5 Hz, 2H), 2.43–2.16 (m, 4H), 2.07–1.98 (m, 1H), 1.98–1.87 (m, 2H), 1.86–1.66 (m, 5H), 1.66–1.58 (m, 1H), 1.44–1.33 (m, 10H); ^{13}C NMR (150 MHz, $CDCl_3$) δ 168.1 (d, $^{2}J_{C\text{-}F}$ = 24.1 Hz, C), 155.9 (C), 108.8 (d, $^{3}J_{C\text{-}F}$ = 10.5 Hz, C), 94.3 (d, $J_{C\text{-}F}$ = 186.4 Hz, C), 87.4 (d, $^{2}J_{C\text{-}F}$ = 31.6 Hz, C), 78.7 (CH_2), 70.5 (CH_2), 70.4 (CH_2), 70.3 (CH_2), 70.1 (CH_2), 70.1 (CH_2), 69.5 (CH_2), 46.2 (d, $^{2}J_{C\text{-}F}$ = 24.1 Hz, CH_2), 38.5 (CH_2), 38.1 (CH_2), 33.8 (CH_2), 29.5 (CH_2), 28.8 (CH_2), 28.6 (CH_2), 28.3 (CH_3), 25.6 (CH_2), 20.5 (CH_2); IR (thin film) 3345, 2927, 2864, 2228, 1685, 1679 cm^{-1}; LRMS (CI) 473 (25, $[M+H]^+$), 373 (100, $[M-C_5H_8O_2]^+$); HRMS (CI) calcd for $C_{24}H_{42}FO_6N_2$ $[M+H]^+$ 473.3027, observed 473.3023.

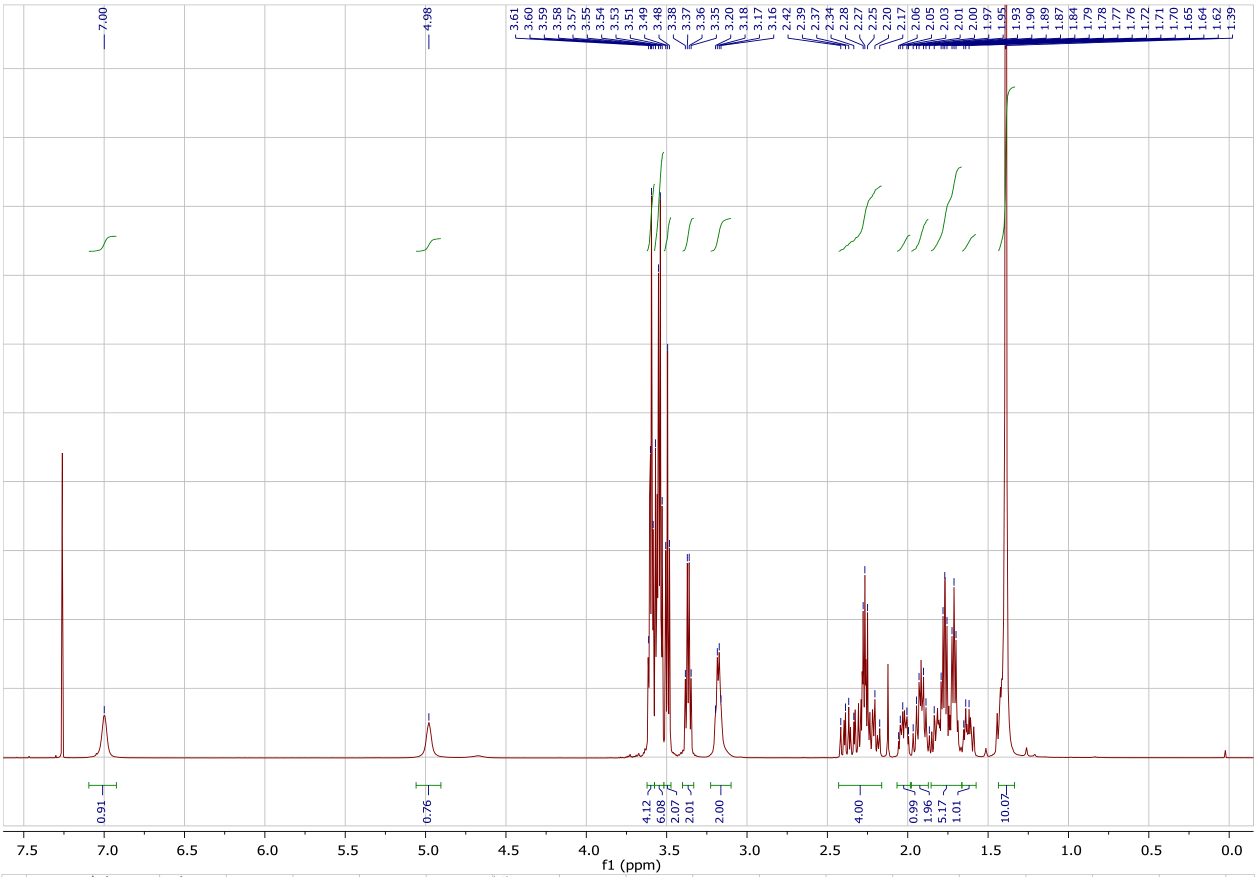

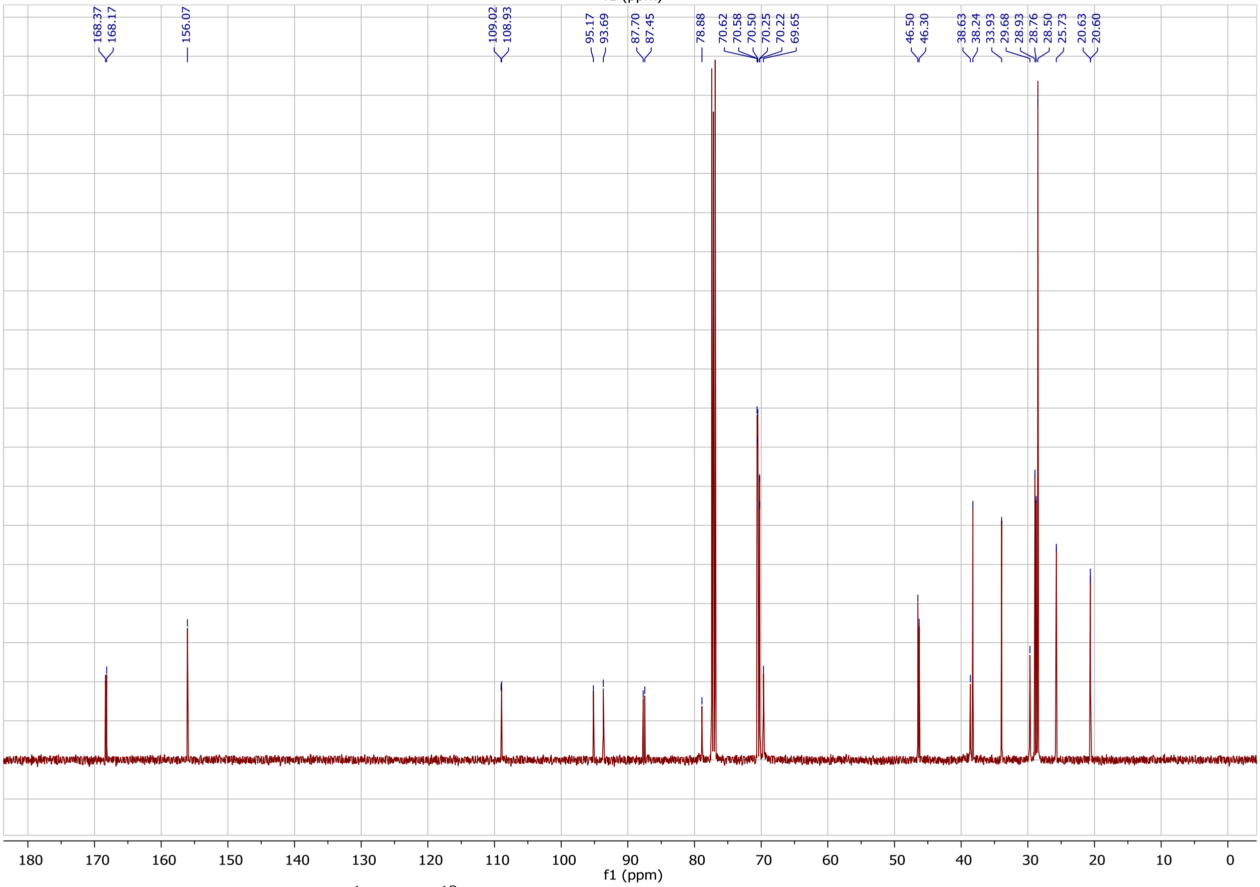

Supplementary Figure 7 ^{1}H and ^{13}C NMR data for *tert*-butyl (1-(cyclooct-2-yn-1-yl)-1-oxo-6,9,12-trioxa-2-azapentadecan-15-yl)carbamate.

***N*-(1-(4,5-Dibromo-3,6-dioxo-2-(prop-2-yn-1-yl)-2,3-dihydropyridazin-1(6*H*)-yl)-2-oxo-7,10,13-trioxa-3-azahexadecan-16-yl)-1-fluorocyclooct-2-ynecarboxamide**

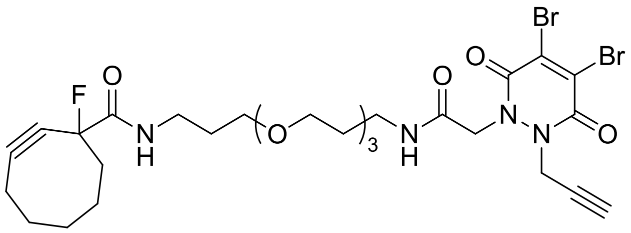

To a solution of *tert*-butyl (1-(cyclooct-2-yn-1-yl)-1-oxo-6,9,12-trioxa-2-azapentadecan-15-yl)carbamate (50 mg, 0.11 mmol) in CH_2Cl_2 (3 mL) was added TFA (1.5 mL) dropwise at −20 °C and the reaction mixture stirred at −20 °C for 2 h. After this time, all volatile material was removed *in vacuo*. The crude residue was then dissolved in CH_2Cl_2 (2 mL) and was added to a solution of 2-(4,5-dibromo-3,6-dioxo-2-(prop-2-yn-1-yl)-2,3-dihydropyridazin-1(6*H*)-yl)acetic acid (39 mg, 0.11 mmol), EDC·HCl (22 mg, 0.12 mmol), and NEt_3 (24 mg, 0.23 mmol) in CH_2Cl_2 (3 mL). The resulting solution was stirred at 21 °C for 4 h. Then the reaction mixture was diluted with H_2O (15 mL), extracted with EtOAc (3 × 15 mL), the combined organic layers were dried ($MgSO_4$) and concentrated *in vacuo*. The crude residue was purified by flash column chromatography (5% MeOH/ CH_2Cl_2) to afford *N*-(1-(4,5-dibromo-3,6-dioxo-2-(prop-2-yn-1-yl)-2,3-dihydropyridazin-1(6*H*)-yl)-2-oxo-7,10,13-trioxa-3-azahexadecan-16-yl)-1-fluorocyclooct-2-ynecarboxamide (16 mg, 0.02 mmol, 20%) as a yellowish oil: ^{1}H NMR (600 MHz, $CDCl_3$) δ 7.10 (br s, 1H), 7.00 (br s, 1H), 5.01 (d, *J* = 2.4 Hz, 2H), 4.88 (s, 2H), 3.69–3.64 (m, 4H), 3.63–3.55 (m, 8H), 3.40 (q, *J* = 5.8 Hz, 4H), 2.44 (t, *J* = 2.4 Hz, 1H), 2.42–2.20 (m, 4H), 2.11–2.03 (m, 1H), 2.02–1.89 (m, 2H), 1.89–1.75 (m, 6H), 1.49–1.39 (m, 1H); ^{13}C NMR (150 MHz, $CDCl_3$) δ 168.5 (d, $^{2}J_{C\text{-}F}$ = 24.1 Hz, C), 165.3 (C), 153.6 (C), 152.4 (C), 136.4 (C), 135.9 (C), 109.4 (d, $^{3}J_{C\text{-}F}$ = 10.6 Hz, C), 94.6 (d, $J_{C\text{-}F}$ = 186.4 Hz, C), 87.5 (d, $^{2}J_{C\text{-}F}$ = 31.6 Hz, C), 76.3 (C), 74.9 (CH), 70.5 (CH_2), 70.5 (CH_2), 70.4 (CH_2), 70.1 (CH_2), 70.1 (CH_2), 69.9 (CH_2), 50.5 (CH_2), 46.5 (d, $^{2}J_{C\text{-}F}$ = 24.5 Hz, CH_2), 38.8 (CH_2), 38.2 (CH_2), 37.3 (CH_2), 34.0 (CH_2), 29.0 (CH_2), 28.9 (CH_2), 28.5 (CH_2), 25.8 (CH_2), 20.7 (CH_2); IR (thin film) 3301, 2927, 2867, 2123, 1646 cm^{-1}; LRMS (ES^+) 723 (50, $[M^{81}Br^{81}Br+H]^+$), 721 (100, $[M^{81}Br^{79}Br+H]^+$), 719 (50, $[M^{79}Br^{79}Br+H]^+$); HRMS (ES^+) calcd for $C_{28}H_{38}O_7N_4{}^{79}Br_2F$ $[M^{79}Br_2+H]^+$ 719.1091, observed 719.1084.

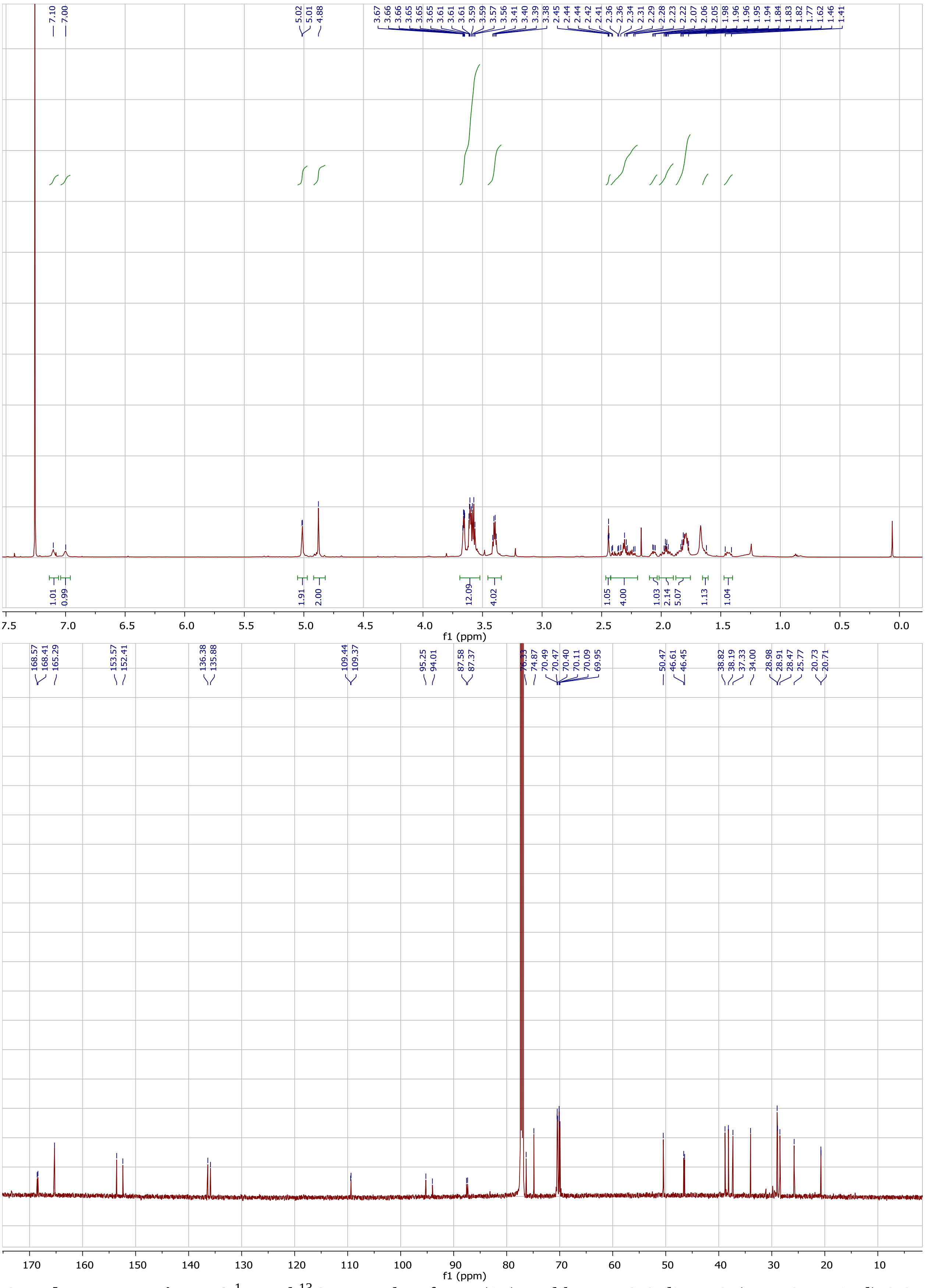

Supplementary Figure 8 ^{1}H and ^{13}C NMR data for *N*-(1-(4,5-dibromo-3,6-dioxo-2-(prop-2-yn-1-yl)-2,3-dihydropyridazin-1(6*H*)-yl)-2-oxo-7,10,13-trioxa-3-azahexadecan-16-yl)-1-fluorocyclooct-2-ynecarboxamide.

14-Azido-*N*-((2*S*,3*S*,4*S*,6*R*)-3-hydroxy-2-methyl-6-(((1*S*,3*S*)-3,5,12-trihydroxy-3-(2-hydroxyacetyl)-10-methoxy-6,11-dioxo-1,2,3,4,6,11-hexahydrotetracen-1-yl)oxy)tetrahydro-2*H*-pyran-4-yl)-3,6,9,12-tetraoxatetradecan-1-amide (Dox- N_3)

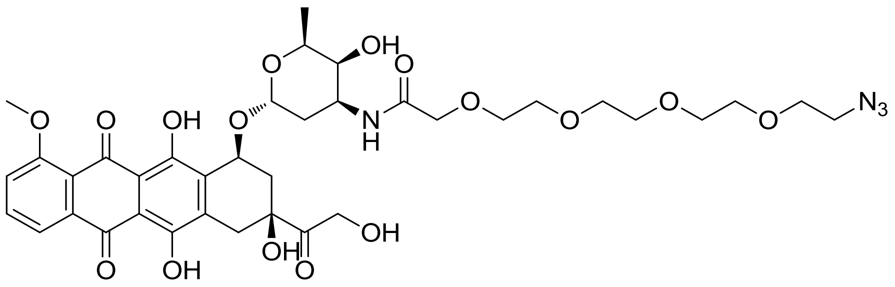

To a solution of 14-azido-3,6,9,12-tetraoxatetradecan-1-oic acid[6] (4.4 mg, 16 µmol), and DIPEA (6.2 µL, 35 µmol) in DMF (1 mL) was added HBTU (6.7 mg, 18 µmol) and the reaction mixture stirred at 21 °C for 5 min. After this time, doxorubicin (9.3 mg, 16 µmol) was added and the reaction mixture stirred at 21 °C for 3 h. Then, the reaction mixture was diluted with H_2O (10 mL) and CH_2Cl_2 (10 mL), extracted with CH_2Cl_2 (3 × 15 mL), the combined organic layers washed with sat. aq. LiCl (2 × 10 mL) and acetate buffer pH 5 (10 mL), dried ($MgSO_4$) and concentrated *in vacuo* using toluene as an azeotrope. The crude residue was purified by flash column chromatography (5% MeOH/EtOAc) to afford 14-azido-*N*-((2*S*,3*S*,4*S*,6*R*)-3-hydroxy-2-methyl-6-(((1*S*,3*S*)-3,5,12-trihydroxy-3-(2-hydroxyacetyl)-10-methoxy-6,11-dioxo-1,2,3,4,6,11-hexahydrotetracen-1-yl)oxy)tetrahydro-2*H*-pyran-4-yl)-3,6,9,12-tetraoxatetradecan-1-amide (9.0 mg, 11 µmol, 70%) as a red solid: m.p. = 61–64 °C; ^{1}H NMR (600 MHz, MeOD-d_4 + drops of $CDCl_3$) δ 7.97 (dd, *J* = 7.8, 1.2 Hz, 1H), 7.74 (t, *J* = 7.8 Hz, 1H), 7.36 (d, *J* = 8.4 Hz, 1H), 7.24 (br, 1H), 5.48 (d, *J* = 4.2 Hz, 1H), 5.24 (dt, *J* = 3.6, 1.8 Hz, 1H), 4.74 (ABq, *J* = 21.0 Hz, ν_{AB} = 8.9 Hz, 2H), 4.17 (m, 1H), 4.11 (q, *J* = 6.6 Hz, 1H), 4.05 (s, 3H), 3.91 (ABq, *J* = 16.2 Hz, ν_{AB} = 16.9 Hz, 2H), 3.62–3.70 (m, 15H), 3.39 (t, *J* = 5.4 Hz, 2H), 3.18 (d, *J* = 18.6 Hz, 1H), 2.90 (d, *J* = 18.6 Hz, 1H), 2.33 (d, *J* = 14.4 Hz, 1H), 2.12–2.15 (dd, *J* = 14.4, 4.2 Hz, 1H), 1.92 (dt, *J* = 13.2, 4.2 Hz, 1H), 1.75–1.78 (dd, *J* = 13.2, 4.8 Hz, 1H), 1.27 (d, *J* = 6.6 Hz, 3H); ^{13}C NMR (150 MHz, MeOD-d_4 + drops of $CDCl_3$) δ 214.1 (C), 187.0 (C), 186.6 (C), 169.4 (C), 161.1 (C), 156.3 (C), 155.7 (C), 135.8 (CH), 135.5 (C), 133.8 (C), 133.7 (C), 120.8 (C), 119.9 (CH), 118.5 (CH), 111.5 (C), 111.4 (C), 101.1 (CH), 76.7 (C), 71.0 (CH_2), 70.8 (CH_2), 70.6 (CH_2), 70.5 (CH_2), 70.4 (CH_2), 70.2 (CH_2), 70.1 (CH_2), 69.7 (CH), 69.2 (CH), 67.6 (CH), 65.7 (CH_2), 56.8 (CH_3), 50.7 (CH_2), 45.0 (CH), 35.7 (CH_2), 34.0 (CH_2), 29.7 (CH_2), 17.1 (CH_3); IR (solid) 3405, 3341, 2917, 2100, 1650, 1615 cm^{-1}; LRMS (ESI) 825 (100, $[M+Na]^+$); HRMS (ESI) calcd. for $C_{37}H_{46}N_4O_{16}Na$ $[M+Na]^+$ 825.2808, observed: 825.2807.

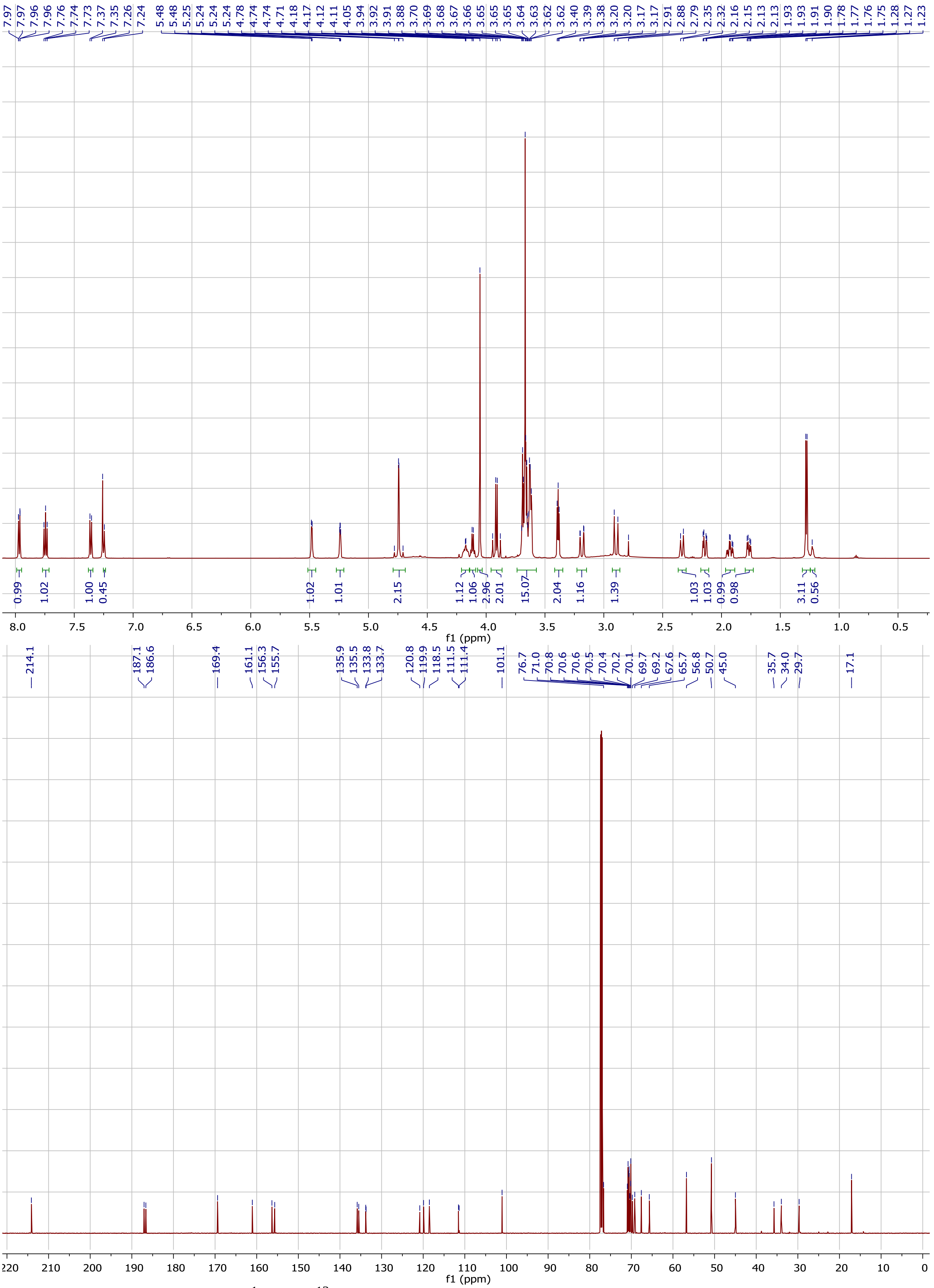

Supplementary Figure 9 ^{1}H and ^{13}C NMR data for 14-azido-*N*-((2*S*,3*S*,4*S*,6*R*)-3-hydroxy-2-methyl-6-(((1*S*,3*S*)-3,5,12-trihydroxy-3-(2-hydroxyacetyl)-10-methoxy-6,11-dioxo-1,2,3,4,6,11-hexahydrotetracen-1-yl)oxy)tetrahydro-2H-pyran-4-yl)-3,6,9,12-tetraoxatetradecan-1-amide (Dox-N_3).

4,5-Dibromo-1,2-diethyl-1,2-dihydropyridazine-3,6-dione (Diet-PD)[7]

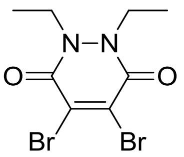

To a solution of *N*,*N'*-diethyl-hydrazine (88 mg, 1.0 mmol) in glacial AcOH (5 mL) 2,3-dibromomaleic anhydride[2] (0.25 g, 1.0 mmol), and the reaction mixture heated at 130 °C for 16 h. Then the reaction mixture was concentrated *in vacuo*, and purification by flash column chromatography (50% EtOAc/Petrol to neat EtOAc) yielded 4,5-dibromo-1,2-diethyl-1,2-dihydro-pyridazine-3,6-dione (0.20 g, 0.61 mmol, 61%) as a yellow solid: ^{1}H NMR (600 MHz, $CDCl_3$) δ 4.17 (q, *J* = 7.0 Hz, 4H), 1.28 (t, *J* = 7.0 Hz, 6H); ^{13}C NMR (150 MHz, $CDCl_3$) δ 153.3 (C), 136.1 (C), 42.4 (CH_2), 13.2 (CH_3); IR (solid) 2979, 2937, 1630, 1574 cm^{-1}; LRMS (EI) 328 (50, $[M^{81}Br^{81}Br]^{+\bullet}$), 326 (100, $[M^{81}Br^{79}Br]^{+\bullet}$), 324 (50, $[M^{79}Br^{79}Br]^{+\bullet}$); HRMS (EI) calcd for $C_8H_{10}Br_2N_2O_2$ $[M^{79}Br^{79}Br]^{+\bullet}$ 323.9104, observed 323.9097.

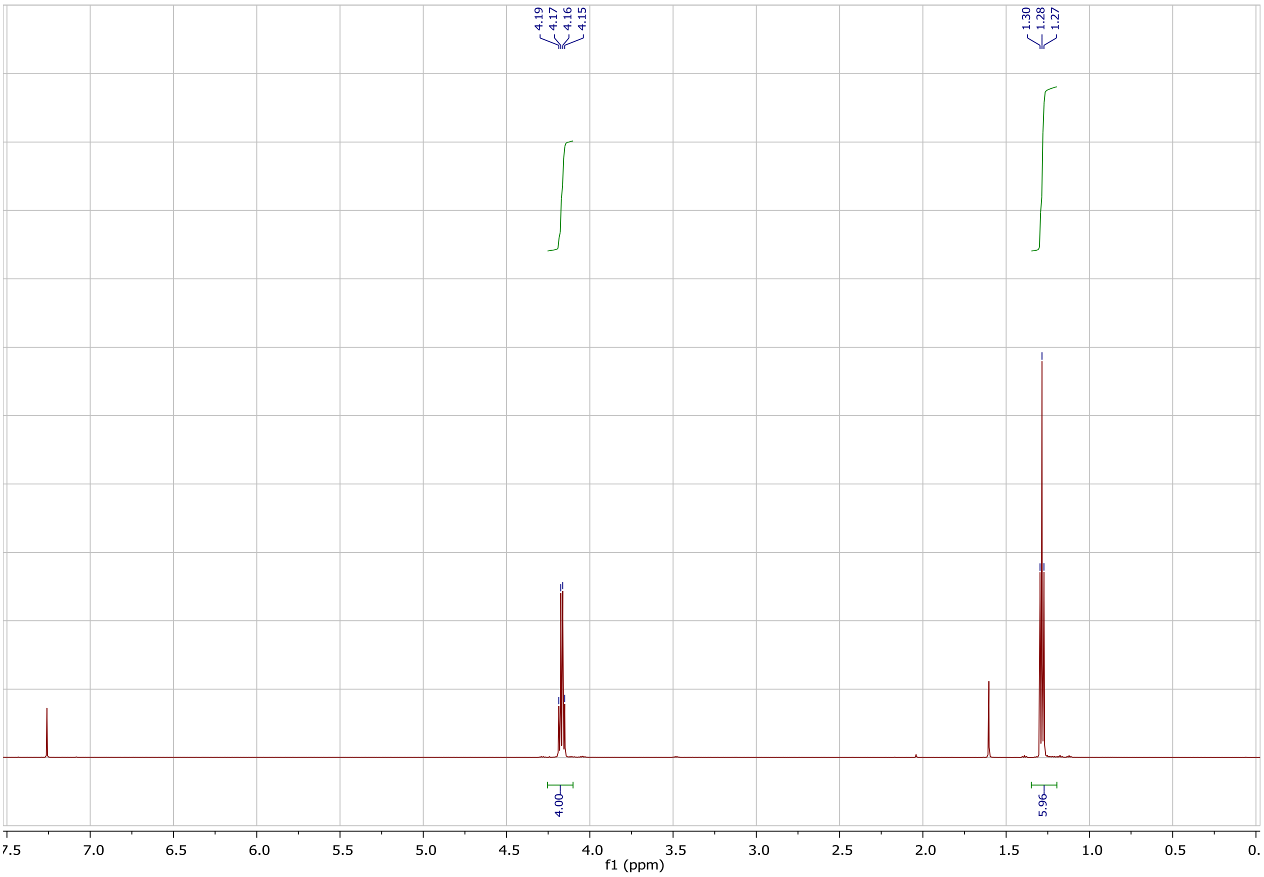

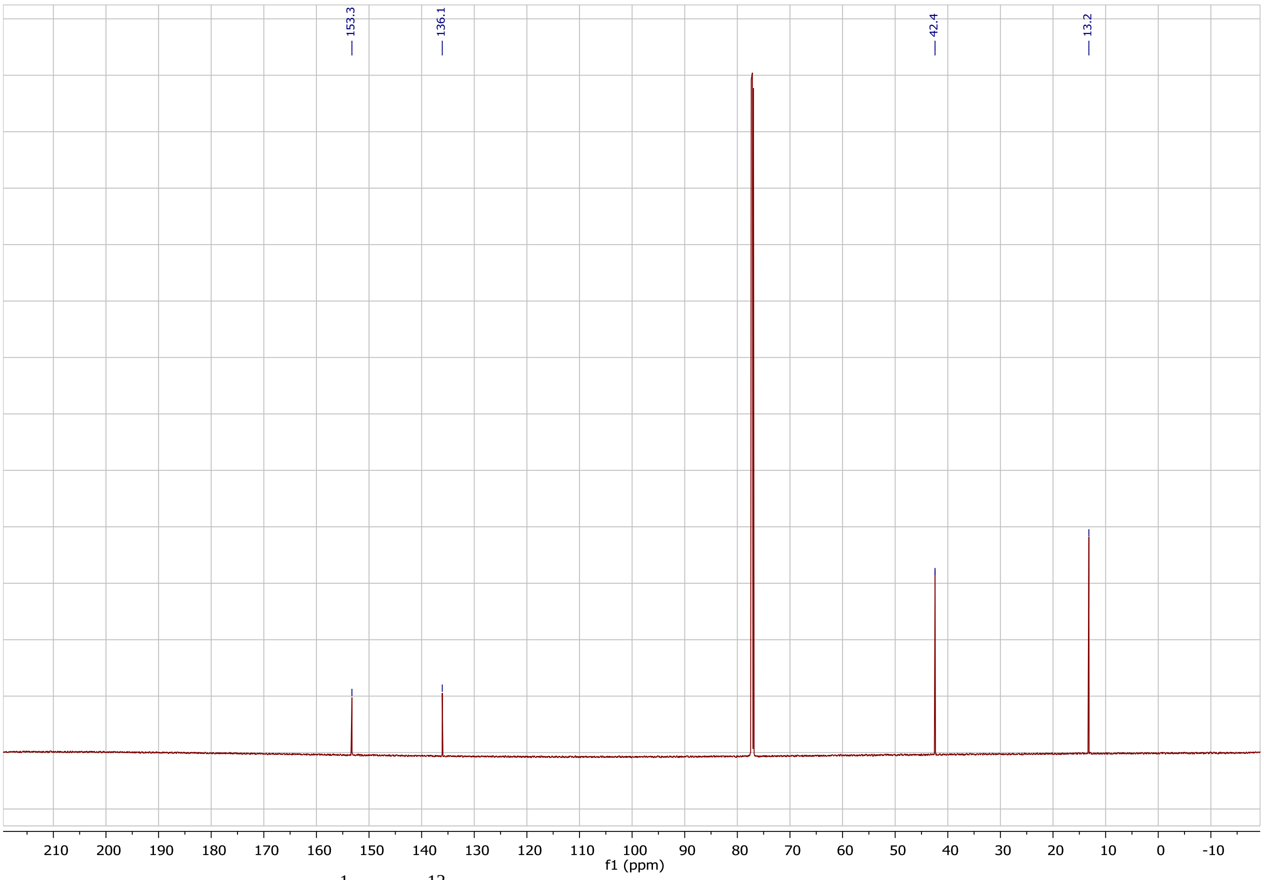

Supplementary Figure 10 ^{1}H and ^{13}C NMR data for 4,5-dibromo-1,2-diethyl-1,2-dihydropyridazine-3,6-dione (Diet-PD).

Trastuzumab Fab fragment 2 preparation[3]

Preparation of Fab-Her fragment using sequential digests with pepsin and papain

Immobilized pepsin (0.15 mL) was washed with digestion buffer (20 mM sodium acetate trihydrate, pH 3.1) four times and trastuzumab (0.5 mL, 6.41 mg·mL^{-1} in digestion buffer) was added. The mixture was incubated for 5 h at 37 °C whilst shaking (1100 rpm). The resin was separated from the digest using a filter column, and washed with digest buffer (50 mM phosphate, 1 mM EDTA, 150 mM NaCl, pH 6.8) three times. The digest was combined with the washes and the volume adjusted to 0.5 mL.

After this, immobilized papain (0.5 mL, 0.25 mg·mL^{-1}) was activated with 10 mM DTT (in digest buffer: 50 mM phosphate, 1 mM EDTA, 150 mM NaCl, pH 6.8) whilst shaking (1100 rpm) for 1 h at 37 °C. The resin was washed with digest buffer (without DTT) four times and the 0.5 mL of Herceptin-F(ab')$_2$ added. The mixture was incubated for 16 h at 37 °C whilst shaking (1100 rpm). Then the resin was separated from the digest using a filter column, and washed with BBS (25 mM sodium borate, 25 mM NaCl, 0.5 mM EDTA, pH 8.0) three times. The digest was combined with the washes and the buffer was exchanged completely for BBS using diafiltration columns (GE Healthcare, 10000 MWCO) and the volume adjusted to 0.5 mL. The digest was analysed by SDS-PAGE and LCMS to reveal formation of a single trastuzumab Fab fragment: observed mass 47652. The concentration of Fab-Her fragment **2** was determined by UV/VIS using a molecular extinction coefficient of ε_{280} = 68590 M^{-1}·cm^{-1}. [Fab-Her fragment] 2.8 mg·mL^{-1} (0.5 mL), 67%.

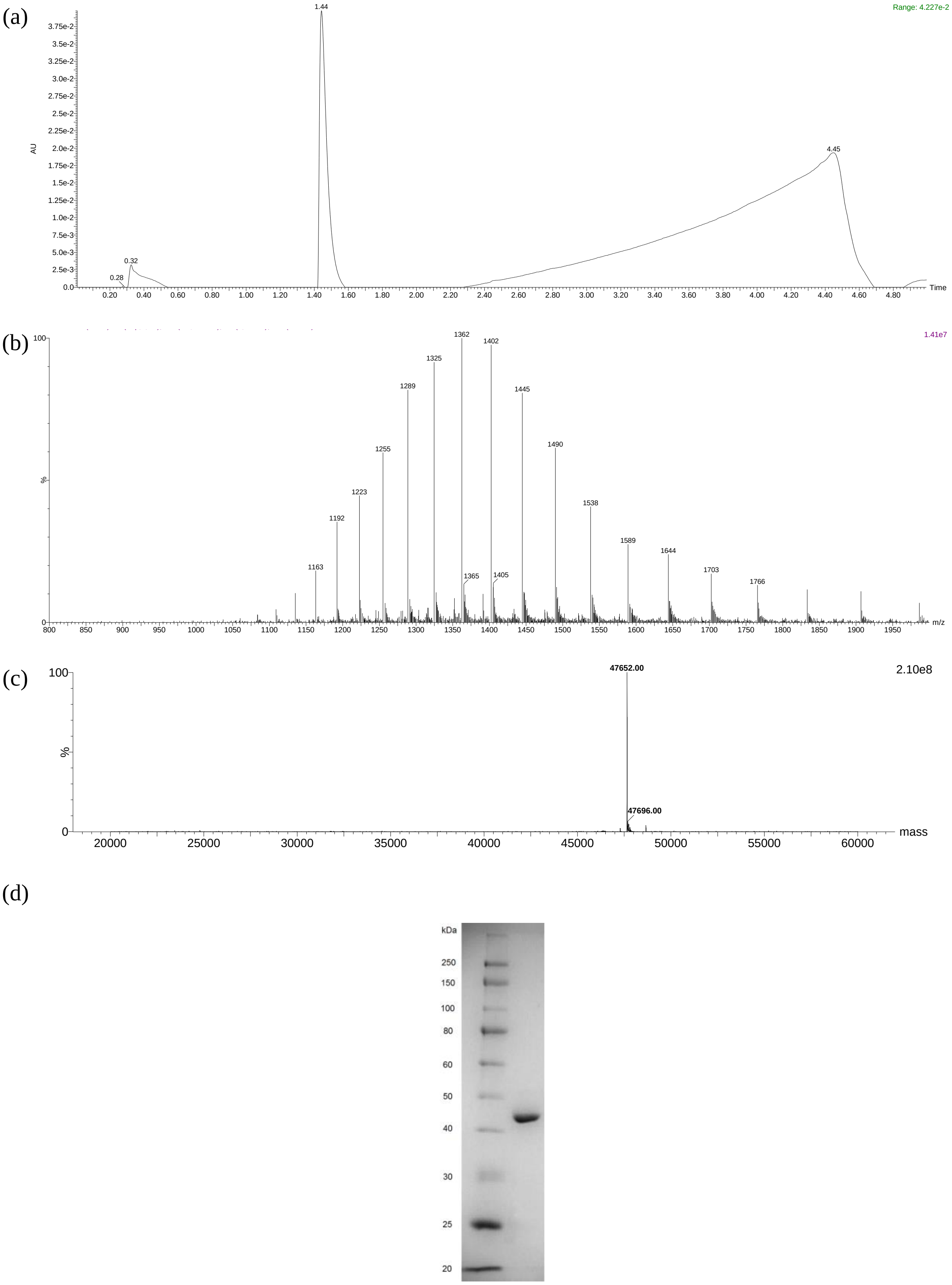

Supplementary Figure 11 (**a**) Absorbance at 254 nm, (**b**) non-deconvoluted, (**c**) deconvoluted MS data for Fab-Her fragment **2** and (**d**) SDS-PAGE characterisation of Her-Fab **2** prepared by using sequential digests with pepsin and papain.

Bioconjugation reactions involving Fab-Her fragment 2

Formation of bioconjugate 3

To a solution of Fab-Her **2** (50 µL, 30 µM, 1.4 mg·mL^{-1}, 1 eq) in borate buffer (25 mM sodium borate, 25 mM NaCl, 0.5 mM EDTA, pH 8.0) was added TCEP (final concentration 90 µM, 3 eq) and the reaction mixture incubated at 37 °C for 90 min. After this time, was added a solution of Astra-PD **1** in DMF (final concentration 1.5 mM, 5 eq) and the reaction mixture incubated at 37 °C for 1 h. The excess reagents were then removed by repeated diafiltration into fresh buffer using VivaSpin sample concentrators (GE Healthcare, 10000 MWCO). Following this, analysis by LCMS revealed conversion to the desired trastuzumab Fab-Astra bioconjugate **3** in an average >95% yield (expected mass: 48212 Da, observed mass: 48211 Da).

(a)

(b)

(c)

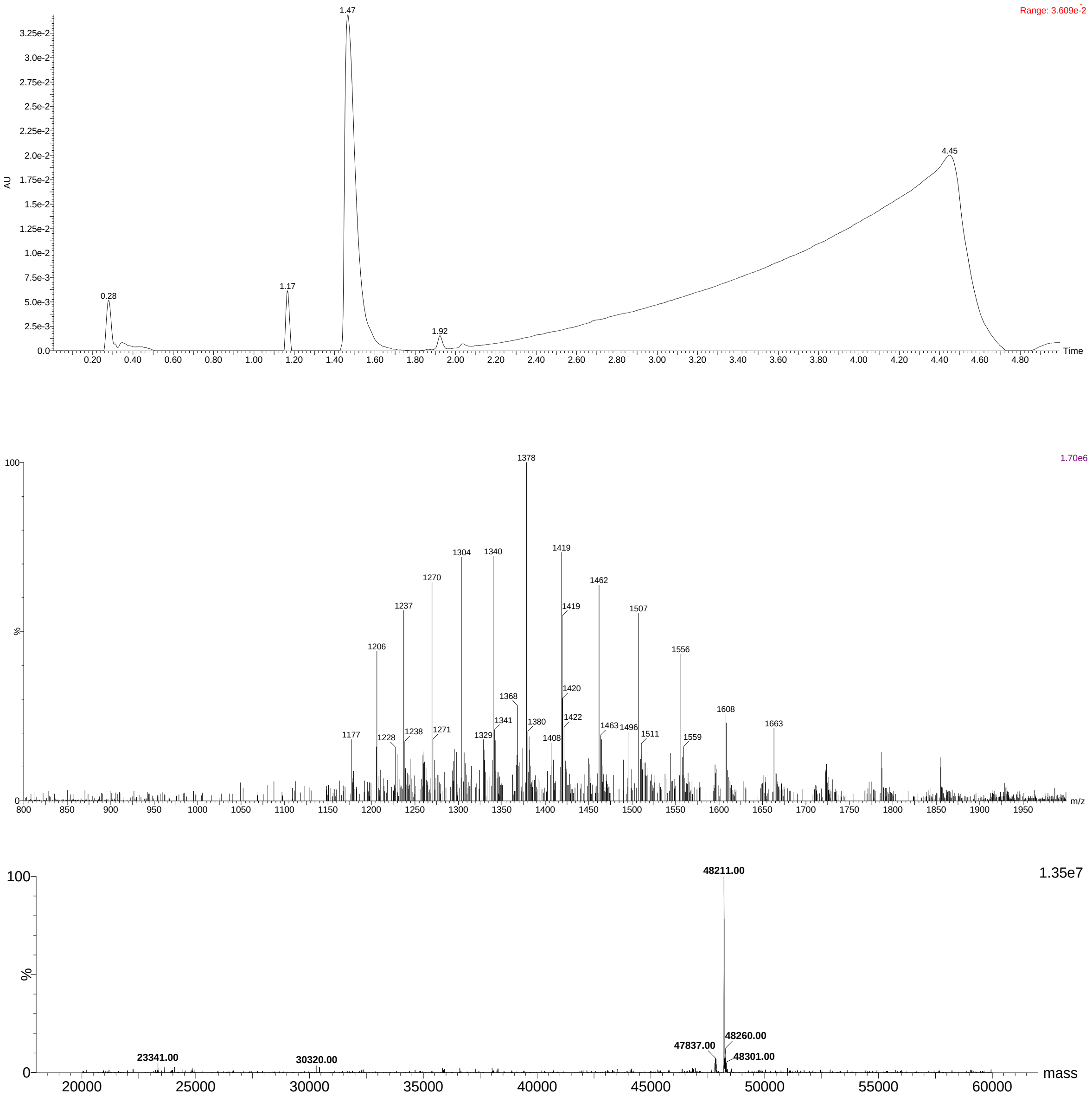

Supplementary Figure 12 (**a**) Absorbance at 254 nm, (**b**) non-deconvoluted, and (**c**) deconvoluted MS data for bioconjugate **3**.

Formation of bioconjugate 4

To a solution of trastuzumab Fab-Astra **3** (50 µL, 30 µM, 1.4 mg·mL^{-1}, 1 eq) in PBS (pH 7.4) was added 2-(2-(2-(2-azidoethoxy)ethoxy)ethoxy)ethanamine (final concentration 150 µM, 5 eq) and the reaction mixture incubated at 37 °C for 4 h. The excess reagents were then removed by repeated diafiltration into fresh buffer using VivaSpin sample concentrators (GE Healthcare, 10000 MWCO). Following this, analysis by LCMS revealed conversion to the desired trastuzumab Fab-Astra-PEG$_4$ bioconjugate **4** in an average >95% yield (expected mass: 48431 Da, observed mass: 48432 Da).

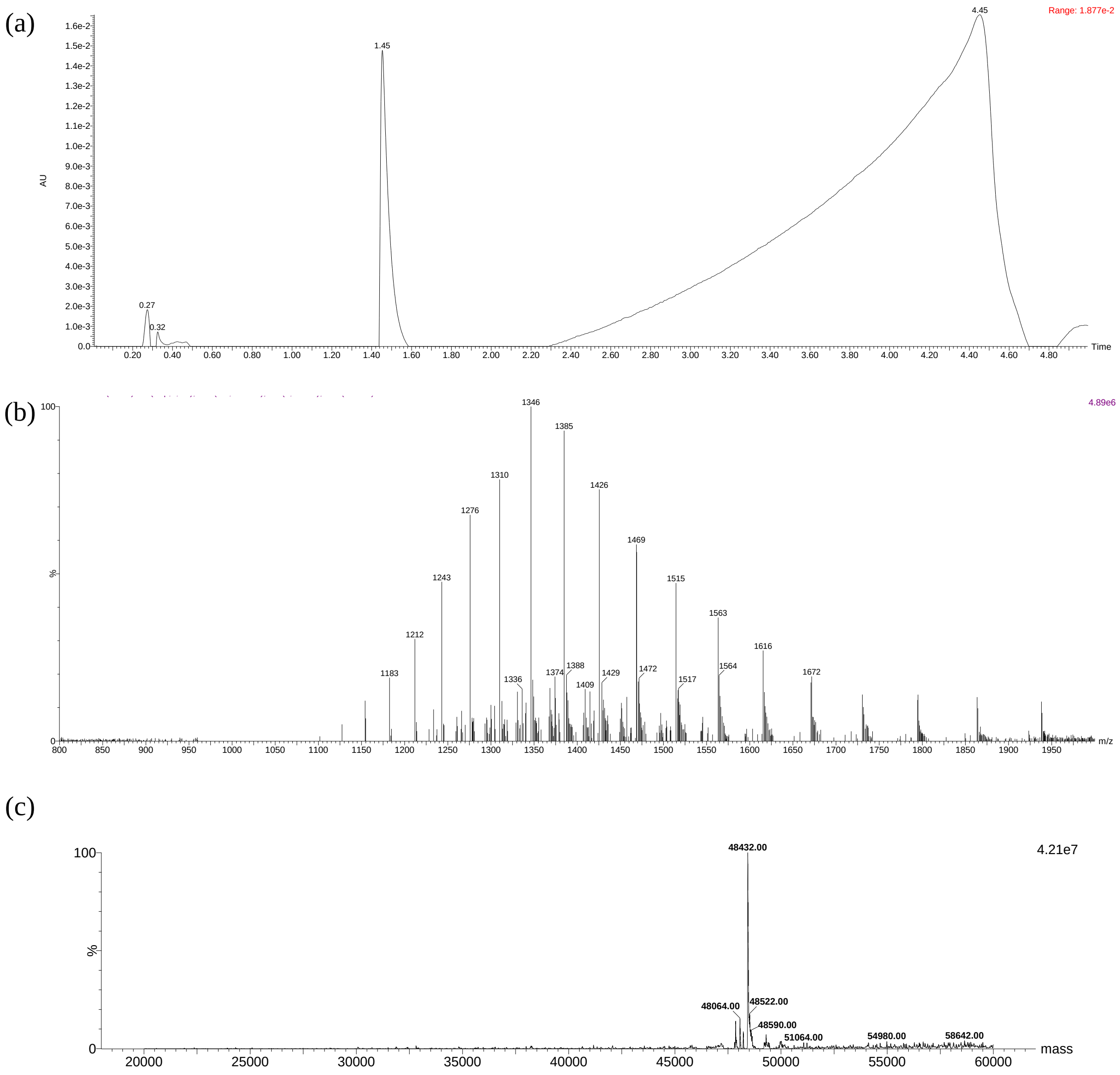

Supplementary Figure 13 (**a**) Absorbance at 254 nm, (**b**) non-deconvoluted, and (**c**) deconvoluted MS data for bioconjugate **4**.

Formation of bioconjugate 5

To a solution of trastuzumab Fab-Astra-PEG$_4$ bioconjugate **4** (50 µL, 50 µM, 1 eq) in PBS (pH 7.4) containing tris(3-hydroxypropyltriazolylmethyl)amine (THPTA) (500 µM), $CuSO_4$ (100 µM) was added sulfo-Cy5-azide (final concentration 150 µM, 3 eq) and sodium ascorbate (final concentration 2.5 mM) and the reaction mixture incubated at 21 °C for 2 h. The excess reagents were then removed by repeated diafiltration into fresh PBS with 2 mM EDTA (to remove residual copper ions) using VivaSpin sample concentrators (GE Healthcare, 10000 MWCO). Following this, analysis by LCMS revealed conversion to the desired trastuzumab Fab-Astra-PEG$_4$-Cy5 bioconjugate **5** in an average 80% yield (expected mass: 49176 Da, observed mass: 49175 Da).

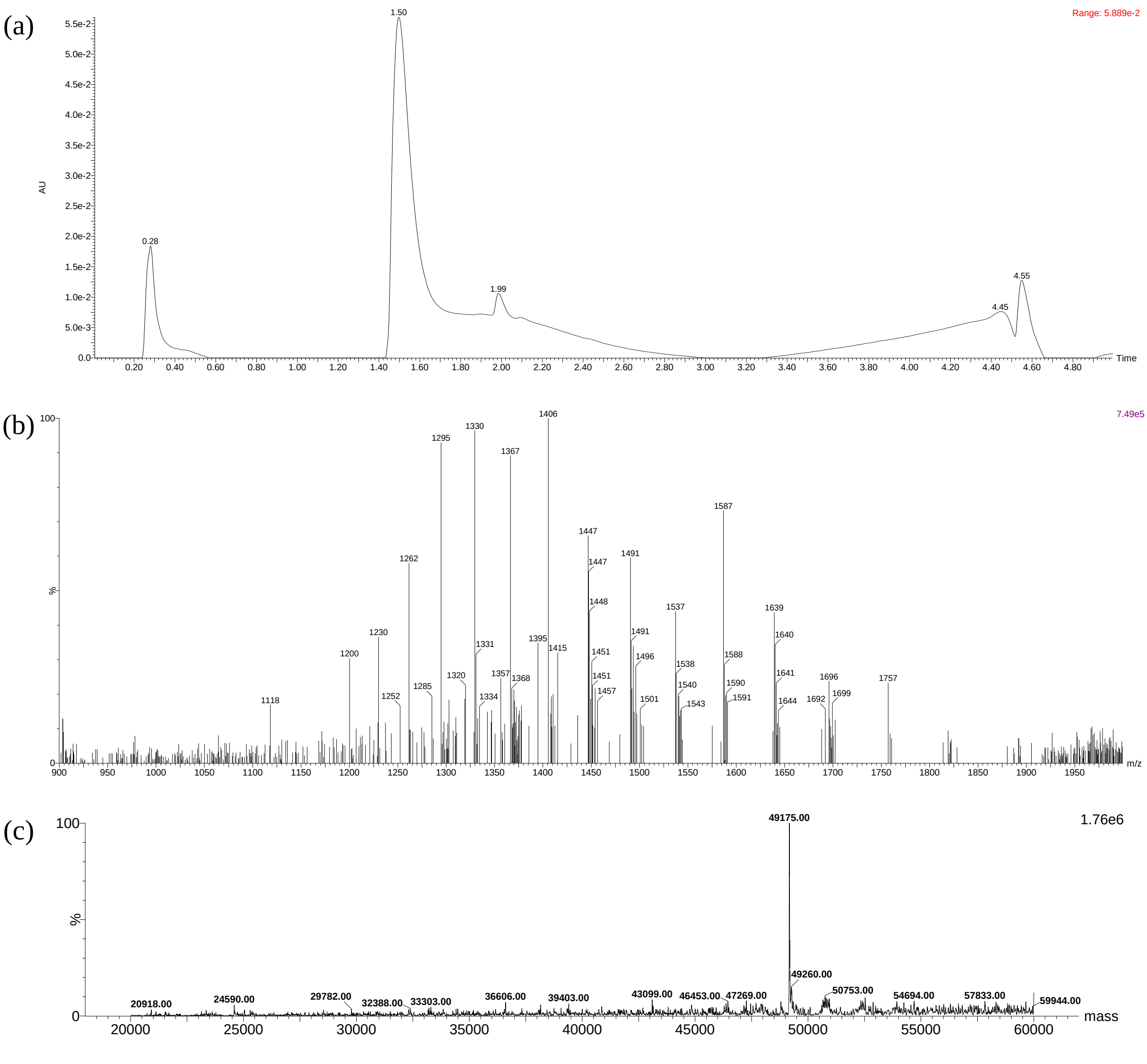

Supplementary Figure 14 (**a**) Absorbance at 254 nm, (**b**) non-deconvoluted, and (**c**) deconvoluted MS data for bioconjugate **5**.

Formation of bioconjugate 6

To a solution of Fab-Her **2** (50 µL, 30 µM, 1.4 mg·mL^{-1}, 1 eq) in borate buffer (25 mM sodium borate, 25 mM NaCl, 0.5 mM EDTA, pH 8.0) was added TCEP (final concentration 90 µM, 3 eq) and the reaction mixture incubated at 37 °C for 90 min. After this time, was added a solution of Diet-PD in DMF (final concentration 1.5 mM, 5 eq) and the reaction mixture incubated at 37 °C for 1 h. The excess reagents were then removed by repeated diafiltration into fresh buffer using VivaSpin sample concentrators (GE Healthcare, 10000 MWCO). Following this, analysis by LCMS revealed conversion to the desired trastuzumab Fab-Diet bioconjugate **6** in an average >95% yield (expected mass: 47820 Da, observed mass: 47823 Da).

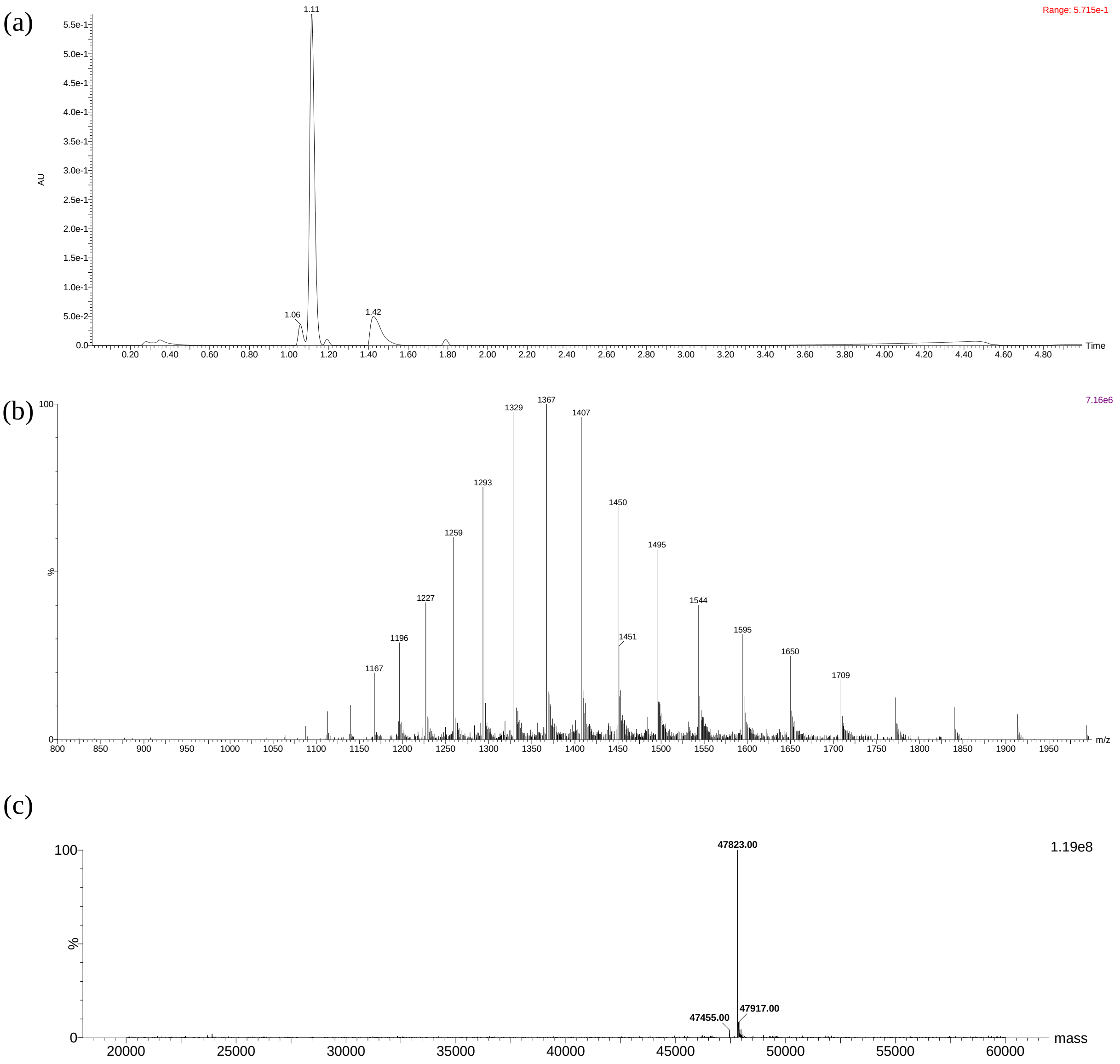

Supplementary Figure 15 (**a**) Absorbance at 254 nm, (**b**) non-deconvoluted, and (**c**) deconvoluted MS data for bioconjugate **6**.

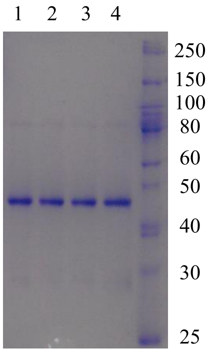

Supplementary Figure 16 SDS-PAGE analysis for bioconjugates **3**, **4**, **5**, and **6** (lanes 1–4 respectively)

Activity and stability of bioconjugate 6

Activity by enzyme-linked immunosorbent assay (ELISA)

Binding affinity to HER2 receptor was determined by ELISA. A 96-well plate was coated overnight at 4 °C with HER2 (100 μL of a 0.25 μg·mL^{-1} solution in PBS), including coating one row of wells with PBS only for negative controls. Next, coating solutions were removed and each well washed with PBS twice. Then, the wells were coated with a 1% BSA solution in PBS (200 μL) for 1 h at 21 °C. Then, the wells were washed with PBS three times. Solutions of Fab-Her **2** and trastuzumab Fab-Diet **6** in PBS with the following dilution series: 23 nM, 7.8 nM, 2.6 nM, 0.86 nM, 0.29 nM and 0.10 nM were prepared. Wells were coated with the dilution series solutions, including a PBS only and Fab-Her at 23 nM in the absence of HER2 as negative controls, and incubated for 2 h at room temperature. Then, the solutions were removed and the wells washed with 0.1% Tween 20 in PBS twice and with PBS three times. Detection antibody (100 μL of anti-human IgG, Fab-specific-HRP solution, prepared by taking 4 μL of a 1:5000 diluted solution and further diluting with 20 mL of PBS) was added and incubated for 1 h at room temperature. Then, the solutions were removed and the wells washed with 0.1% Tween 20 in PBS twice and with PBS three times. Finally, an OPD solution (100 μL of 0.5 mg·mL^{-1} OPD in phosphate-citrate buffer with sodium perborate, prepared by dissolving 1 capsule in 100 mL water) was added to each well. After *ca.* 2 min the reaction was stopped through addition of 4 M HCl (50 μL). Absorbance was measured at 490 nm. Absorbance was corrected by subtracting average of negative controls. See Figure 5b.

Stability under acidic pH

Fab-Diet **6** (50 μL, 30 μM, 1.4 mg·mL^{-1}) was buffer exchanged into acetate buffer (20 mM sodium acetate trihydrate, pH 3.1) by repeated diafiltration into fresh buffer using VivaSpin sample concentrators (GE Healthcare, 10000 MWCO) and was incubated at 37 °C for 24 h. Following this, analysis by LCMS revealed no trace of degradation of bioconjugate **6** (expected mass: 47820 Da, observed mass: 47822 Da).

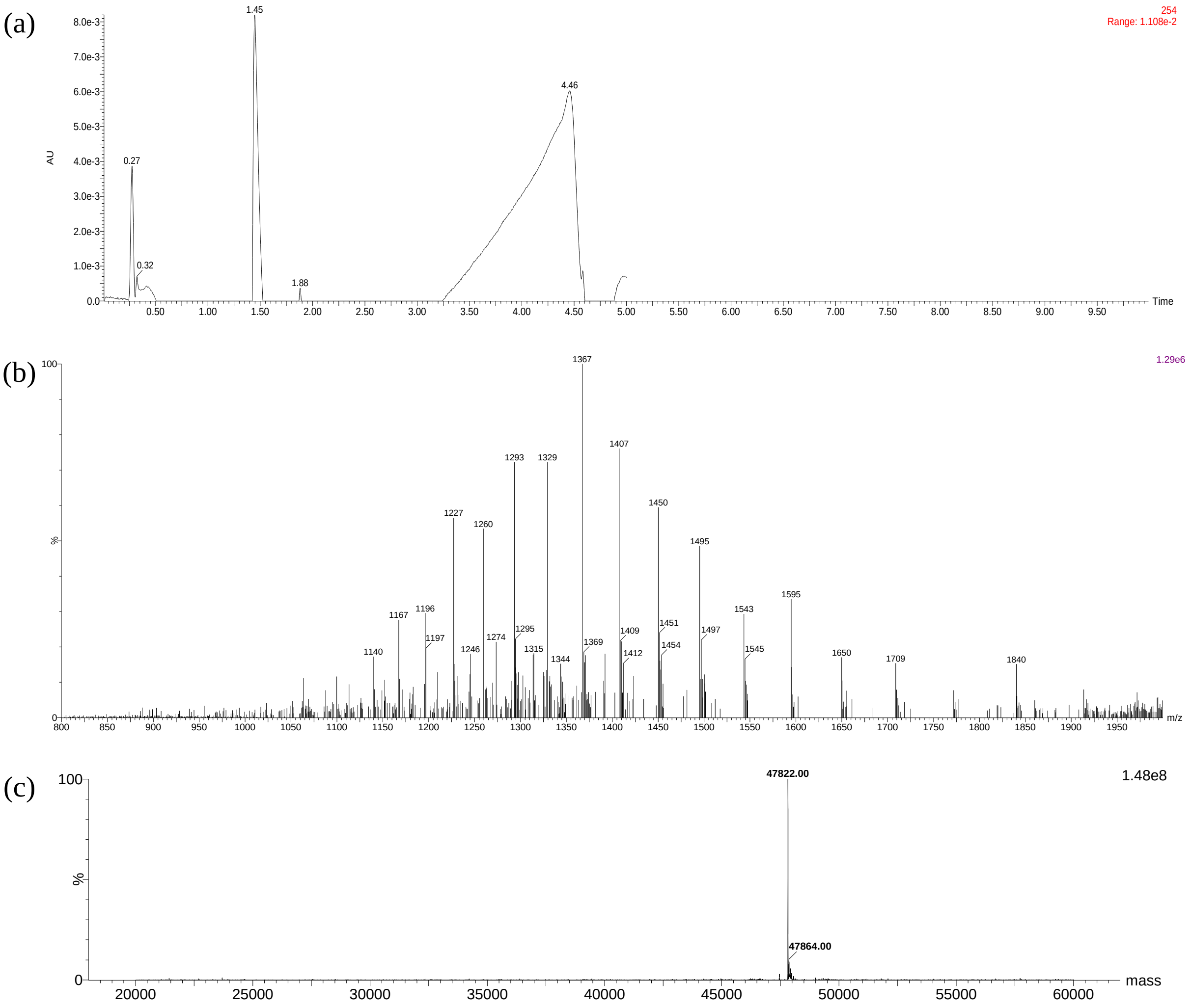

Supplementary Figure 17 (**a**) Absorbance at 254 nm, (**b**) non-deconvoluted, and (**c**) deconvoluted MS data of Fab-Diet **6** after 24 h at 37 °C in buffer pH 3.1.

Stability under basic pH

Fab-Diet **6** (50 µL, 30 µM, 1.4 mg·mL^{-1}) was buffer exchanged into BBS (pH 9) by repeated diafiltration into fresh buffer using VivaSpin sample concentrators (GE Healthcare, 10000 MWCO) and was incubated at 37 °C for 24 h. Following this, analysis by LCMS revealed no trace of degradation of bioconjugate **6** (expected mass: 47820 Da, observed mass: 47822 Da).

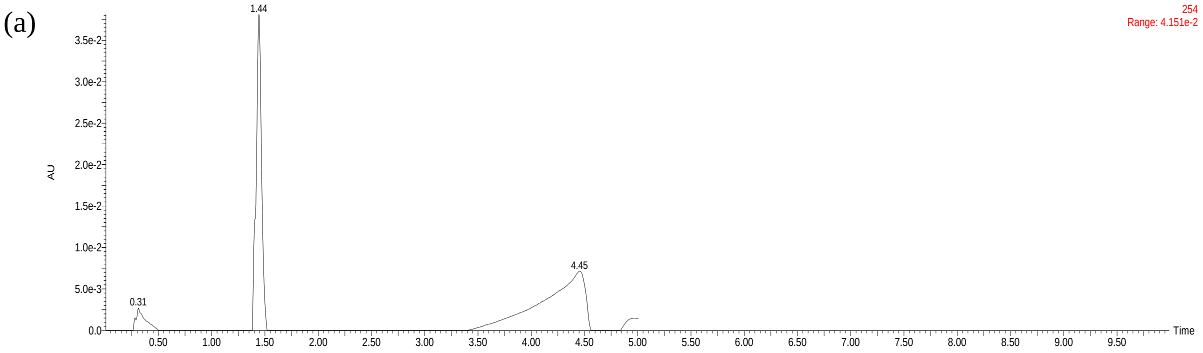

(b)
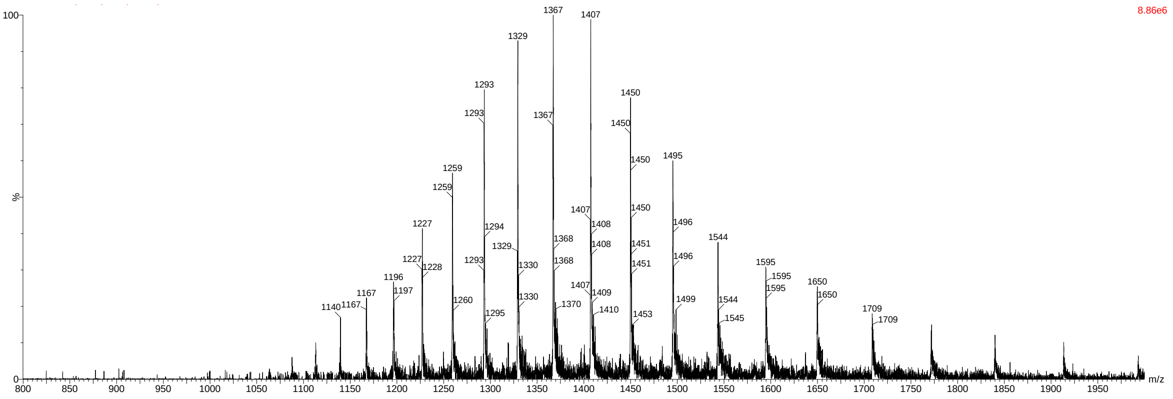

(c)
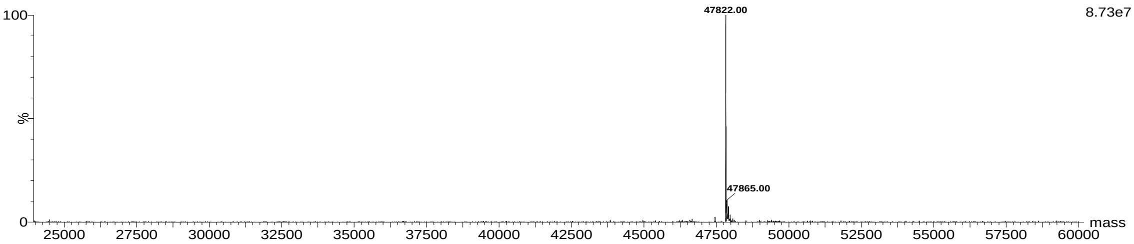

Supplementary Figure 18 (**a**) Absorbance at 254 nm, (**b**) non-deconvoluted, and (**c**) deconvoluted MS data of Fab-Diet **6** after 24 h at 37 °C in buffer pH 9.0.

Stability after 8 months at 4 °C

Fab-Diet **6** (50 µL, 30 µM, 1.4 mg·mL^{-1}) was buffer exchanged into PBS (pH 7.4) by repeated diafiltration into fresh buffer using VivaSpin sample concentrators (GE Healthcare, 10000 MWCO) and was incubated at 4 °C for 8 months. Following this, analysis by LCMS revealed no trace of degradation of bioconjugate **6** (expected mass: 47820 Da, observed mass: 47826 Da).

(a)
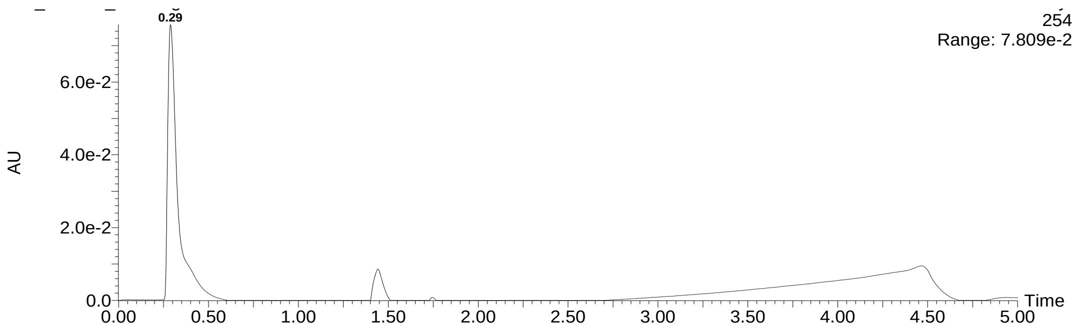

(b)

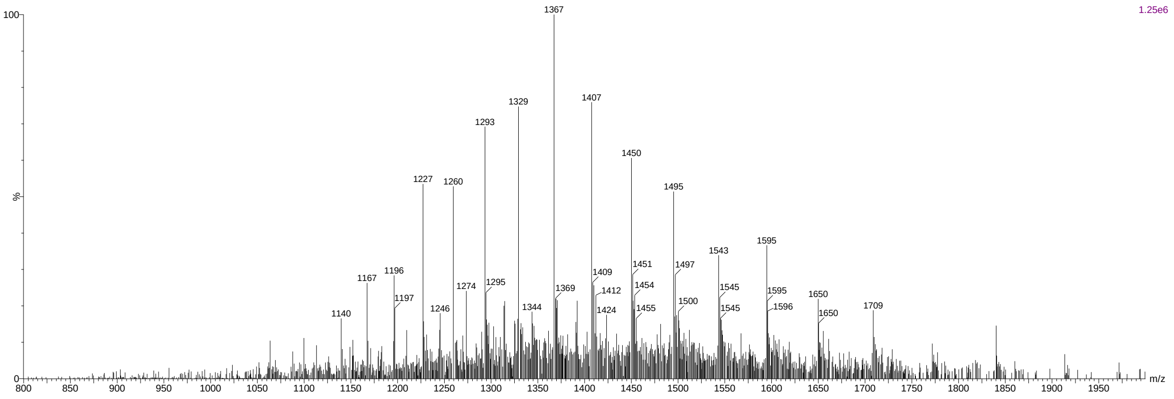

(c)

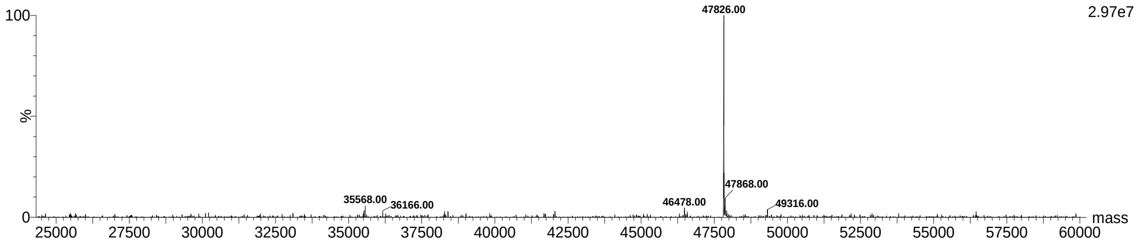

Supplementary Figure 19 (**a**) Absorbance at 254 nm, (**b**) non-deconvoluted, and (**c**) deconvoluted MS data of Fab-Diet **6** after eight months of storage at 4 °C in PBS.

Stability in blood plasma mimicking conditions

Fab-Diet **6** (200 μL, 40 μM, 1.9 mg·mL^{-1}) was buffer exchanged into a simulated body fluid solution (SBF) by repeated diafiltration into fresh buffer using VivaSpin sample concentrators (GE Healthcare, 10000 MWCO) then, human serum albumin (HSA) (final concentration 600 μM) and glutathione (GSH) (final concentration 20 μM) were added. The solution was incubated at 37 °C for 7 days. The reaction was monitored by SDS-PAGE and the analysis revealed no trace of reaction of bioconjugate **6** with GSH (expected resulting mass *ca.* 23 kDa) or with HSA (expected resulting mass *ca.* 90 kDa). See Figure 5c.

Stability control with native Herceptin and Fab-Her 2

The stability of Herceptin and Fab-Her **2** were also assessed under similar conditions. The results obtained are presented below.

(a)

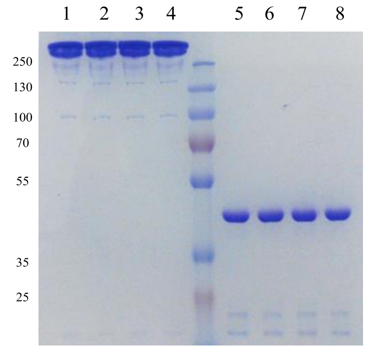

(b) (c)

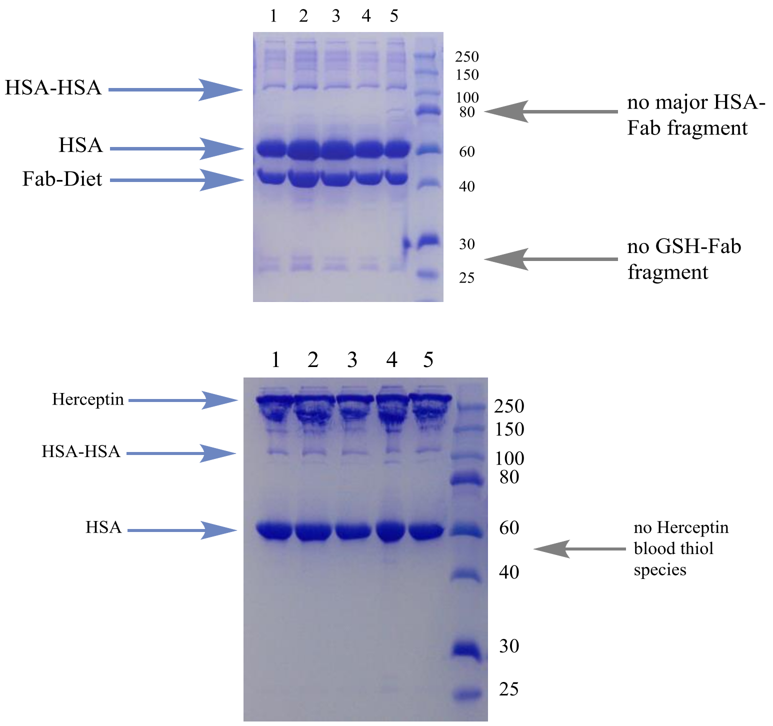

Supplementary Figure 20 (**a**) SDS-PAGE gel of Herceptin, Herceptin after two months of storage at 4 °C in PBS, Herceptin after 24 h at 37 °C in buffer pH 3.1, Herceptin after 24 h at 37 °C in buffer pH 9.0, Fab-Her **2**, Fab-Her **2** after two months of storage at 4 °C in PBS, Fab-Her **2** after 24 h at 37 °C in buffer pH 3.1, Fab-Her **2** after 24 h at 37 °C in buffer pH 9.0 (lanes 1–8 respectively), (**b**) SDS-PAGE gel following incubation of Fab-Her **2** in blood plasma mimicking conditions for 0, 1, 3, 5 and 7 days (lanes 1–5 respectively), and (**c**) SDS-PAGE gel following incubation of Herceptin in blood plasma mimicking conditions for 0, 1, 3, 5 and 7 days (lanes 1–5 respectively).

Formation of bioconjugate 7

To a solution of Fab-Her **2** (50 µL, 30 µM, 1.4 mg·mL^{-1}, 1 eq) in borate buffer (25 mM sodium borate, 25 mM NaCl, 0.5 mM EDTA, pH 8.0) was added TCEP (final concentration 90 µM, 3 eq) and the reaction mixture incubated at 37 °C for 90 min. After this time, was added a solution of Astra-PD **1** in DMF (final concentration 1.5 mM, 5 eq) and the reaction mixture incubated at 37 °C for 1 h. The excess reagents were then removed by repeated diafiltration into fresh buffer using VivaSpin sample concentrators (GE Healthcare, 10000 MWCO). Following this, analysis by LCMS revealed conversion to the desired trastuzumab Fab-Astra bioconjugate **3** (expected mass: 48212 Da, observed mass: 48211 Da). Then was added Dox-PEG$_4$-N$_3$ (5 eq from a 20 mM solution in DMF) and the reaction mixture incubated at 37 °C for 4 h. The excess reagents were then removed by repeated diafiltration into fresh PBS using VivaSpin sample concentrators (GE Healthcare, 10000 MWCO). Following this, analysis by LCMS revealed conversion to the desired trastuzumab Fab-Astra-Dox bioconjugate in an average >95% yield (expected mass: 49013 Da, observed mass: 49016 Da).

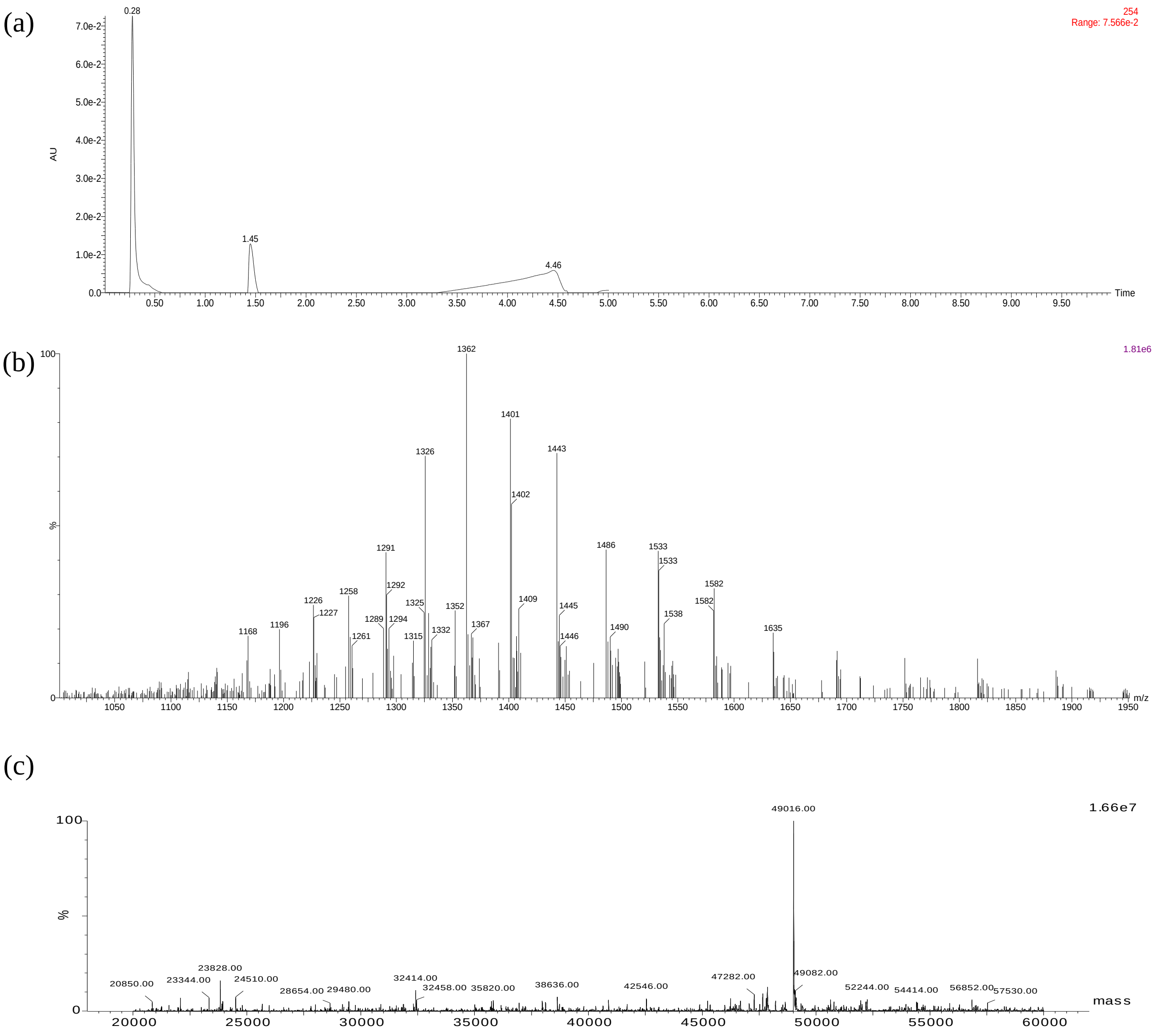

Supplementary Figure 21 (**a**) Absorbance at 254 nm, (**b**) non-deconvoluted, and (**c**) deconvoluted MS data for Fab-Astra-Dox.

Then, tris(3-hydroxypropyltriazolylmethyl)amine (THPTA) (500 μM), $CuSO_4$ (100 μM) was added followed by addition of PEG_{20k}-N_3 (2 eq) and sodium ascorbate (final concentration 2.5 mM) and the reaction mixture incubated at 37 °C for 16 h. Following this, analysis by SDS-PAGE gel revealed conversion to the desired trastuzumab Fab-Astra-Dox-PEG_{20k} bioconjugate **7**.

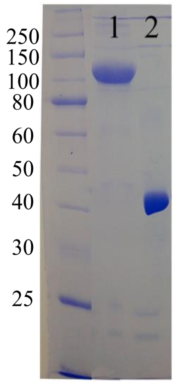

Supplementary Figure 22 SDS-PAGE analysis for bioconjugates Fab-Astra-Dox-PEG$_{20k}$ **7** and Fab-Astra-Dox (lanes 1 and 2 respectively).

Activity of bioconjugates 7 and 8

Enzyme-linked immunosorbent assay (ELISA)

Binding affinity to HER2 receptor was determined by ELISA. A 96-well plate was coated overnight at 4 °C with HER2 (Human HER2/ErbB2 Protein (His Tag) from Sino Biological) (100 μL of a 0.25 μg·mL^{-1} solution in PBS), including coating one row of wells with PBS only for negative controls. Next, coating solutions were removed and each well washed with PBS twice. Then, the wells were coated with a 1% BSA solution in PBS (200 μL) for 1 h at 21 °C. Then, the wells were washed with PBS three times. Solutions of trastuzumab mAb, Fab-Her **2**, Fab-Dox-PEG$_{20k}$ **7**, and Her-Dox-Cy5 **8** in PBS with the following dilution series: 30 nM, 10 nM, 3.3 nM, 1.1 nM, 0.37 nM and 0.12 nM were prepared. Wells were coated with the dilution series solutions, including a PBS only and Fab-Her at 30 nM in the absence of HER2 as negative controls, and incubated for 2 h at room temperature. Then, the solutions were removed and the wells washed with 0.1% Tween 20 in PBS twice and with PBS three times. Detection antibody (100 μL of anti-human IgG, Fab-specific-HRP solution, prepared by taking 4 μL of a 1:5000 diluted solution and further diluting with 20 mL of PBS) was added and incubated for 1 h at room temperature. Then, the solutions were removed and the wells washed with 0.1% Tween 20 in PBS twice and with PBS three times. Finally, an OPD solution (100 μL of 10 mg·20 mL^{-1} OPD in phosphate-citrate buffer with sodium perborate, prepared by dissolving 1 capsule in 100 mL water) was added to each well. After *ca.* 2 min the reaction was stopped through addition of 4 M HCl (50 μL). Absorbance was measured at 490 nm. Absorbance was corrected by subtracting average of negative controls.

To demonstrate that affinity/selectivity is not affected by conjugation, binding affinity to EGFR (HER1) receptor was determined by ELISA. Following the protocol described above with solutions of trastuzumab mAb, Fab-Her **2**, Fab-Dox-PEG$_{20k}$ **7**, Her-Dox-Cy5 **8** and cetuximab (as positive control) in PBS at 30 nM, the binding affinities were compared. The results obtained confirmed that the modifications do not alter selectivity.

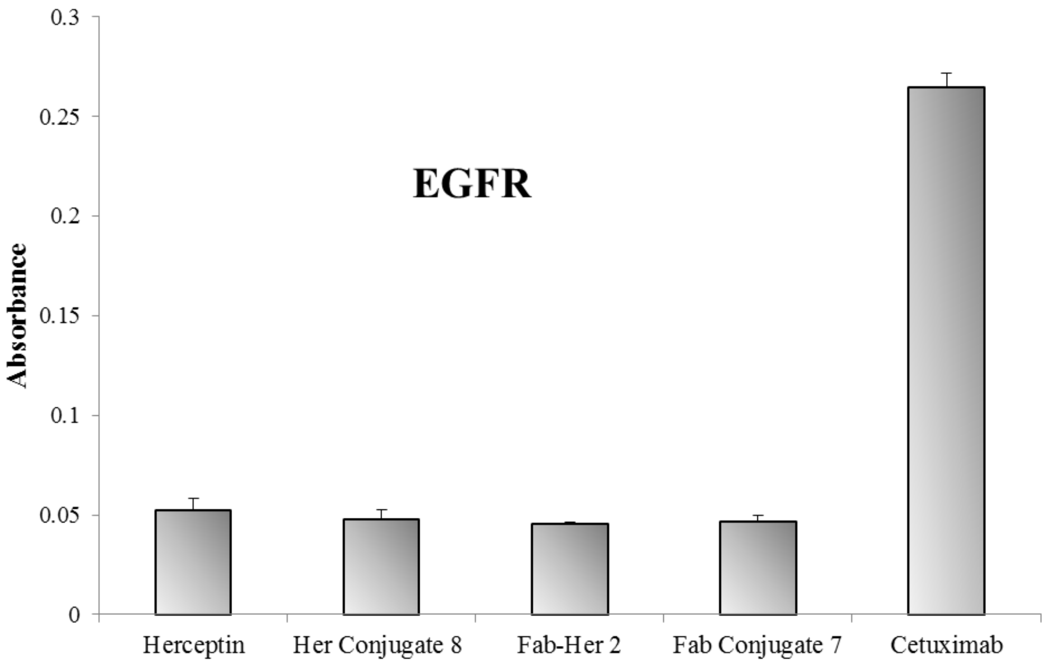

Supplementary Figure 23 Binding activity of Herceptin, Her-Astra-Dox-Cy5 **8**, Fab-Her **2**, Fab-Astra-Dox-PEG_{20k} **7**, and cetuximab (as positive control).

Bioconjugation reactions involving trastuzumab mAb

Formation of bioconjugate 8

To a solution of trastuzumab (100 µL, 50 µM, 7.3 mg·mL^{-1}, 1 eq) in borate buffer (25 mM sodium borate, 25 mM NaCl, 0.5 mM EDTA, pH 8.0) was added TCEP (final concentration 500 µM, 10 eq) and Astra-PD **1** in DMF (final concentration 1.0 mM, 20 eq) and the reaction mixture incubated at 4 °C for 6 h. The excess reagents were then removed by repeated diafiltration into fresh buffer using VivaSpin sample concentrators (GE Healthcare, 10000 MWCO). Following this, analysis by SDS-PAGE gel and UV-Vis revealed conversion to the desired Her-Astra bioconjugate with a PD-to-antibody ratio (PAR) of 4. Then was added Dox-PEG_4-N_3 (20 eq from a 20 mM solution in DMF) and the reaction mixture incubated at 37 °C for 4 h. The excess reagents were then removed by repeated diafiltration into fresh PBS using VivaSpin sample concentrators (GE Healthcare, 10000 MWCO). Following this, analysis by SDS-PAGE gel and UV-Vis revealed conversion to the desired Her-Astra-Dox bioconjugate with a doxorubicin-to-antibody ratio (DAR) of 4. Then, tris(3-hydroxypropyltriazolylmethyl)amine (THPTA) (1.25 mM), $CuSO_4$ (250 µM) was added followed by addition of Sulfo-Cy5-N_3 (10 eq) and sodium ascorbate (final concentration 5 mM) and the reaction mixture incubated at 37 °C for 16 h. The excess reagents were then removed by repeated diafiltration into fresh PBS with 2 mM EDTA (to remove residual copper ions) using VivaSpin sample concentrators (GE Healthcare, 10000 MWCO). Following this, analysis by SDS-PAGE gel and UV-Vis revealed conversion to the desired Her-Astra-Dox-Cy5 bioconjugate **8** with a fluorophore-to-antibody ratio (FAR) of 4.

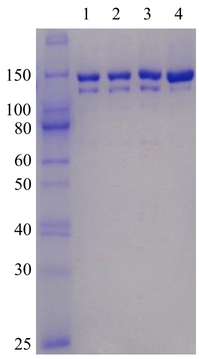

Supplementary Figure 24 SDS-PAGE analysis for Her-Astra, Her-Astra-Dox, Her-Astra-Dox-Cy5 **8** and Herceptin (lanes 1–4 respectively)

(a)

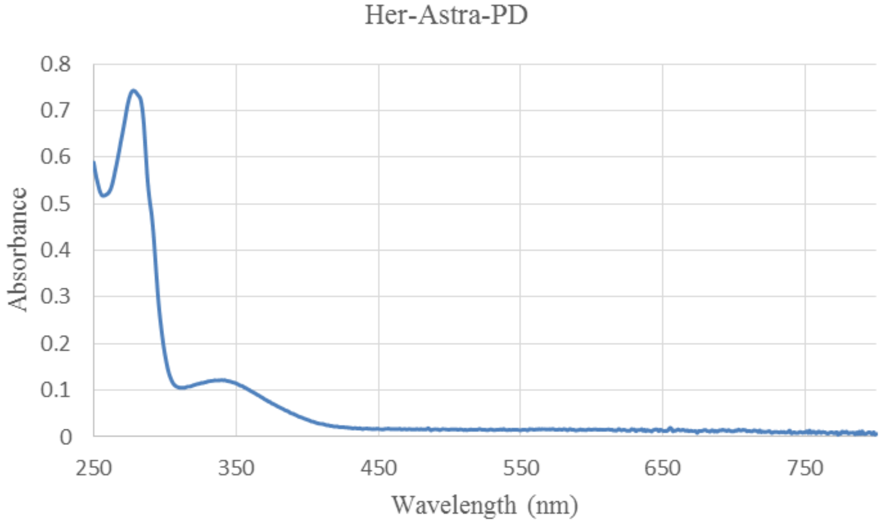

(b)

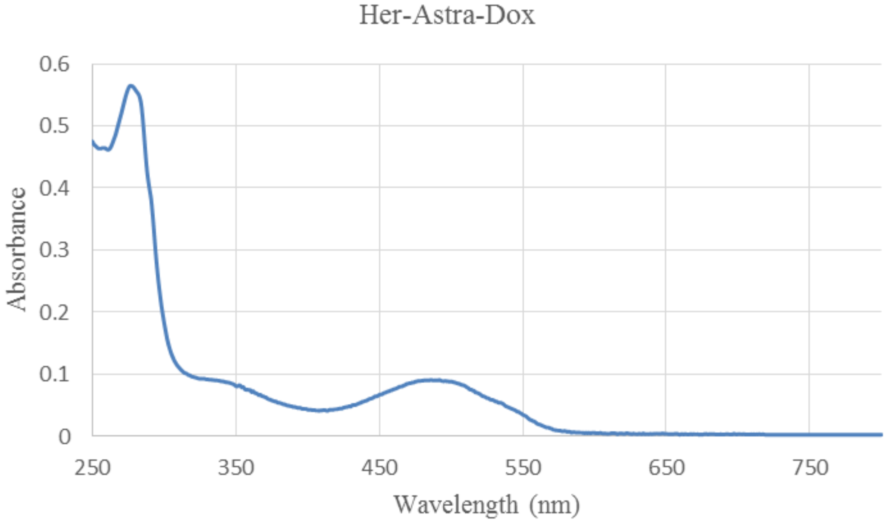

(c)

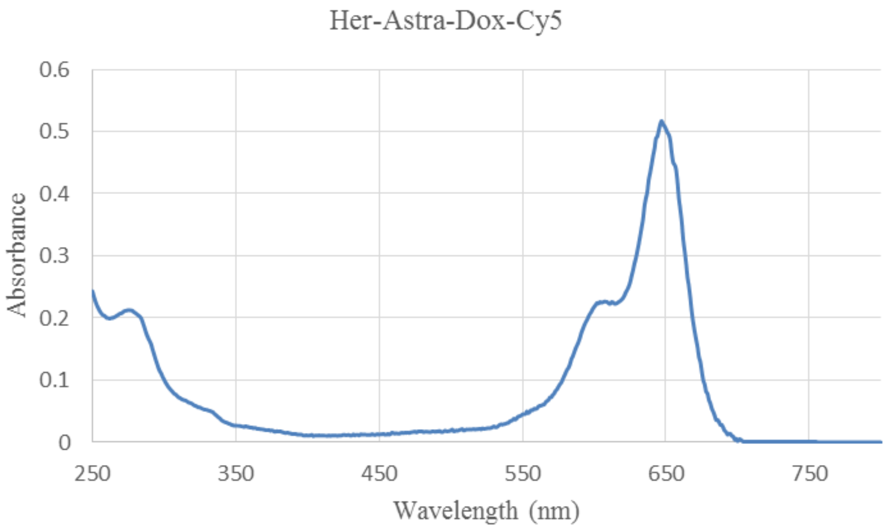

Supplementary Figure 25 UV-Vis trace for (**a**) Her-Astra, (**b**) Her-Astra-Dox, and (**c**) Her-Astra-Dox-Cy5 **8**.

Stability of bioconjugates 7 and 8

Stability under acidic pH, basic pH and after 2 months at 4 °C

Fab-Astra-Dox-PEG_{20k} **7** and Her-Astra-Dox-Cy5 **8** were subjected to the forcing conditions described above for Fab-Diet **6**. The results obtained are presented below.

(a)

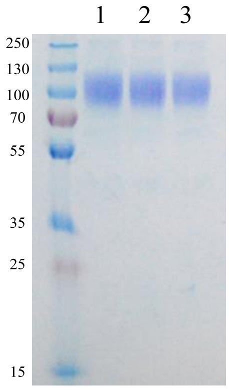

(b)

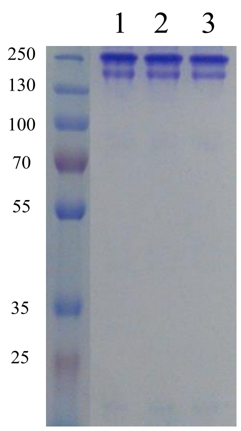

Supplementary Figure 26 SDS-PAGE gel of (**a**) Fab-Astra-Dox-PEG$_{20k}$ **7** after two months of storage at 4 °C in PBS, Fab-Astra-Dox-PEG$_{20k}$ **7** after 24 h at 37 °C in buffer pH 3.1 and Fab-Astra-Dox-PEG$_{20k}$ **7** after 24 h at 37 °C in buffer pH 9.0 (lanes 1–3 respectively), (**b**) Her-Astra-Dox-Cy5 **8** after two months of storage at 4 °C in PBS, Her-Astra-Dox-Cy5 **8** after 24 h at 37 °C in buffer pH 3.1 and Her-Astra-Dox-Cy5 **8** after 24 h at 37 °C in buffer pH 9.0 (lanes 1–3 respectively).

Stability in blood plasma mimicking conditions

Fab-Astra-Dox-PEG$_{20k}$ **7** and Her-Astra-Dox-Cy5 **8** were incubated in blood plasma mimicking conditions as described above for Fab-Diet **6**. The results obtained are presented below.

(a)

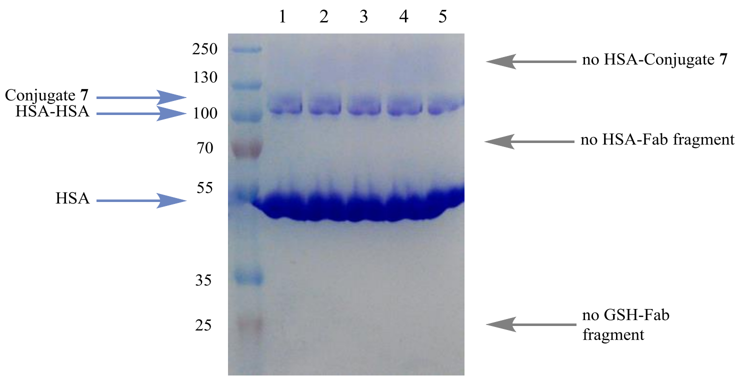

(b)

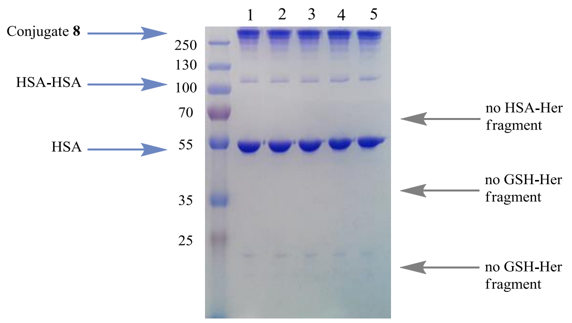

Supplementary Figure 27 (**a**) SDS-PAGE gel following incubation of Fab-Astra-Dox-PEG$_{20k}$ **7** in blood plasma mimicking conditions for 0, 1, 3, 5 and 7 days (lanes 1–5 respectively) and (**b**) SDS-PAGE gel following incubation of Her-Astra-Dox-Cy5 **8** in blood plasma mimicking conditions for 0, 1, 3, 5 and 7 days (lanes 1–5 respectively).

Formation of AlexaFluor488 bioconjugates for internalisation analysis by confocal microscopy

Fab-PD-AlexaFluor488 conjugate

To a solution of Fab-Her **2** (500 µL, 30 µM, 1.4 mg·mL^{-1}, 1 eq) in borate buffer (25 mM sodium borate, 25 mM NaCl, 0.5 mM EDTA, pH 8.0) was added TCEP (final concentration 90 µM, 3 eq) and the reaction mixture incubated at 37 °C for 90 min. After this time, was added a solution of Astra-PD **1** in DMF (final concentration 1.5 mM, 5 eq) and the reaction mixture incubated at 37 °C for 1 h. The excess reagents were then removed by repeated diafiltration into fresh buffer using VivaSpin sample concentrators (GE Healthcare, 10000 MWCO). Following this, analysis by LCMS revealed conversion to the desired trastuzumab Fab-Astra bioconjugate **3** (expected mass: 48212 Da, observed mass: 48211 Da). Then was added AlexaFluor488-Azide (Molecular Probes®) (5 eq from a 10 mM solution in DMSO) and the reaction mixture incubated at 37 °C for 4 h. The excess reagents were then removed by repeated diafiltration into fresh PBS using VivaSpin sample concentrators (GE Healthcare, 10000 MWCO). Following this, analysis by LCMS revealed conversion to the desired trastuzumab Fab-PD-AlexaFluor488 bioconjugate in a >95% yield (expected mass: 48871 Da, observed mass: 48871 Da).

(a)

(b)

(c)

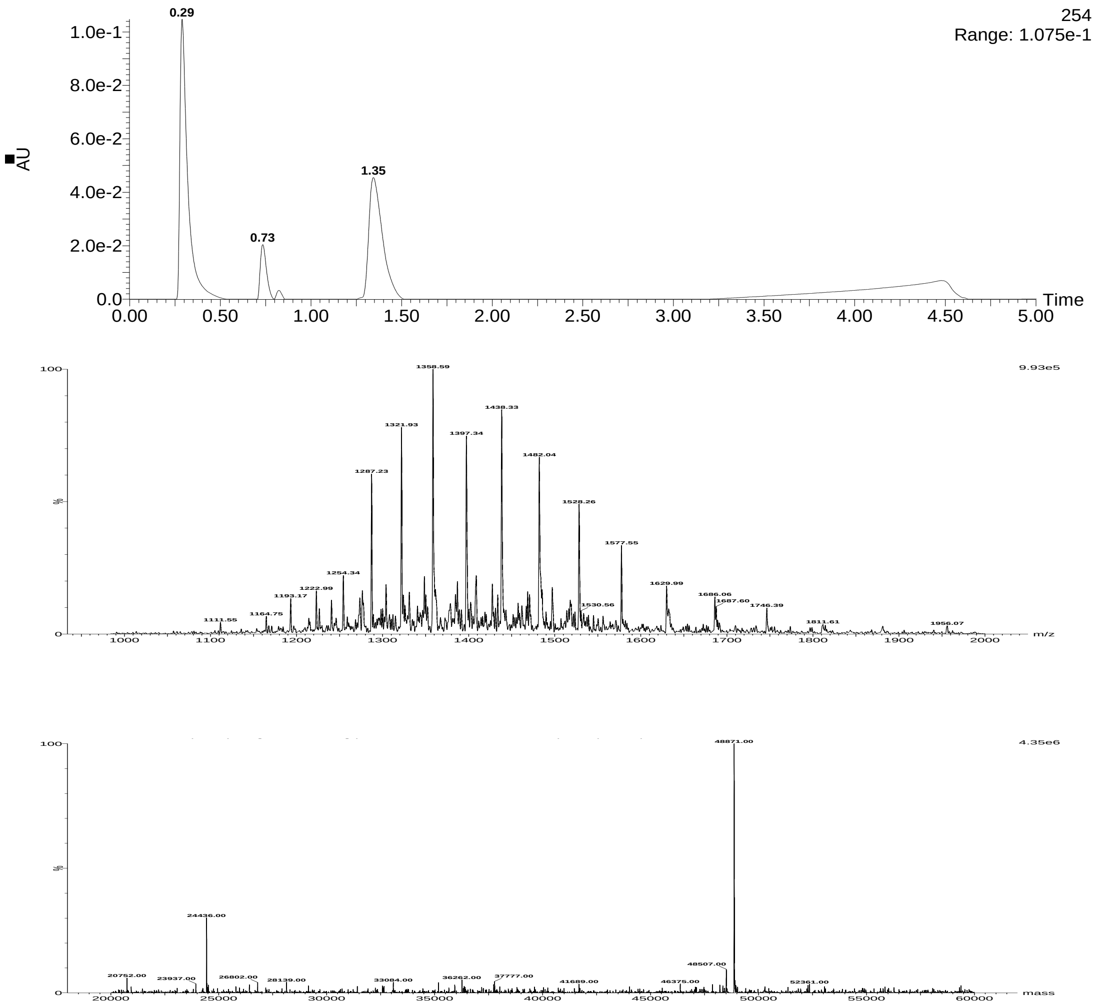

Supplementary Figure 28 (a) Absorbance at 254 nm, (b) non-deconvoluted, and (c) deconvoluted MS data for Fab-PD-AlexaFluor488.

Her-PD-AlexaFluor488 conjugates

To a solution of trastuzumab (300 µL, 50 µM, 7.3 mg·mL^{-1}, 1 eq) in borate buffer (25 mM sodium borate, 25 mM NaCl, 0.5 mM EDTA, pH 8.0) was added TCEP (final concentration 500 µM, 10 eq) and Astra-PD **1** in DMF (final concentration 1.0 mM, 20 eq) and the reaction mixture incubated at 4 °C for 6 h. The excess reagents were then removed by repeated diafiltration into fresh buffer using VivaSpin sample concentrators (GE Healthcare, 10000 MWCO). Following this, analysis by SDS-PAGE gel and UV-Vis revealed conversion to the desired Her-Astra bioconjugate with a PD-to-antibody ratio (PAR) of 4. Then was added AlexaFluor488-Azide (Molecular Probes®) (20 eq from a 10 mM solution in DMSO) and the reaction mixture incubated at 37 °C for 4 h. The excess reagents were then removed by repeated diafiltration into fresh PBS using VivaSpin sample concentrators (GE Healthcare, 10000 MWCO). Following this, analysis by SDS-PAGE gel and UV-Vis revealed conversion to the desired Her-PD-AlexaFluor488 bioconjugate with a fluorophore-to-antibody ratio (FAR) of 4.

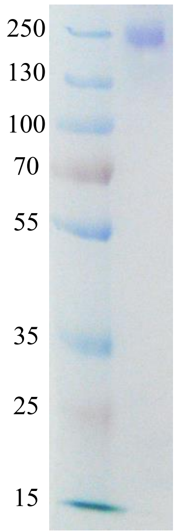

Supplementary Figure 29 SDS-PAGE gel of Her-PD-AlexaFluor488 conjugate.

Reduction control

To a solution of trastuzumab (100 µL, 50 µM, 7.3 mg·mL^{-1}, 1 eq) in borate buffer (25 mM sodium borate, 25 mM NaCl, 0.5 mM EDTA, pH 8.0) was added TCEP (final concentration 500 µM, 10 eq) and the reaction mixture incubated at 37 °C for 90 min. Following this, analysis by LCMS and by SDS-PAGE gel revealed conversion to the desired reduced Herceptin (expected masses (non-glycosylated Fc): 23444 and 49237 Da, observed mass (glycosylated Fc): 23444, 50605 and 50765 Da (+ galactose)).

To a solution of Fab-Her **2** (50 µL, 30 µM, 1.4 mg·mL^{-1}, 1 eq) in borate buffer (25 mM sodium borate, 25 mM NaCl, 0.5 mM EDTA, pH 8.0) was added TCEP (final concentration 90 µM, 3 eq) and the reaction mixture incubated at 37 °C for 90 min. Following this, analysis by LCMS and by SDS-PAGE gel revealed conversion to the desired heavy and light chains only (*i.e.* the reduced fragment) (expected masses: 23444 and 24208 Da, observed mass: 23449 and 24209 Da.

Selectivity control in the absence of TCEP

To a solution of Fab-Her **2** (50 μL, 30 μM, 1.4 mg·mL^{-1}, 1 eq) in borate buffer (25 mM sodium borate, 25 mM NaCl, 0.5 mM EDTA, pH 8.0) was incubated at 37 °C for 90 min. After this time, was added a solution of Astra-PD **1** in DMF (final concentration 1.5 mM, 5 eq) and the reaction mixture incubated at 37 °C for 1 h. The excess reagents were then removed by repeated diafiltration into fresh buffer using VivaSpin sample concentrators (GE Healthcare, 10000 MWCO). Following this, analysis by LCMS revealed that no reaction occurred on Fab-Her **2** (expected mass: 47652 Da, observed mass: 47656 Da).

Supplementary References

1. Rasmussen, S. C. 6,6 '-Dibromo-4,4 '-di(hexoxymethyl)-2,2 '-bipyridine: A new solubilizing building block for macromolecular and supramolecular applications. *J. Org. Chem.* **71**, 4696–4699 (2006).

2. Castañeda, L., Maruani, A., Schumacher, F. F., Miranda, E., Chudasama, V., Chester, K. A. *et al.* Acid-cleavable thiomaleamic acid linker for homogeneous antibody-drug conjugation. *Chem. Commun.* **49**, 8187–8189 (2013).

3. Frew, A. J. & Proctor, G. R. Ring-Expansion of Carbocyclic Beta-Ketoesters with Acetylenic Esters. *J. Chem. Soc. Perk. T. 1*, 1245–1250 (1980).

4. Schultz, M. K., Parameswarappa, S. G. & Pigge, F. C. Synthesis of a DOTA-Biotin Conjugate for Radionuclide Chelation via Cu-Free Click Chemistry. *Org. Lett.* **12**, 2398–2401 (2010).

5. Dai, C., Cazares, L. H., Wang, B., Wang, L., Chu, Y., WANG, S. L. *et al.* Cellular recognition conjugates and methods of use for the histological analysis of cancer tissue using maldi-ms imaging. Patent application WO2013/013130 (2013).

6. Schwabacher, A. W., Lane, J. W., Schiesher, M. W., Leigh, K. M. & Johnson, C. W. Desymmetrization reactions: Efficient preparation of unsymmetrically substituted linker molecules. *J. Org. Chem.* **63**, 1727–1729 (1998).

7. Chudasama, V., Smith, M. E. B., Schumacher, F. F., Papaioannou, D., Waksman, G., Baker, J. R. *et al.* Bromopyridazinedione-mediated protein and peptide bioconjugation. *Chem. Commun.* **47**, 8781–8783 (2011).
